# The crucial role of beta-catenin in the osteoprotective effect of semaglutide in an ovariectomized rat model of osteoporosis

**DOI:** 10.1007/s00210-024-03378-z

**Published:** 2024-09-10

**Authors:** Mohannad Hakam Hamed Abo-Elenin, Rehab Kamel, Shahira Nofal, Amany Ali Eissa Ahmed

**Affiliations:** Pharmacology and Toxicology Department, Faculty of Pharmacy, Helwan University, Ein Helwan, Cairo City, Egypt

**Keywords:** Osteoporosis, Ovariectomy, Semaglutide, β-catenin, Wnt signaling

## Abstract

Postmenopausal osteoporosis is a common chronic medical illness resulting from an imbalance between bone resorption and bone formation along with microarchitecture degeneration attributed to estrogen deficiency and often accompanied by other medical conditions such as weight gain, depression, and insomnia. Semaglutide (SEM) is a recently introduced GLP-1 receptor agonist (GLP-1RA) for the treatment of obesity and type 2 diabetes mellitus by mitigating insulin resistance. It has been discovered that the beneficial effects of GLP-1 are associated with alterations in lipolysis, adipogenesis, and anti-inflammatory processes. GLP-1 analogs transmit signals directly to adipose tissue. Mesenchymal stem cells (MSCs) are multidisciplinary cells that originate from bone marrow, migrate to injury sites, and promote bone regeneration. MSCs can differentiate into osteoblasts, adipose cells, and cartilage cells. Our aim is to investigate the role of semaglutide on bone formation and the Wnt signaling pathway. Osteoporosis was induced in female rats by ovariectomy, and the ovariectomized rats were treated with alendronate as standard treatment with a dose of 3 mg/kg orally and semaglutide with two doses (150 mcg/kg and 300 mcg/kg) S.C. for 10 successive weeks. Semaglutide ameliorates bone detrimental changes induced by ovariectomy. It improves bone microarchitecture and preserves bone mineral content. Semaglutide ameliorates ovariectomy-induced osteoporosis and increases the expression of β-catenin, leading to increased bone formation and halted receptor activator of nuclear factor kappa-Β ligand (RANKL’s) activation. Semaglutide can be used as a potential prophylactic and therapeutic drug against osteoporosis, possibly by activating Wnt signaling and decreasing bone resorption.

**Graphical Abstract:**

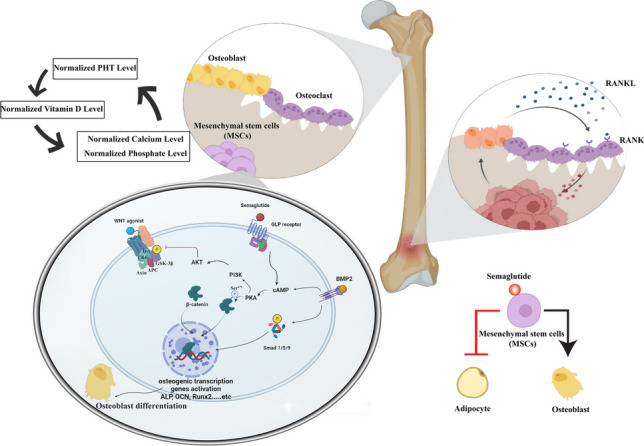

**Supplementary Information:**

The online version contains supplementary material available at 10.1007/s00210-024-03378-z.

## Introduction

Osteoporosis is a common metabolic skeletal disease characterized by low bone mineral density and degeneration of bone tissue microarchitecture that affects specific categories of people, especially the elderly and postmenopausal women (Xu et al. 2019). In postmenopausal women in Egypt, the prevalence of osteopenia and osteoporosis is 53.9% and 28.4%, respectively. In addition, recent research found that 26% of men have osteopenia and 21.9% have osteoporosis (El Miedany et al. 2021). The worldwide occurrence of osteoporosis is 18.3%, and the frequency among postmenopausal women and men was estimated to be 23.1% and 11.7%, respectively (Salari et al. 2021).

Osteoporosis is a silent disease that the patient is unaware of until the fracture of the bone occurs (Sozen et al. 2017). Postmenopausal osteoporosis is characterized by increased body weight, fat gain, and deterioration of bone quality leading to fragility fractures (N. Wawrzyniak et al. 2021).

Bone integrity is maintained by the remodeling process which includes bone resorption and bone formation (A. Wawrzyniak & Balawender 2022). Disruption of this balance by increasing bone resorption leads to decreased bone mass and osteoporosis (Abo-Elenin et al. 2024; Rowe et al. 2023). After menopause, estrogen deficiency leads to an increase in osteoclast recruitment and a decrease in osteoclast apoptosis. but exogenous estrogen replacement in post-menopausal women was found to have several adverse side effects, therefore this treatment is not recommended (Rajfer et al. 2019).

Recently, the Wnt signaling pathway has drawn significant attention in bone biology (Liu et al. 2013a; Regard et al. 2012). Specific bone pathologies result from abnormal Wnt signaling pathways such as sclerosteosis (Vlashi et al. 2022). The Wnt signaling pathway has an important role in the developmental osteogenic differentiation of mesenchymal stem cells (MSCs). MSCs are multipotent progenitors that can differentiate into other types of tissues, such as bone, cartilage, and fat (Liu et al. 2013b). The proper balance of osteogenic and adipogenic formation is essential for proper bone microarchitecture (Kawai et al. 2012). Therefore, as an effective therapy for bone disorders, induction of MSCs into the osteogenic lineage may promote normal bone formation (Liu et al. 2013b).

β-catenin has multiple roles during bone formation. In the early stages of bone tissue damage, β-catenin modulates the ratio of osteoblasts and chondrocytes in the callus formed by pluripotent MSCs (Bao et al. 2017). Also, β-catenin promotes osteoblast development and matrix formation during the healing process of bones (Wang et al. 2017).

Wnt signaling processes cause β-catenin to accumulate and translocate into the nucleus (Enzo et al. 2015). β-catenin’s roles encompass both facilitating cell adhesion through adherens junctions and regulating gene expression in response to Wnt signaling, highlighting its critical importance in signaling pathways and bone biology (Baron and Kneissel 2013; Enzo et al. 2015). Intranuclear accumulation of β-catenin activates transcription factors that target specific genes that mediate cellular development and osteogenesis (Cadigan and Waterman 2012; Houschyar et al. 2019). Without Wnt ligands, cytoplasmic β-catenin is degraded by a multiprotein complex consisting of Axin, casein kinase 1 (CK1), adenomatous polyposis coli (APC), and glycogen synthase kinase-3 (GSK-3) (Stamos and Weis 2013). When Wnt binds to its receptors, the intracellular protein, Dvl, is activated. Dvl transmits the receptor complex’s membrane signal to inhibit GSK-3b leading to the collapse of the β-catenin destruction complex (González-Sancho et al. 2004; Medina and Wandosell 2011). As a result, β-catenin is not degraded by the proteasome and can accumulate in the cytoplasm and translocate to the nucleus. Intranuclear β-catenin binds T-cell factor/lymphoid enhancer factor (TCF/LEF) family members and activates the transcription of genes involved in bone formation (Ma and Hottiger 2016).

Semaglutide is a recent GLP-1 analog with a prolonged half-life of approximately 1 week, that is widely used for the treatment of type 2 diabetes and obesity (Anam et al. 2022; Rubino et al. 2021). There is a strong association between GLP-1 agonists and Wnt signaling pathways. It has been documented that GLP-1 is implicated in significant biological functions by binding to the GLP-1 receptor, a cyclic adenosine monophosphate (cAMP)-linked G-protein-coupled receptors found in the cells of the pancreas and numerous extra-pancreatic tissues (Aoyama et al. 2014). During the osteogenic differentiation, a higher expression of the GLP-1 receptor gene was observed. This finding suggests that GLP-1 may be involved in the osteogenic differentiation of bone tissue (Jeon et al. 2014). Since osteoporosis and obesity are two diseases that affect a vast majority of postmenopausal women concomitantly, using a single drug that is Food and Drug Administration (FDA) approved for obesity to prevent and manage both osteoporosis and obesity could improve the quality of life and decrease the complications and medical costs that occur as a result of both diseases.

This study aims to investigate the possible ameliorating effects of semaglutide on bone mineral density (BMD), the possible mechanism, and its effect on the microarchitecture of the bone in ovariectomized rat models.

## Material and methods

### Drugs and chemicals

Semaglutide was purchased from Novo Nordisk, Bagsvaerd, Denmark, and alendronate was purchased from Sigma-Aldrich (St. Louis, MO, USA). All used chemicals were of high grade and purchased from standard commercial supplies.

## Experimental design

Thirty adult female Sprague–Dawley rats (150–170 gm) aged about 6 weeks were purchased from the breeding unit of the Egyptian Organization of the Biological Product and Vaccine (Helwan, Egypt). They were housed under controlled environmental conditions at a constant temperature (25 ± 2 °C) with free access to a standard pellets diet and drinking water for 1 week before experimentation. Animal body weight was recorded once weekly till the end of the experiment using a lab scale (ViBRA SJ-6200 Precision Digital Balance (Shinko Denshi Co., Japan)).

Animal care and experimental procedures were approved by the research ethics committee, Faculty of Pharmacy, Helwan University (protocol number: 16A2022).

## Experimental procedure

The rats were randomly allocated into five groups as follows:

- The control group underwent a sham operation and received subcutaneous (S.C.) injections of 44 mM sodium phosphate dibasic, 70 mM NaCl + 0.007% Tween 20 for 10 weeks once weekly.
- The osteoporotic group underwent ovariectomy and received S.C. injections of 44 mM sodium phosphate dibasic, 70 mM NaCl + 0.007% Tween 20 for 10 weeks once weekly.
- The alendronate group underwent ovariectomy and received oral alendronate (3 mg/kg) dissolved in 0.9% normal saline weekly for 10 weeks. Alendronate was used as a comparative standard.
- The SEM 150 group underwent ovariectomy and received S.C. injections (150 mcg/kg) of semaglutide once weekly for 10 weeks.
- The SEM 300 group underwent ovariectomy and received S.C. injections (300 mcg/kg) (Li et al. 2020) of semaglutide once weekly for 10 weeks.

Sham operation and ovariectomy were performed after the 1st week of habituation.

For the surgical procedure, rats were anesthetized using ketamine (70 mg/kg, intramuscular (I.M.)) and xylazine (8 mg/kg, I.M.) (Salazar et al. 2015), and the skin was cleaned with povidone-iodine and alcohol.

All surgical tools were sterilized before surgery. Hair was shaved, ovariectomy was done by double (bilateral) dorsolateral skin incisions (Kumar et al. 2012; Sophocleous & Idris 2014), and the ovary was resected (Yousefzadeh et al. 2020a).

After 12 weeks, all rats underwent a dual-energy X-ray absorptiometry (DEXA) scan using a Hologic QDR 4500 instrument (Hologic, Waltham, MA) which was utilized with a specialized software (version V819a) and a rat-specific internal standard then sacrificed by cervical dislocation. The blood and the femurs of the rats were collected for further analysis. The left femurs were removed from the adjacent tissues and frozen at − 80 °C, and the right femurs were preserved in 10% neutral buffered formalin for histological study.

## Biochemical analysis

The serum was isolated by centrifugation at 2000 × g for 15 min using the Megafuge® 8R Centrifuge (Thermo Scientific, Waltham, Massachusetts, USA). The supernatant was collected and stored at − 20 °C for further analysis of the bone formation, resorption marker, and inflammatory cytokines in rat serum. The levels of estradiol (cat no. CSB-E05110r), calcitonin (cat no. CSB-E05132r), bone-specific alkaline phosphatase (BALP) (cat no. CSB-E11914m), osteocalcin (cat no. CSB-E05129r) (Cusabio. Houston, USA), parathyroid hormone (PTH) (cat no. MBS2019938), vitamin D (cat no. MBS580159), RANKL (cat no. MBS268706), pyridinoline (PYD) (cat no. MBS720260) (MyBioSource, San Diego, CA), and interleukin-6 (IL-6) (cat no. E-EL-R0015) (Elabscience. Wuhan, China) were measured by enzyme-linked immunosorbent assay (ELISA) kits. Calcium (Ca^2+^) (cat no. E-BC-K103-M), phosphate (cat no. E-BC-K245-S) (Elabscience. Wuhan, China), creatinine (cat no. CRE106120), and urea (cat no. URE118120) (Biomed, Badr City; Egypt) were measured by colorimetry kits.

The bone tissue homogenates were used to measure the cAMP (cat no. MBS2700004) (MyBioSource, San Diego, CA).

## Gene expression of GLP1-R

The Thermo Scientific kit was used to extract total RNA from the bone of each sample. Individual samples of total RNA at 1 µg were employed to create single-strand cDNA using a kit (Thermo Fisher Scientific, USA). The real-time RT-PCR thermal cycling technique comprises three sequential steps. The protocol consists of an initial denaturation stage at 95 °C for 30 s, followed by 40 cycles of denaturation at 95 °C for 5 s. The last step involves annealing and extension at 60 °C for 20 s. The cycle threshold (Ct) values of the GLP1-R target genes were normalized to the Ct values of the β-actin housekeeping gene. The target gene primer sequence was as follows.

### GLP1R

Forward 5′-TCAGAGACGGTGCAGAAATG-3′.

Reverse 5′-CAGCTGACATTCACGAAGGA-3′.

### β-actin

Forward 5′-CCCATCTATGAGGGTTACGC-3′.

Reverse 5′-TTTAATGTCACGCACGATTTC-3′.

### Western blot analysis

Proteins were extracted and estimated from the bones’ samples using RIPA lysis buffer (150 mM NaCl, 0.1% Triton X-100, 0.5% sodium deoxycholate, 0.1% SDS (sodium dodecyl sulfate), 50 mM Tris–HCl, pH was adjusted at 8.0, 0.4 µl protease inhibitor buffer, 2.5 µl phosphatase inhibitor) from BufferBio BASIC Inc. (Markham Ontario L3R 8T4 Canada) and the Bradford protein assay kit (BIO BASIC Inc.), respectively, separated on a sodium dodecyl sulfate–polyacrylamide gel by electrophoresis and transferred to polyvinylidene difluoride membrane using the Bio-Rad Trans-Blot Turbo instrument. The membranes were incubated with the primary antibodies against p-β-catenin (Ser675), bone morphogenetic protein-2 (BMP-2), AKT, p-AKT, protein kinase A (PKA), and p-GSK3β; rinsed in Tris-buffered saline with Tween 20 (TBST) buffer; incubated with goat anti-rabbit HRP-conjugated secondary antibodies solution (Novus Biologicals, Littleton, CO, USA); and then washed in TBST buffer.

The chemiluminescent substrate (Clarity™ Western ECL substrate—BIO-RAD, USA cat# 170–5060) was applied to the blot. The protein band was visualized using a CCD camera-based imager. Image analysis software was used to read the band intensity of the target proteins against the control sample after normalization by beta-actin on the Chemi Doc MP imager.

### Histopathological examination

Femoral bone samples were fixed in 10% neutral buffered formalin for 48 h. Then decalcified by using the Cal-X II (Fisher Scientific) for 15 days; then, samples were processed using serial grades of ethanol and cleared in xylene followed by infiltration and embedding in a Paraplast tissue-embedding media. Five-micron tissue sections were made by rotatory microtome and mounted on glass slides for hematoxylin and eosin (H&E) staining and general light microscopic histological examination of tissue samples as well as Masson’s Goldners trichrome staining utilized for differentiation of mineralized and non-mineralized areas in bone matrix.

### Histomorphometric analysis

Six non-overlapping fields were randomly selected and scanned from each bone sample for the determination of mean trabecular bone area percentage, mean trabecular bone width in H&E-stained tissue sections, and relative bone area percentage of non-mineralized bone (osteoid) in Masson’s Goldner trichrome-stained samples. All light microscopic examinations and data were obtained by using the Leica Application module for histological analysis attached to the full HD microscopic imaging system (Leica Microsystems GmbH, Germany).

### Statistical analysis

Data were analyzed using the GraphPad Prism (version 9.1.0) (GraphPad Software Inc., San Diego, CA, USA) statistical analysis software, and results were shown as mean ± SD. The normality of the data was performed using the Kolmogorov–Smirnov test, and it passed; then, a test of significance between the groups was carried out using one-way analysis of variance (ANOVA) followed by Tukey–Kramer’s multiple comparisons post hoc test. In GLP-1R mRNA relative expression and western blot analysis relative protein expression, the test of significance between the groups was carried out using a non-parametric Kruskal–Wallis test. *P* < 0.05 was considered significant.

## Result

### Effect of ovariectomy on estradiol level

At the beginning of the experiment, the difference between the body weight of all rats was non-significant. In the 12th week, the osteoporotic group and alendronate group showed a significant increase in body weight compared to the control group, SEM 150 group, and SEM 300 group showed a significant decrease in body weight compared to the control group, from the beginning of the experiment, the amount of food which was 250 gm of standard pellets was added daily to all groups, and the next day the remaining amount was measured. The semaglutide group’s remaining amount was half food (125 gm) while the osteoporotic and alendronate groups had finished all the food (250 gm).

**Fig. 1:**
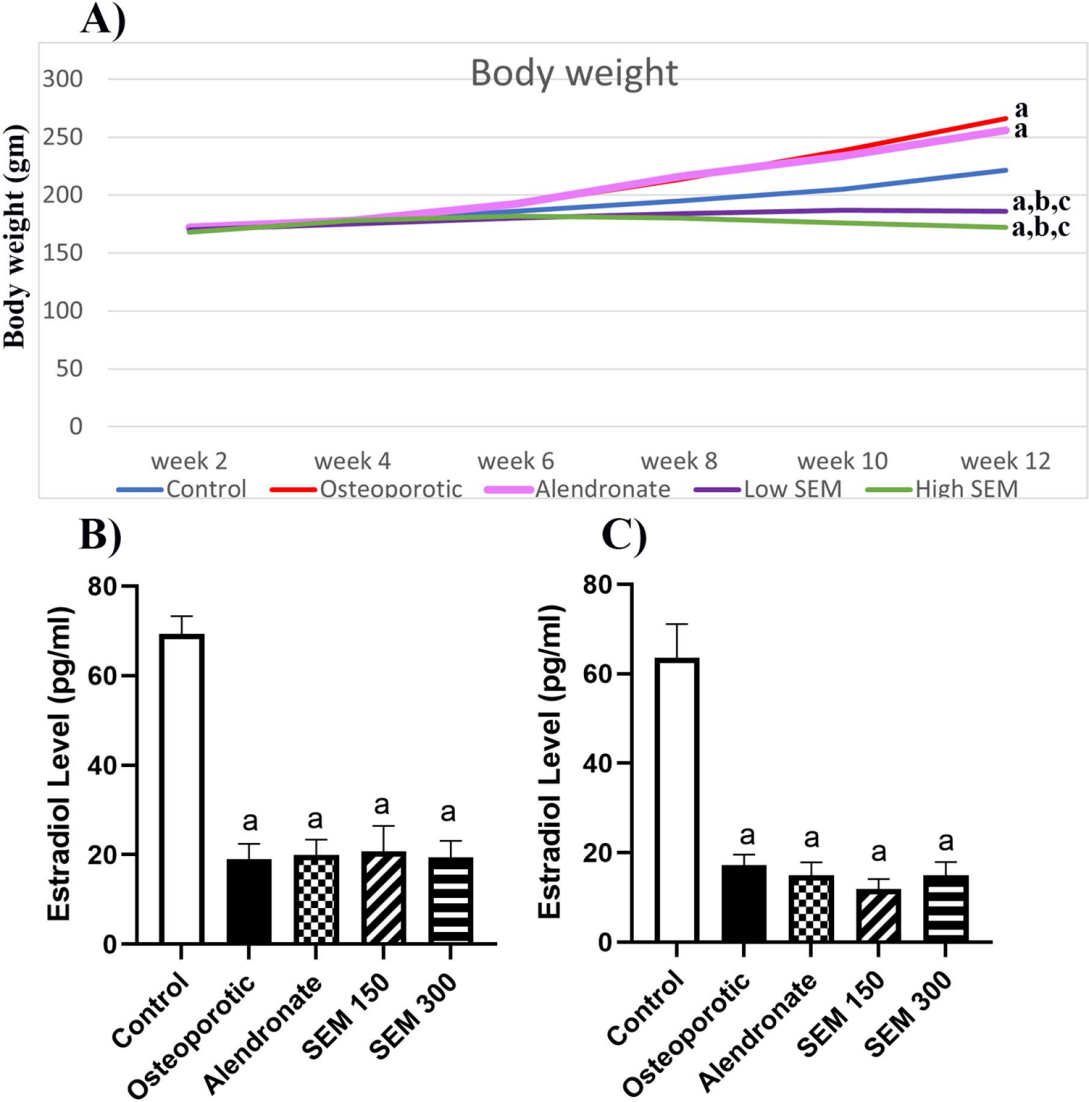
Effect of ovariectomy on **A** rat ‘s body weight and the serum estradiol level at **B** the 3rd week and **C** at the end of the experiment. Data is represented as mean ± SD (*n* = 6). a, significant from the control group; b, significant from the osteoporotic group; c, significant from the alendronate group. The test of significance was carried out using ANOVA followed by the Tukey–Kramer multiple comparison test

Estradiol levels in serum were measured two times. The first measurement was performed in the 3rd week of the experiment; rats in the osteoporotic group showed a significant decrease in estradiol levels by 72.64% when compared to the control group, and the other treated groups (alendronate group, semaglutide 150, and semaglutide 300) showed a significant decrease by 71.3%, 70.15%, and 72.1%, respectively, when compared to the control group. The second measurement was undertaken in the 12th week of the experiment; rats in the osteoporotic group showed a significant decrease in estradiol levels by 73% when compared to the control group, and the alendronate, semaglutide 150, and semaglutide 300 showed a decrease of 76.52%, 81.23%, and 76.4%, respectively, when compared to the control group. (Fig. 1).

### Effect of semaglutide on bone mineral density (BMD) in ovariectomized rats

**Fig. 2:**
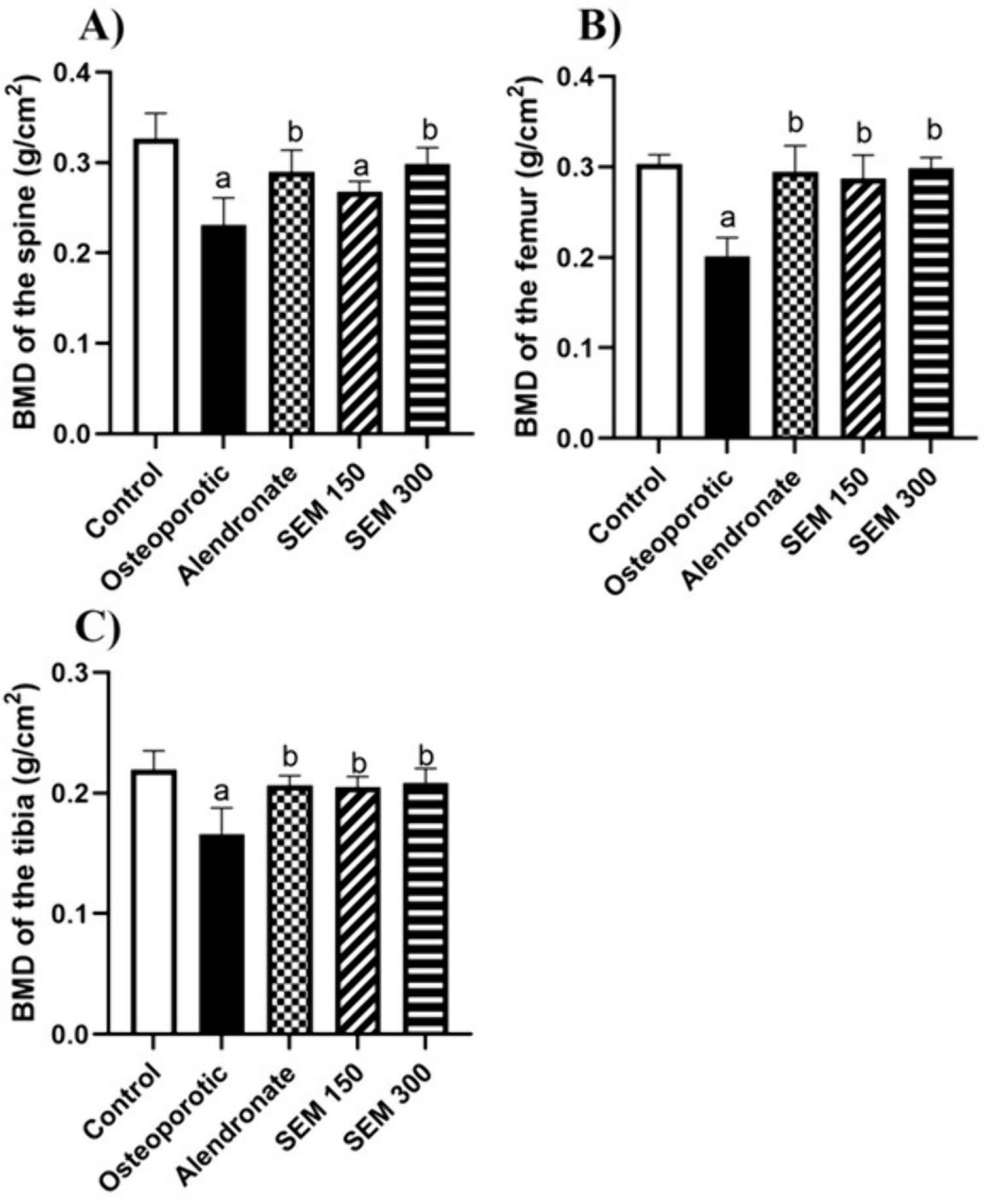
Effect of semaglutide on **A** BMD of the spine, **B** BMD of the femur, and **C** BMD of the tibia. Data is represented as mean ± SD. a, significant from the control group; b, significant from the osteoporotic group. The test of significance was carried out using ANOVA followed by the Tukey–Kramer multiple comparison test. BMD bone mineral density

The examination of bone performed by DEXA showed that ovariectomy reduced BMD in the spine, femur, and tibia by 29.2%, 33.5%, and 24.2%, respectively, when compared to the control group. Administration of semaglutide (150 mcg/kg) increased BMD by 15.65%, 42.39%, and 23.35% respectively when compared to the osteoporotic group, and administration of semaglutide (300 mcg/kg) showed an increase in BMD by 29.3%,48.7%, and 25.6%, respectively, when compared to the osteoporotic group. (Fig. 2).

### Effect of semaglutide on calcitonin, parathyroid hormone (PTH), vitamin D level, serum Ca^2+^, and serum phosphate level

Rats in the osteoporotic group showed a significant decrease in calcitonin levels compared to the control group by 49.2%, administration of semaglutide by doses of 150 and 300 mcg/kg showed a significant increase in calcitonin levels by 52.24% and 62.27%, respectively, when compared to the osteoporotic group.

Rats in the osteoporotic group showed a significant decrease in the PTH when compared to the control group by 55%. Rats treated with semaglutide 150 and 300 mcg/kg showed a significant increase in PTH when compared to the osteoporotic group by 92.3% and 98.1%, respectively.

Rats in the osteoporotic group showed a significant decrease in vitamin D levels compared to the control group by 61%. Administration of semaglutide in a dose of 150 mcg/kg showed an improvement in vitamin D levels in comparison to the osteoporotic group by 76.6%, which didn’t normalize the vitamin D level compared to the control group, while the higher dose (300 mcg/kg) normalized the vitamin D level compared to the control group and increased it by 1.61-fold compared to the osteoporotic group.

The Ca^2+^ serum levels in the osteoporotic group showed a significant increase compared to the control group by 21.1%. The treated groups’ alendronate and semaglutide 150 and 300 showed a significant decrease in Ca^2+^ compared to the osteoporotic group by 16.6%, 16.7%, and 16.7%, respectively. The treated groups normalized the levels of Ca^2+^ compared to the control group.

The phosphate serum level in the osteoporotic groups showed a significant increase compared to the control group by 18.75%. The treated groups’ alendronate and semaglutide 150 and 300 showed a significant decrease in phosphate compared to the osteoporotic by 10.5% for all the treated groups. There was no significant difference between the treated groups and the control group (Table 1).

**Table 1:** Effect of semaglutide on A serum calcitonin level; B serum PTH level; C serum vitamin D level; D serum Ca^2+^level; and E serum phosphate level

Parameter	Control group	Osteoporotic group	Alendronate group	SEM 150 group	SEM 300 group
A. Calcitonin (pg/ml)	**82.8 ± 2.53**	**42.1**^**a**^** ± 4.44**	**69.0**^**a,b**^** ± 5.71**	**64.1**^**a,b**^** ± 2.68**	**68.3**^**a,b**^** ± 5.31**
B. PTH (pg/ml)	**234.1 ± 17.13**	**105.4**^**a**^** ± 6.31**	**194.2**^**a,b**^** ± 8.31**	**202.7**^**a,b**^** ± 8.44**	**208.8**^**a,b**^** ± 15.63**
C. Vitamin D (ng/ml)	**36.92 ± 5.81**	**14.37**^**a**^** ± 1.03**	**26.0**^**a,b**^** ± 2.08**	**25.38**^**a,b**^** ± 4.06**	**37.60**^**b,c,d**^** ± 3.54**
D. Serum Ca^2+^ level (mg/dl)	**9.9 ± 0.50**	**12.0**^**a**^** ± 0.50**	**10.0**^**b**^** ± 0.35**	**10.0**^**b**^** ± 0.48**	**10.0**^**b**^** ± 0.72**
E. Serum phosphate level (mg/dl)	**4.8 ± 0.38**	**5.7**^**a**^** ± 0.15**	**5.1**^**b**^** ± 0.08**	**5.1**^**b**^** ± 0.13**	**5.1**^**b**^** ± 0.29**

Data is represented as mean ± SD. Test of significance was carried out using ANOVA followed by the Tukey–Kramer multiple comparison test

*PTH* parathyroid hormone

^a^Significant from the control group

^b^Significant from the osteoporotic group

^c^Significant from the alendronate group

^d^Significant from the SEM 150 group

### Effect of semaglutide on bone formation, resorption markers, and interleukin 6 (IL-6)

Bone-specific alkaline phosphatase (BALP) is a sensitive and reliable marker of bone formation activity. The BALP serum level in the osteoporotic group showed a significant increase compared to the control group by 2.85-fold. The rats treated with semaglutide (150 and 300 mcg/kg) showed a significant decrease in BALP by 55.6% and 64.96%, respectively, compared to the osteoporotic group.

Osteocalcin is hypothesized to act in extracellular matrix mineralization and is used as a serum marker of osteoblastic bone formation. The osteocalcin serum level in the osteoporotic group showed a significant increase compared to the control group by 4.21-fold. The rats treated with semaglutide (150 mcg/kg) showed a decrease in the osteocalcin level by 67.48% compared to the osteoporotic group, while the rats treated with semaglutide (300 mcg/kg) showed a decrease in the osteocalcin level by 74.63% compared to the osteoporotic group.

RANKL is a member of the tumor necrosis factor (TNF) family and is recognized as the only cytokine that performs a critical role in bone metabolism by regulating osteoclast formation, maintenance, and activation and is responsible for bone resorption (Weitzmann 2013). Rats in the osteoporotic group showed a significant increase in the receptor activator of nuclear factor kappa-Β ligand (RANKL) level compared to the control group by 2.5-fold. Administration of semaglutide in doses 150 and 300 mcg/kg significantly decreased the RANKL level compared to the osteoporotic group by 63.77% and 68.45%, respectively. The treated group with semaglutide showed a significant difference when compared to the alendronate group.

Type I collagen is the most prevalent protein component in bone, and C-terminal telopeptides of type I collagen carboxy-terminal collagen crosslinks (CTX) are type I collagen fragments from the telopeptide region. The telopeptides are cleaved during osteoclastic resorption of bone, allowing them to enter the bloodstream at a rate proportionate to bone resorption activity (Greenblatt et al. 2017).

Rats in the osteoporotic group showed a significant increase in the CTX level compared to the control group by twofold. Rats treated with 150 and 300 mcg/kg of semaglutide showed a significant decrease in CTX level compared to the osteoporotic group by 46.1% and 54.76%, respectively.

The decrease in CTX level for rats treated with semaglutide 300 mcg/kg was better than the alendronate group.

Pyridinoline (PYD) collagen crosslinks are formed during the extracellular maturation of fibrillar collagens and released into the bloodstream as mature collagens degrade and are considered bone resorption markers (Kuo & Chen 2017).

Rats in the osteoporotic group showed a significant increase in PYD levels compared to the control group by 2.58-fold. Administration of semaglutide by doses of 150 and 300 mcg/kg showed an osteoprotective effect by a decrease in PYD level by 39.1% and 59.4% compared to the osteoporotic group.

**Fig. 3:**
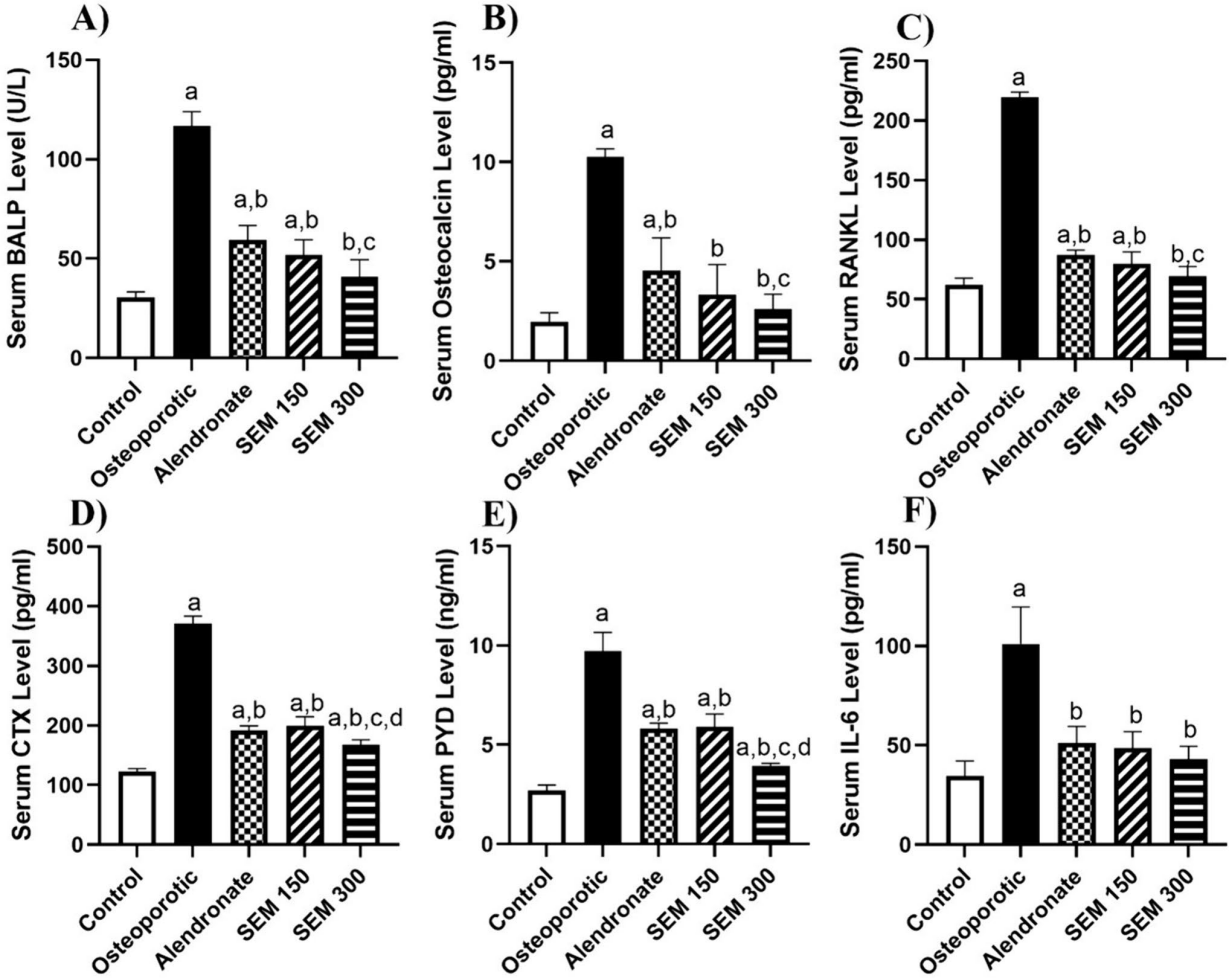
Effect of semaglutide on **A** serum BALP level, **B** serum OCN level, **C** serum RANKL level, **D** serum CTX level, **E** serum PYD level, and **F** serum IL-6 level. Data is represented as mean ± SD. a, significant from the control group; b, significant from the osteoporotic group; c, significant from the alendronate group; d, significant from the SEM 150 group. The test of significance was carried out using ANOVA followed by the Tukey–Kramer multiple comparison test. BALP bone-specific alkaline phosphatase, CTX carboxy-terminal collagen crosslinks, IL-6 interleukin 6, OCN osteocalcin, PYD pyridinoline, RANKL receptor activator of nuclear factor kappa-Β ligand

Inflammatory cytokines such as IL-6 can regulate skeletal balance and the differentiation of osteoclasts. The binding of IL-6 to receptors on precursor osteoclasts stimulates osteoclast formation, leading to elevated bone resorption (Harmer et al. 2019). Rats in the osteoporotic group showed a significant increase in the serum IL-6 by 1.92-fold compared to the control group. Rats treated with 150 and 300 mcg/kg of semaglutide showed a significant decrease in the serum IL-6 by 52% and 57.4%, respectively, when compared to the osteoporotic group. The two doses of semaglutide did not differ significantly from the alendronate group and the control group (Fig. 3).

### Effect of semaglutide on GLP-1R

**Fig. 4:**
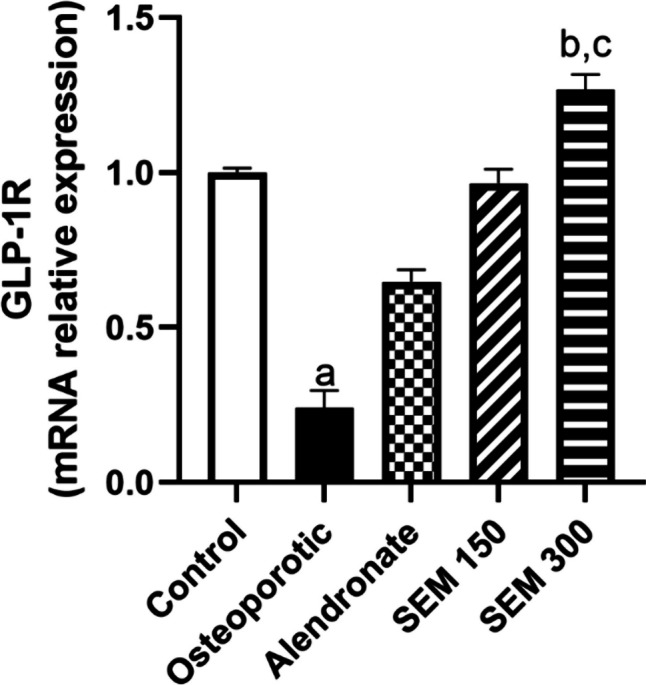
Effect of semaglutide on GLP-1R mRNA relative expression. Data is represented as mean ± SD. a, significant from the control group; b, significant from the osteoporotic group; c, significant from the alendronate group. The test of significance was carried out using the non-parametric Kruskal–Wallis test. GLP-1R glucagon-like peptide-1 receptor

Rats in the osteoporotic group showed a significant decrease in the expression of GLP-1R by 75.83% compared to the control group. In contrast, administration of semaglutide in doses of 150 and 300 mcg/kg attenuated this downregulation of GLP-1R mRNA expression and showed an increase in GLP-1R expression by 2.99-fold and 4.24-fold, respectively, compared to the osteoporotic group as seen in Fig. 4.

### Effect of semaglutide on cAMP

**Fig. 5:**
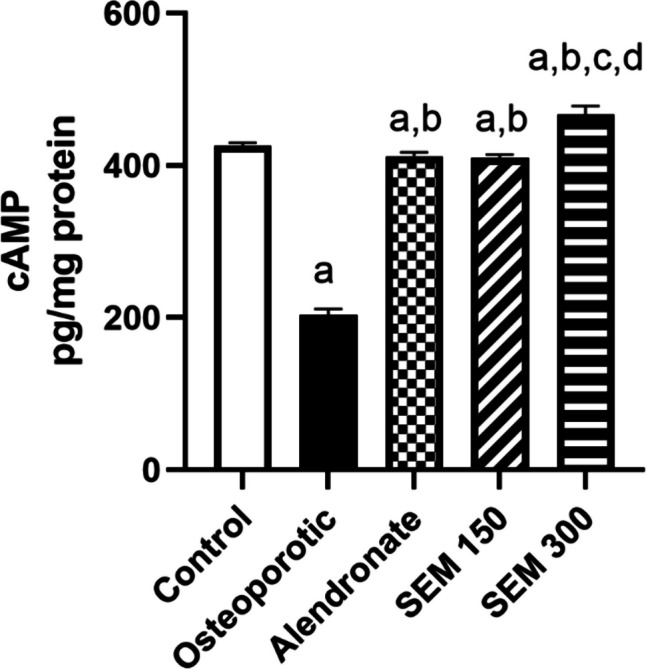
Effect of semaglutide on cAMP. Data is represented as mean ± SD. a, significant from the control group; b, significant from the osteoporotic group; c, significant from the alendronate group; d, significant from the SEM 150 group. The test of significance was carried out using ANOVA followed by the Tukey–Kramer multiple comparison test. cAMP cyclic adenosine monophosphate

Rats in the osteoporotic group showed a significant decrease in cAMP levels compared to the control group by 52.38%. Administration of semaglutide by doses of 150 and 300 mcg/kg showed an increase in cAMP by 1.02-fold and 1.3-fold, respectively, compared to the osteoporotic group (Fig. 5).

### Effect of semaglutide on renal function

Renal function tests were conducted to rule out the possibility of renal failure causing bone resorption.

**Fig. 6:**
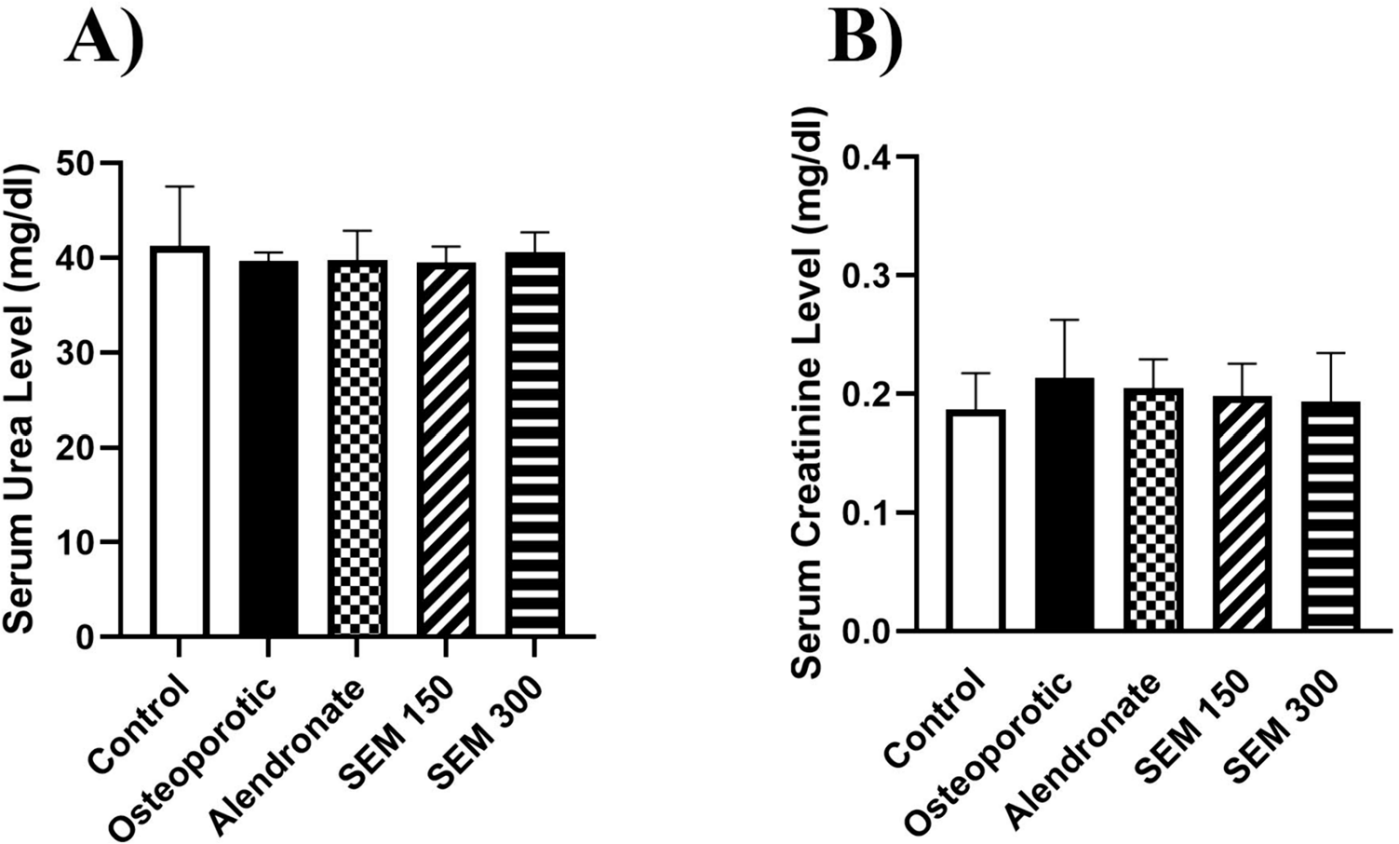
Kidney function tests. Data is represented as mean ± SD. The test of significance was carried out using ANOVA followed by the Tukey–Kramer multiple comparison test. **A** serum urea level; **B** serum creatinine level

Rats in the osteoporotic group showed a non-significant difference in creatinine and urea levels when compared to the control, alendronate, semaglutide 150, and semaglutide 300 mcg/kg (Fig. 6).

### Effect of semaglutide on bone histomorphometry

Rats in the osteoporotic group showed a significant decrease in the trabecular bone area percentage by 59.3% compared to the control group. Rats treated with 150 and 300 mcg/kg showed an osteoprotective effect by a decrease in trabecular bone percentage by 81.1% and 154.4%, respectively, compared to the osteoporotic group.

Rats in the osteoporotic group showed a significant decrease in the trabecular width by 31.48% compared to the control group. Rats treated with semaglutide 150, and 300 mcg/kg showed a significant increase in the trabecular width by 47.94% and 15.64%, respectively, compared to the osteoporotic group.

**Fig. 7:**
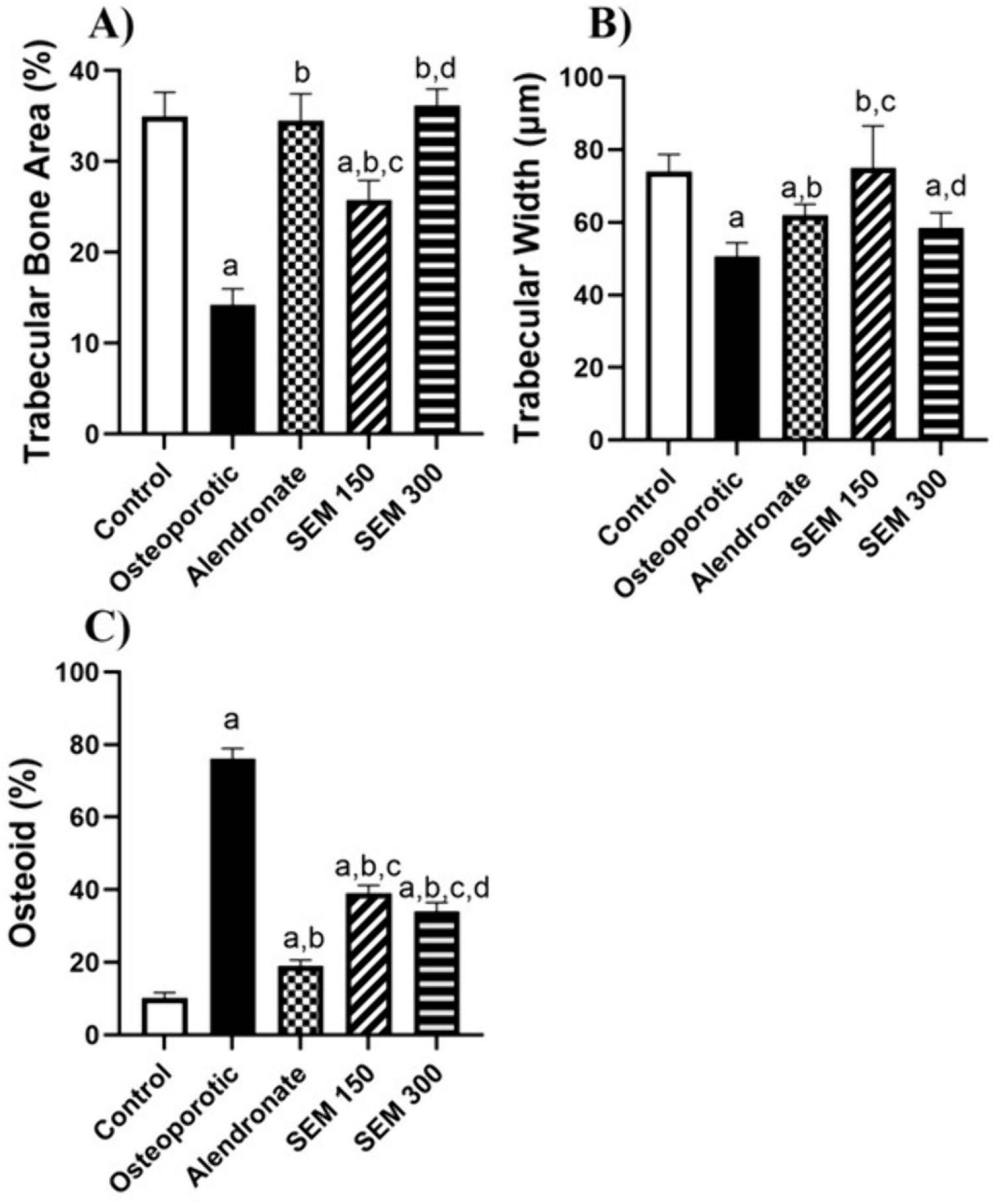
Effect of semaglutide on **A** the trabecular bone area, **B** trabecular width, and **C** osteoid percent. Data is represented as mean ± SD. a, significant from the control group; b, significant from the osteoporotic group; c, significant from the alendronate group; d, significant from the SEM 150 group. The test of significance was carried out using ANOVA followed by the Tukey–Kramer multiple comparison test

Rats in the osteoporotic group showed a significant increase in the osteoid percent by 6.5 folds compared to the control group. Rats treated with semaglutide 150 and 300 mcg/kg showed a significant decrease in the osteoid percent by 48.6% and 55.3%, respectively, compared to the osteoporotic group (Fig. 7).

### Effect of semaglutide on bone histopathological examination

**Fig. 8:**
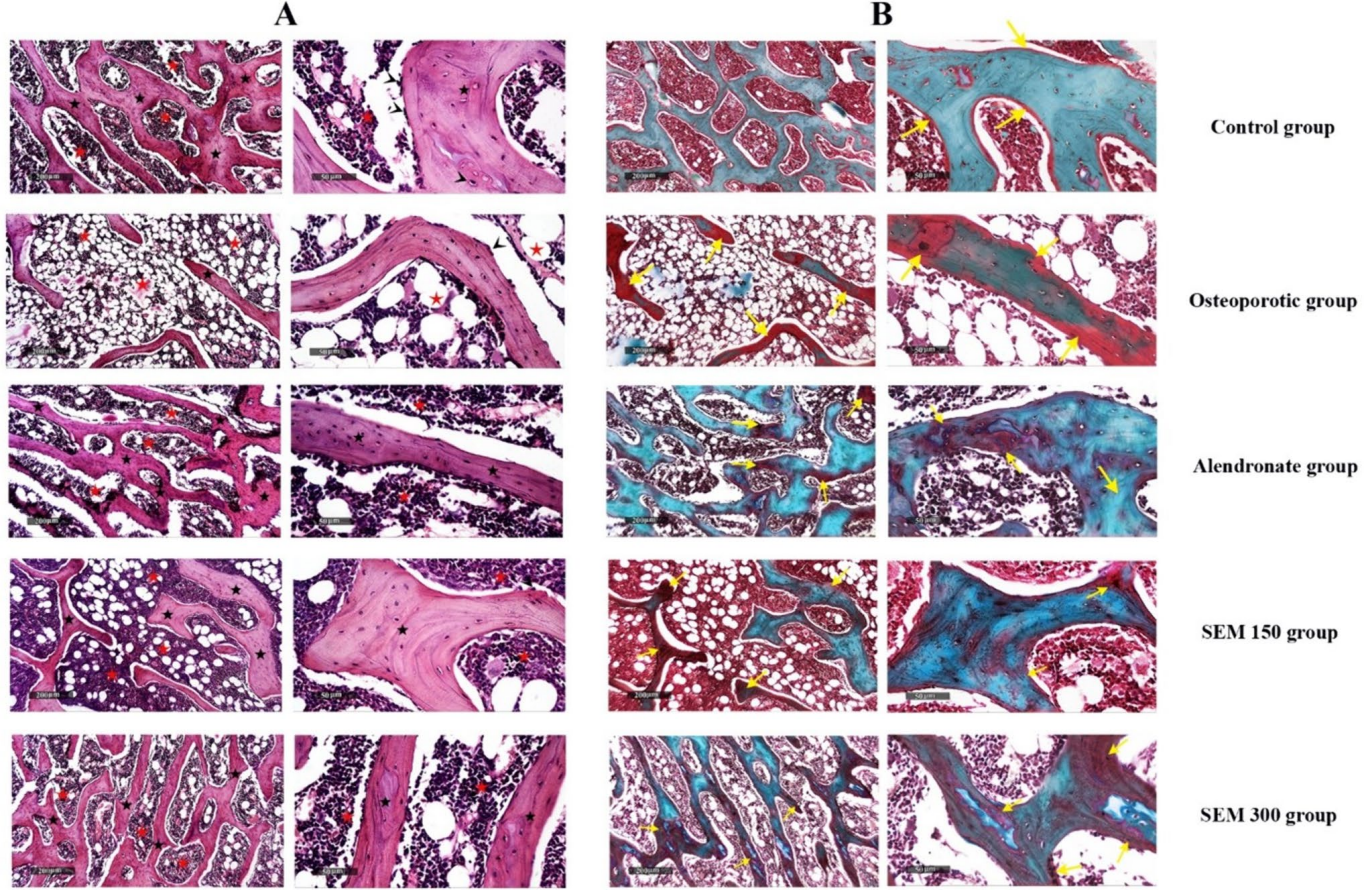
**A** H&E Histopathological changes of the bone matrix. In the control group, the black star demonstrated normally organized histological architectures of bony trabeculae, the arrowhead indicates normally distributed osteoblasts and osteocytes, and the red star indicates normal widening of intertrabecular spaces. In the osteoporotic group, black stars show deformity and disorganized morphological features of trabecular bone, arrowhead indicates a significant loss of osteoblastic activity, and red stars show an increase of intertrabecular marrow spaces accompanied by remarkable hypoplasia of bone marrow. In the alendronate group, the black stars demonstrated significant improvement in morphological features of bony samples, the arrowhead showed higher osteoblastic activity, and the red stars showed a normal organization of bone marrow cellular elements. In the SEM 150 group, the black stars showed moderate protective efficacy with persistent records of moderate loss of trabecular bone area, the arrowhead showed an increase in osteoblastic activity, and the red stars indicated a significant restoration of bone marrow cellular elements. **B** The Goldner Trichrome stain for bone tissue. In the control group, the yellow arrow indicates an intact intercellular bony matrix with minimal cracks or porosity records. In the osteoporotic group, the yellow arrow indicates the focal records of trabecular cracks and fissures. In the alendronate group, the yellow arrow indicates marked improvement in non-mineralized bone matrix area percentage. In the SEM 150 group, the yellow arrow indicates a marked improvement in non-mineralized bone matrix area percentage. In the SEM 300 group, the yellow arrow indicates up to 55% decreased non-mineralized bone matrix area percentage compared to the osteoporotic group

The control group demonstrated normally organized histological architectures of bony trabeculae as shown in Fig. 7 with normally distributed osteoblasts and osteocytes with minimal cracks or porosity records with normal widening of intertrabecular spaces without abnormal alteration of bone marrow cellularity. In the osteoporotic group, marked deformity and disorganized morphological features of trabecular bone. An obvious decrease of up to 59% of relative total trabecular density was recorded compared to normal control samples with a significant decrease of the trabecular thickness, and a significant loss of osteoblastic activity was shown with focal records of trabecular cracks and fissures with a marked increase of intertrabecular marrow spaces accompanied by remarkable hypoplasia of bone marrow with significant vacuolization recorded. The alendronate group demonstrated significant improvement in morphological features of bony samples with obvious records of apparent intact trabecular bone with up to a 2.4-fold increase of relative trabecular bony area. Significantly higher osteoblastic activity was observed with the normal organization of bone marrow cellular elements. The SEM 150 group showed moderate protective efficacy with persistent records of moderate loss of trabecular bone area up to a 1.7-fold increase compared to the osteoporotic group, almost normalized trabecular bone thickness without abnormal deformities, with a moderate increase of osteoblastic activity with the significant restoration of bone marrow cellular elements. The SEM 300 group demonstrated evidence of higher protective efficacy compared to the lower dose with almost the same morphology as alendronate group samples (Fig. 8).

### Effect of semaglutide on PKA, AKT, p-AKT, p-β-Catenin, p-GSK3β, and BMP-2 expression:

In the current study, Western blot analysis was employed to estimate canonical Wnt signaling pathway activation and to investigate the mechanistic pathway of the osteoprotective effect of semaglutide. The current result showed that PKA, p-AKT, and p-β-Catenin _(Ser675)_ expression increased significantly by 2.65-fold, 2.63-fold, and 3.28-fold, respectively, upon administration of semaglutide (300 mcg/kg) versus the osteoporotic group.

The osteoporotic group showed an increase in GSK3 β expression by 2.4-fold versus the control group. While, in comparison to the osteoporotic group, treatment with semaglutide (300 mcg/kg) significantly decreases the expression of GSK3 β by 82.31%.

**Fig. 9:**
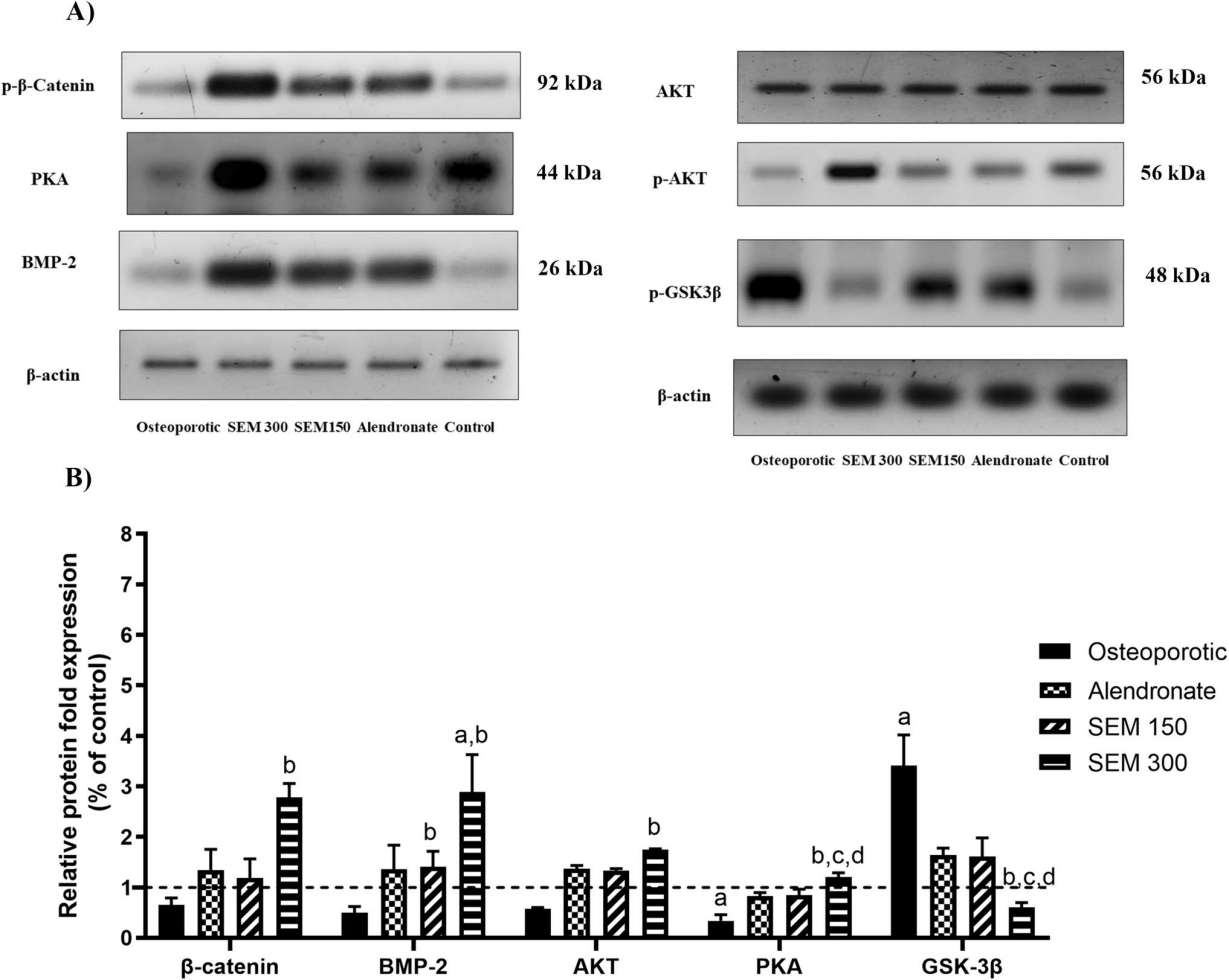
Effect of semaglutide on **A** western blots for p-β-catenin, AKT, p-AKT, BMP-2, β-actin, p-GSK-3β, and PKA and **B** the relative protein expression relative to control. Data is represented as mean ± SD. a, significant from the control group; b, significant from the osteoporotic group; c, significant from the alendronate group; d, significant from the SEM 150 group. The test of significance was carried out using the non-parametric Kruskal–Wallis test. The experiment was performed in triplicate. AKT protein kinase B, BMP-2 bone morphogenetic protein-2, GSK-3β glycogen synthase kinase-3 beta, PKA protein kinase A

The semaglutide 150 and 300 mcg/kg showed a significant increase in BMP-2 expression by 1.84-fold and 4.82-fold, respectively, in comparison to the osteoporotic group (Fig. 9).

## Discussion

In our study, we investigated the effect of semaglutide on ovariectomy-affected bone turnover and bone loss in Sprague–Dawley rats. Our results were not different from those of Babaei et al. (2010) and Ström et al. (2012) who showed that alendronate and semaglutide do not affect the estradiol level. Semaglutide causes a significant decrease in body weight compared to the osteoporotic rats which were obese. Semaglutide decreases body weight via direct effects on the hypothalamus and the hindbrain; the semaglutide might influence body weight through indirect neural signals to important relay centers that control appetite (Gabery et al. 2020). As well as indirect effects on several energy-metabolizing regions (Roh & Choi 2023). Blundell et al. (2017) have shown that semaglutide activates the GLP-1 in the hypothalamus which affects energy balance.

Here, ovariectomy-induced bone loss was validated by a significant decrease in femoral, tibial, and lower spine BMD in the osteoporotic group compared to the other group. These results are similar to those of Bonnet et al. (2008) and Yousefzadeh et al. (2020b). This demonstrated a direct relationship between low estrogen levels and BMD. Compared to the other groups, the changes in BMD in an osteoporotic group ensured the presence of osteoporosis.

Bone loss was also confirmed in this study by the significant rise in serum Ca^2+^ levels and serum phosphate levels. These results were consistent with those of Yousefzadeh et al. (2020a, b). This might be explained by the activation of osteoclasts, which results in the extracellular fluid being infused with Ca^2+^ and phosphate, thus reducing the production of vitamin D (Tebben et al. 2016). The decrease in serum vitamin D leads to a decrease in intestinal Ca^2+^ and phosphate absorption (de Brito Galvao et al. 2013). In osteoporotic rats, estrogen deprivation leads to osteoclast activation so the serum level of Ca^2+^ increases. The semaglutide groups and alendronate group showed a normalization for Ca2 + , phosphate, vitamin D, and PTH since these parameters form a specific cascade. The fluctuations in serum Ca^2+^ are detected by the Ca^2+^-sensing receptor (CaSR), a glycoprotein that is a G-protein-coupled receptor on the cell surface of parathyroid gland chief cells (Riccardi & Brown 2010; Hebert et al. 1997). That results in a decreased PTH level, which is consistent with what was shown by Rader et al. (1979) and Smajilovic et al. (2010). PTH has multiple essential roles in Ca^2+^ homeostasis that is, encouraging bone resorption (Silva & Bilezikian 2015) and Ca^2+^ reabsorption by the kidneys (Moor & Bonny 2016). However, the influence of PTH on the uptake of Ca^2+^ is subtle and depends on PTH’s ability to regulate renal vitamin D metabolism (Armbrecht et al. 2003; Zierold et al. 2003). Since estradiol levels were all the same in the osteoporotic, alendronate, and semaglutide groups, and the normalization of Ca^2+^ level only occurred in alendronate and semaglutide, then it could only be concluded that semaglutide osteoprotective effects are separate from estradiol level.

Three key phases may be used to illustrate the osteogenesis process: proliferation, matrix maturation, and mineralization. Bone-specific alkaline phosphatase (BALP), a major enzyme indicating extracellular matrix maturation in the process of bone formation, is one of the most often utilized indicators of the osteoblast differentiation process (Salhotra et al. 2020). By converting the phosphoric ester into inorganic phosphorus, BALP can increase the mineralization of the bone matrix and raise phosphorus levels (Ren et al. 2020).

In this study, the serum osteocalcin (OCN) and BALP were markedly elevated, which may contribute to the inhibition of Ca^2+^ mineralization and the osteoclast breakdown of the bone matrix (Ivaska et al. 2004).

The weekly treatment of semaglutide in osteoporotic female rats enhanced BMD and demonstrated a significant reduction in serum OCN and BALP. The process might be attributed to the activation of osteoblasts (Henry & Bordoni 2022), which increased bone formation indicators such as BALP and OCN, resulting in Ca^2+^ mineralization in the bone matrix.

In addition, histological examination demonstrated that semaglutide reduced the altered bone structure when compared to the osteoporotic group. Semaglutide showed a direct influence on bone formation indicators in bone marrow stromal cells, suggesting that the predominant effect was on osteoblasts.

The elevated blood levels of CTX and PYD in osteoporotic rats revealed accelerated bone resorption via osteoclastic cells in the current study, which is similar to what was shown by Kim et al. (2011) and Yoon et al. (2012). In our experiment, a significant increase in serum RANKL suggested osteoclastogenesis. RANKL works to stimulate the differentiation, activation, and survival of osteoclasts by binding to the receptor activator of nuclear factor kB (RANK) on the cell surface of osteoclasts or their progenitors (Mai et al. 2011). Reduced blood RANKL levels imply that osteoclast development is inhibited, which therefore inhibits osteoclastogenesis and prevents cancellous bone loss in ovariectomized rats (Bae & Kim 2010; Mai et al. 2011). When compared to control bone marrow stromal cells (BMSCs), ovariectomized BMSCs had a higher adipogenic differentiation potential (Ren et al. 2020). These findings may assist in explaining clinical observations of postmenopausal osteoporotic women’s increased fat and reduction in trabecular bone volume in the marrow cavity. The Wnt/ß-catenin pathway is vital for osteogenic differentiation and bone formation (Grigorie & Lerner 2018). Previous studies have shown that the Wnt canonical pathway is critical in the differentiation of BMSCs (Houschyar et al. 2019; Wu et al. 2019).

**Fig. 10:**
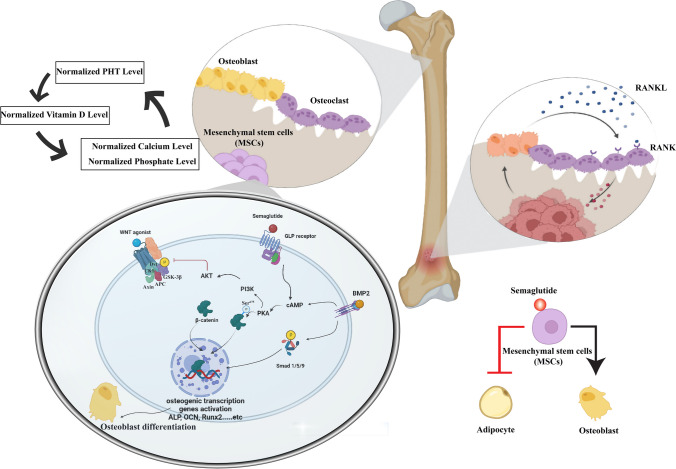
Diagram for the suggested mechanism of drug action. A suggested model illustrating the role of GLP-1R in promoting BMSCs osteoblast differentiation and suppressing their differentiation into adipocytes. Semaglutide binds to GLP-1R and activates adenylyl cyclase, resulting in the downstream formation of cAMP and the activation of PKA. That leads to the phosphorylation of beta-catenin on Ser675, which inhibits the downstream degradation of beta-catenin and increases its quantity in the cytosol of the pre-osteoblasts. In addition to phosphorylating PI3K, the activated PKA also activates AKT. PKA that has been phosphorylated subsequently phosphorylates GSK3b, inhibiting GSK3b activity. Thus, GSK3b-mediated beta-catenin degradation is inhibited, and nuclear accumulation of b-catenin is enhanced. Which leads to an increase in the transcription and initiation of osteogenic gene activation and osteoblast differentiation. That leads to anabolic bone formation. In addition, Bone formation and osteoblast differentiation are stimulated by BMP-2. Which augments semaglutide signaling by increasing the cAMP and increasing the expression of the osteogenic genes. Proper balancing between osteoblast and osteoclast leads to the normalization of PTH, vitamin D, Ca^2+^, and phosphate levels

In our investigation, we explain the association between semaglutide, GLP-1R, and the Wnt/β-catenin signaling pathways. The GLP-1R receptor interacts with the canonical Wnt/β-catenin pathway to enhance the formation of bone. The findings of this study showed that GLP-1R, mostly expressed in BMSCs, enhances the process of osteoblast development (Habib et al. 2021). The GLP-1R receptor synergistically interacts with the canonical Wnt/β-catenin pathway to promote the process of bone formation. The main mechanisms by which GLP-1R mediates osteogenic action involve the cAMP/PKA/β-catenin pathway, which initiates osteoblast differentiation. Additionally, GLP-1R acts via the PKA/Akt/GSK3β pathway by preventing β-catenin degradation and promoting its accumulation in the nucleus of BMSCs. These processes ultimately lead to the formation of new bone tissue (Liu et al. 2013c; Luo et al. 2016). Our findings show that semaglutide increases the β-catenin by increasing the cAMP which activates the protein kinase A (PKA) leading to the modification of β-catenin enzymatically by adding phosphate groups to its Ser675 residue, which facilitates the accumulation and translocation of β-catenin into the nucleus. This event then triggers the activation of genes associated with the differentiation of BMSCs into osteoblasts, leading to their transformation into bone-forming cells. PKA has been shown to phosphorylate β-catenin at two specific locations, Ser552 and Ser675 (Hino et al. 2005; Taurin et al. 2006). This phosphorylation process prevents the ubiquitination of β-catenin, leading to its stabilization. Furthermore, PKA phosphorylates phosphatidylinositol-3-kinase (PI3K), subsequently resulting in the phosphorylation of protein kinase B (AKT), ultimately leading to the phosphorylation of Glycogen synthase kinase-3β (GSK-3β). GSK3β is an essential enzyme that exerts an inhibitory effect on the canonical Wnt/β-catenin signaling pathway. Furthermore, the triggering of canonical Wnt signaling requires the suppression of GSK3β activity (Clevers & Nusse 2012; Gillespie et al. 2013). In our findings, Akt was increased and GSK3β was decreased after treatment with semaglutide, further promoting the role of Wnt/β-catenin in bone formation as illustrated in Fig. 10.

BMP-2 is critical for BMSC differentiation and osteogenesis. BMSCs can develop into osteoblasts when new bone has to be generated. These osteoblasts can become osteocytes, which give extra bone support and structure. BMP-2, released into the bone matrix during osteoclast-driven bone resorption, is the primary agent differentiating BMSCs into osteoblasts. Thus, increased expression of BMP-2 prevents loss of BMD from the bone and protects the bone from ovarian deficiency-induced osteoporosis (Halloran et al. 2020). Various types of cells, including osteoclasts, osteoblasts, and osteocytes, maintain the bone, and intercellular communication between these cells is facilitated by hormones and cytokines, the nervous system, and cell-to-cell adhesion. Inducing improper differentiation and function of bone cells, abnormal cytokines contribute to the development of bone disease. IL-6 may induce RANKL via stimulation of several cells and enhance osteoclast differentiation (Takeuchi et al. 2021). Our findings show that the elevated expression of BMP-2 increased bone formation through interaction with cAMP and decreased bone resorption through RANKL inhibition in semaglutide-treated groups as shown in Fig. 9.

Increased expression of β-catenin improves BMD by inducing the BMSC to differentiate into osteoblasts that maintain the bone microarchitecture, enhance its density, and restore the normal balance that the estrogen deficiency disrupted (Chen & Long 2013).

The use of targeted delivery of anti-osteoporotic drugs for semaglutide may limit or prevent drug distribution toward non-bone tissues and early metabolism, as well as diminish the negative effects induced by excessive systemic drug exposure (Shi et al. 2020). Targeted ligands having a specific affinity for bone-associated cells, or the bone matrix show potential for increasing semaglutide accumulation in the bone (Guo et al. 2021; Khajuria et al. 2017). So, bisphosphonates such as alendronate exhibit promising bone-targeting potential (Farrell et al. 2018). The assumption of using semaglutide/alendronate nanoparticles may have the potential to increase the efficacy of bone formation and decrease bone resorption; however, further studies are mandatory to prove the efficacy and to study the pharmacodynamic effects (Ma et al. 2023).

## Conclusion

Our study revealed that semaglutide demonstrated remarkable prophylactic effects as an anti-osteoporotic in ovariectomy-induced osteoporosis, which may be linked to an increase in osteoblast formation caused by the activation of Wnt signaling. Furthermore, semaglutide prevented bone resorption and decreased the expression of RANKL on osteoblastic cells. This decrease in RANKL serves to prevent bone deterioration. We recommend the consideration of semaglutide in such situations where obesity is accompanied by osteoporosis.

## Supplementary Information

Below is the link to the electronic supplementary material.

Supplementary file1 (JPG 455 KB)

Supplementary file2 (JPG 331 KB)

Supplementary file3 (JPG 405 KB)

Supplementary file4 (JPG 291 KB)

Supplementary file5 (JPG 237 KB)

Supplementary file6 (JPG 489 KB)

Supplementary file7 (JPG 259 KB)

Supplementary file8 (JPG 404 KB)

Supplementary file9 (JPG 289 KB)

Supplementary file10 (JPG 475 KB)

Supplementary file11 (JPG 246 KB)

Supplementary file12 (JPG 550 KB)

Supplementary file13 (JPG 278 KB)

Supplementary file14 (JPG 552 KB)

Supplementary file15 (JPG 319 KB)

Supplementary file16 (JPG 479 KB)

Supplementary file17 (JPG 310 KB)

Supplementary file18 (JPG 542 KB)

Supplementary file19 (PDF 1568 KB)

## Data Availability

The authors declare that the data supporting the findings of this study are available within the paper and its supplementary information files. Should any raw data files be needed in another format they are available from the corresponding author upon reasonable request.
