# Supplementary figures and images for: The crucial role of beta-catenin in the osteoprotective effect of semaglutide in an ovariectomized rat model of osteoporosis
Source: Naunyn Schmiedebergs Arch Pharmacol. 2024 Sep 10;398(3):2677–93. doi: 10.1007/s00210-024-03378-z (PMC11920005; doi:10.1007/s00210-024-03378-z)

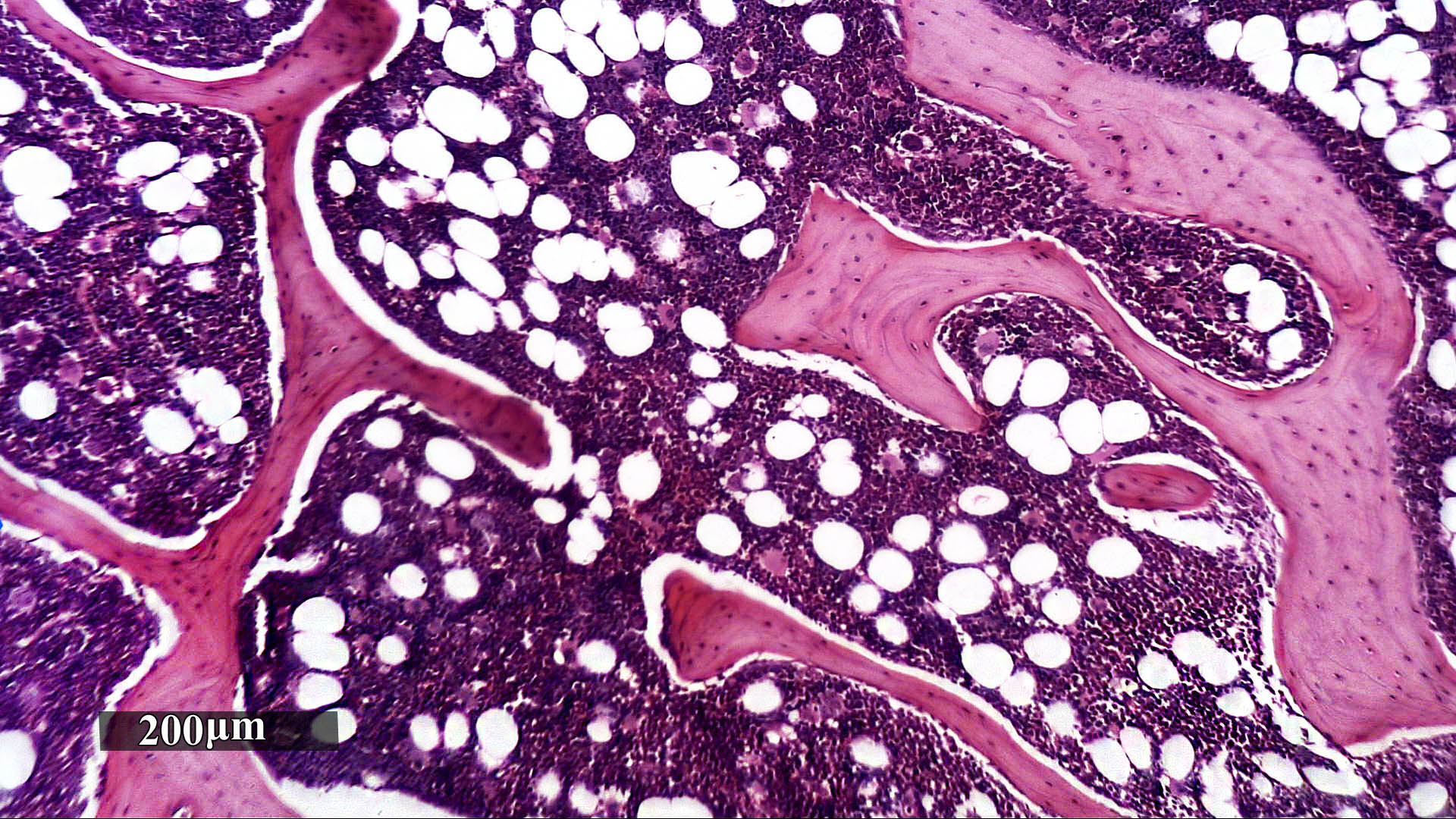

Supplement: Supplementary file 1 — Supplementary file1 (JPG 455 KB) [file 210_2024_3378_MOESM1_ESM.jpg]

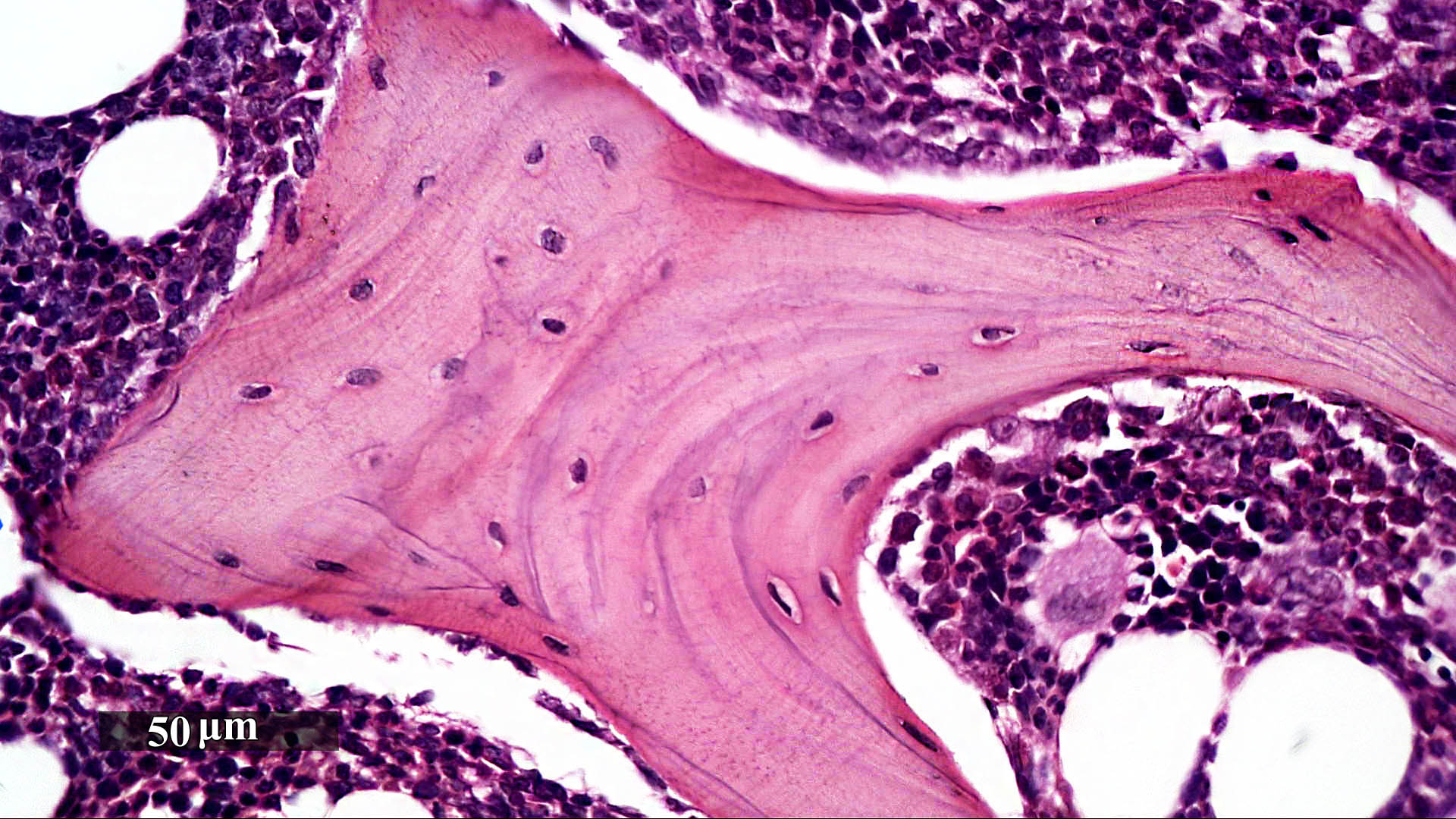

Supplement: Supplementary file 2 — Supplementary file2 (JPG 331 KB) [file 210_2024_3378_MOESM2_ESM.jpg]

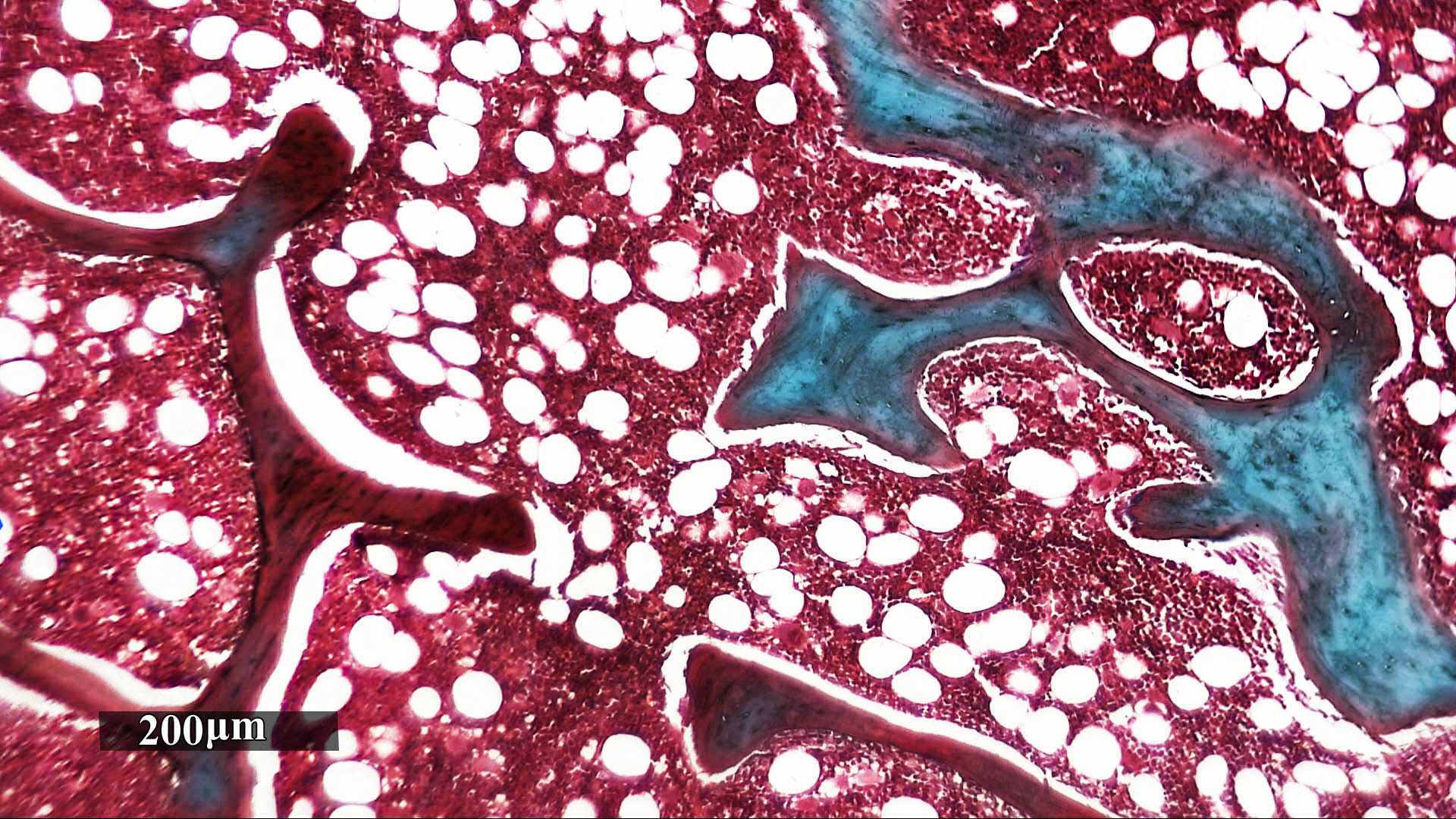

Supplement: Supplementary file 3 — Supplementary file3 (JPG 405 KB) [file 210_2024_3378_MOESM3_ESM.jpg]

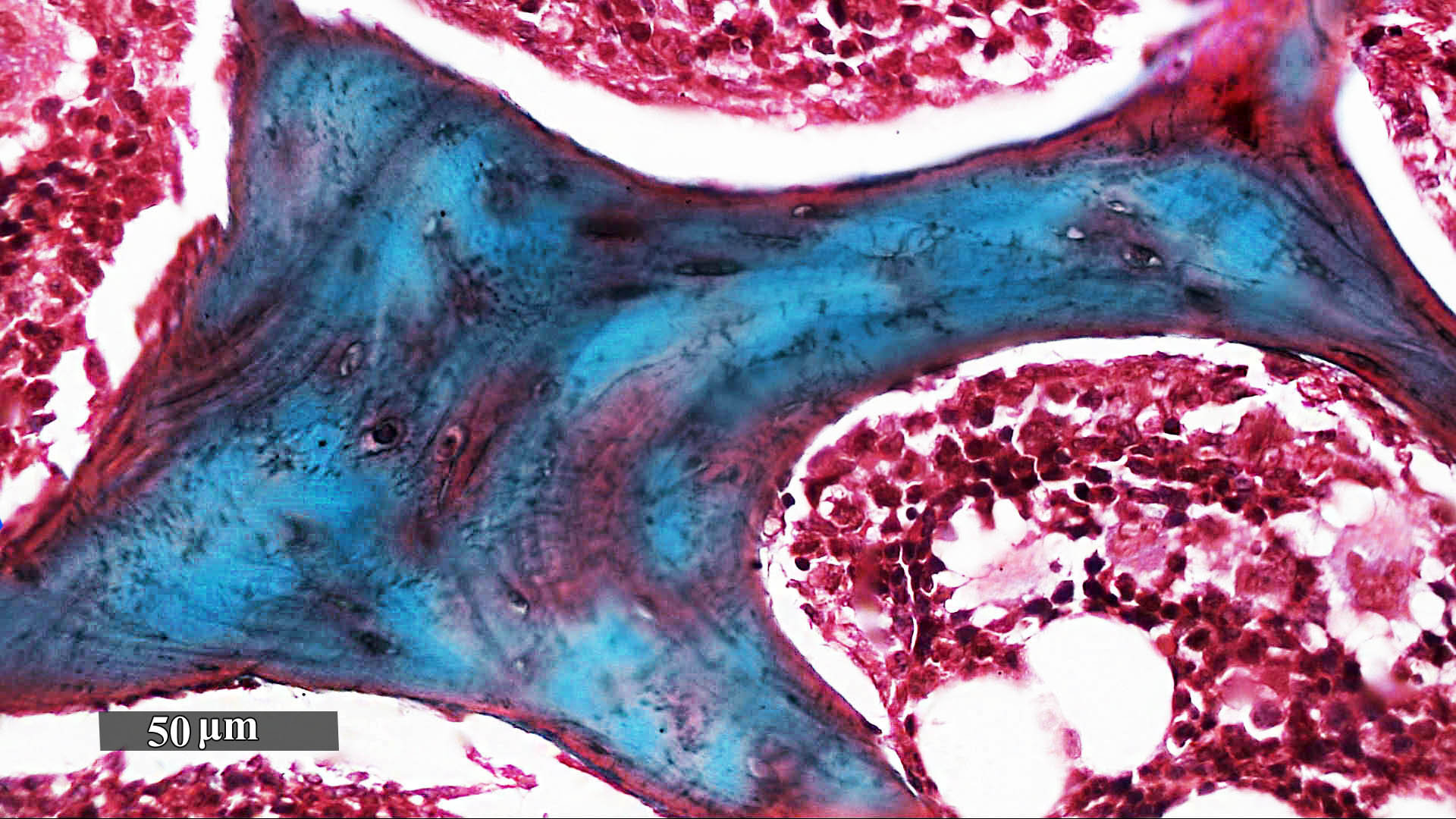

Supplement: Supplementary file 4 — Supplementary file4 (JPG 291 KB) [file 210_2024_3378_MOESM4_ESM.jpg]

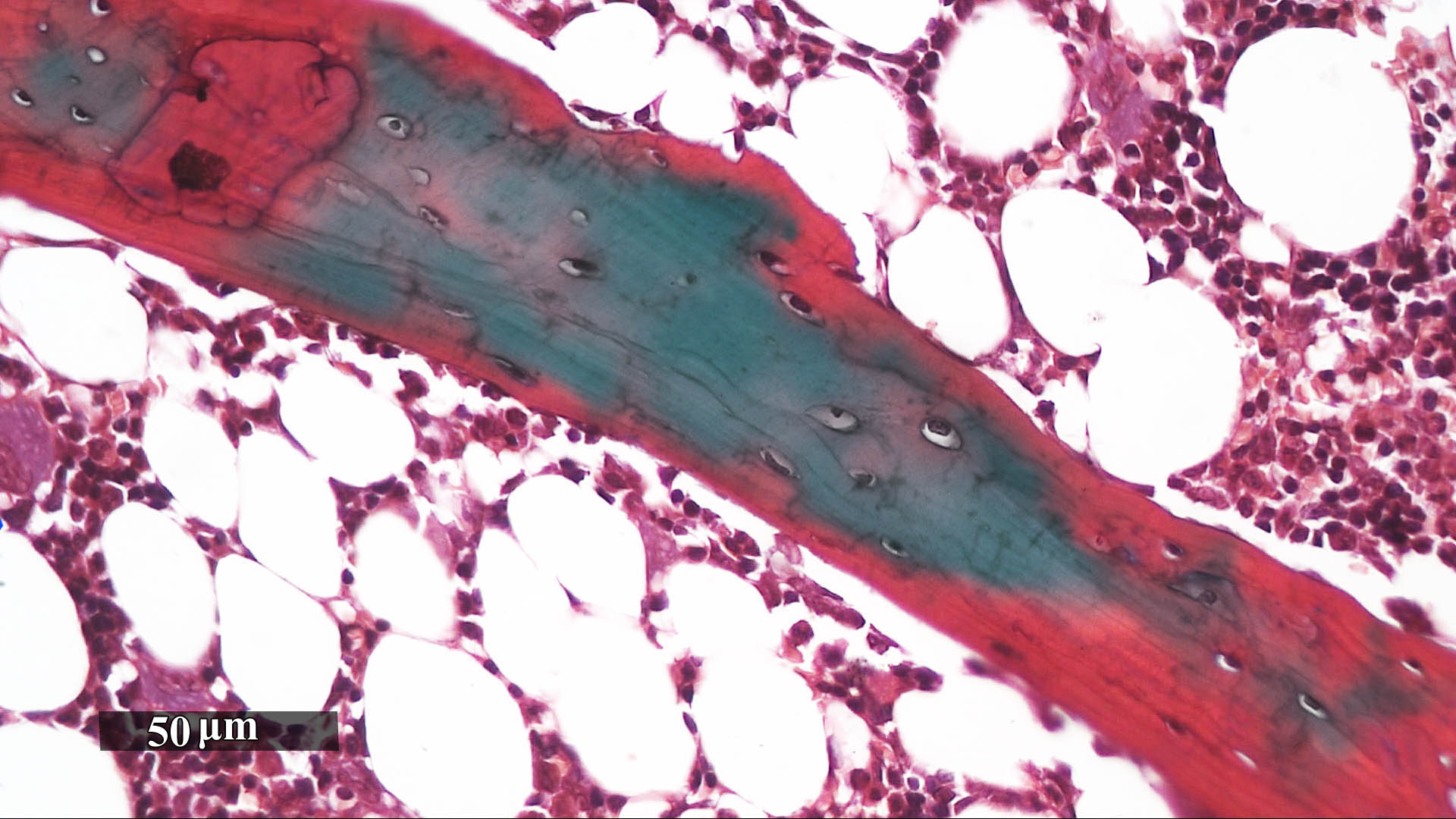

Supplement: Supplementary file 5 — Supplementary file5 (JPG 237 KB) [file 210_2024_3378_MOESM5_ESM.jpg]

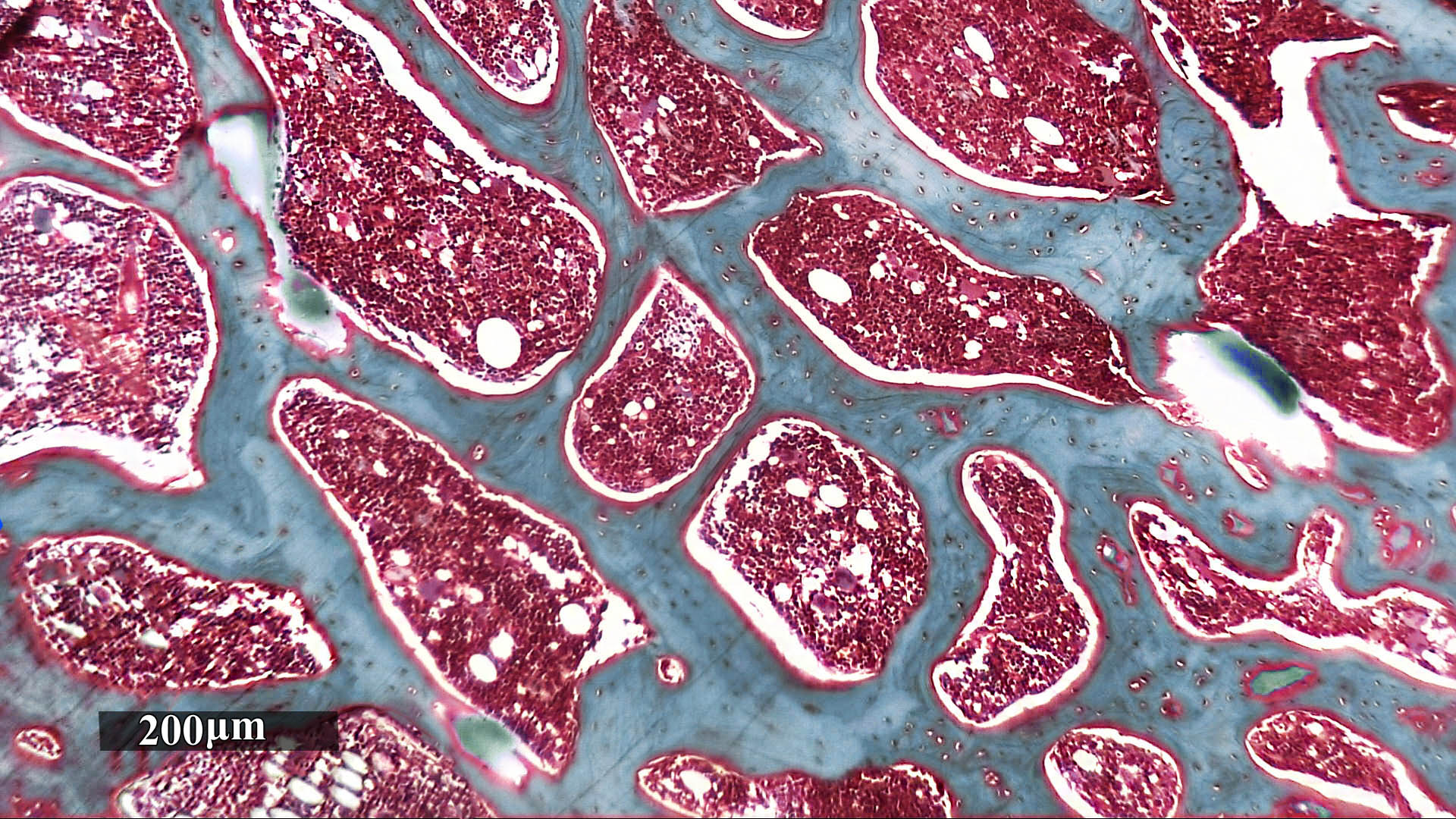

Supplement: Supplementary file 6 — Supplementary file6 (JPG 489 KB) [file 210_2024_3378_MOESM6_ESM.jpg]

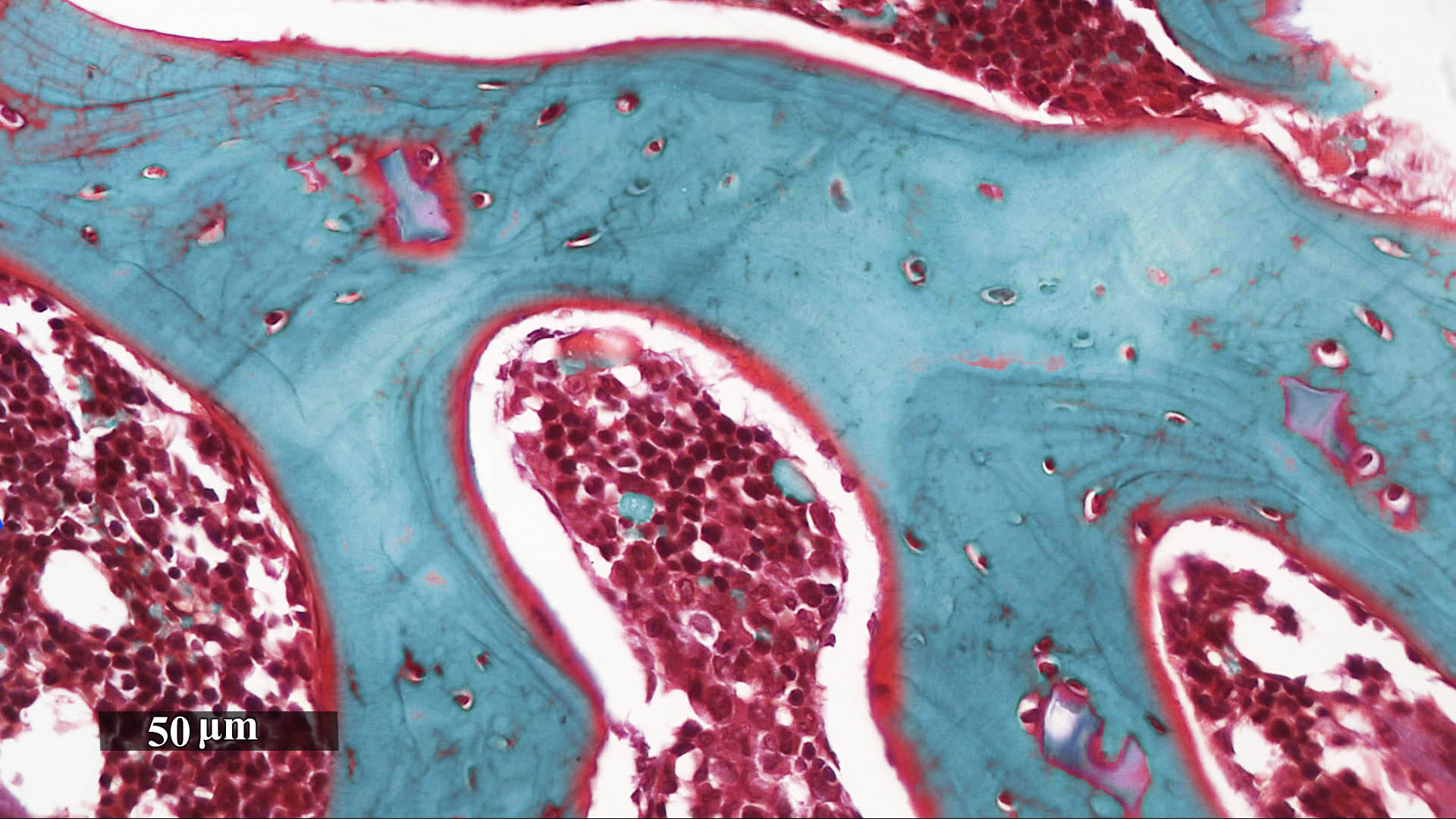

Supplement: Supplementary file 7 — Supplementary file7 (JPG 259 KB) [file 210_2024_3378_MOESM7_ESM.jpg]

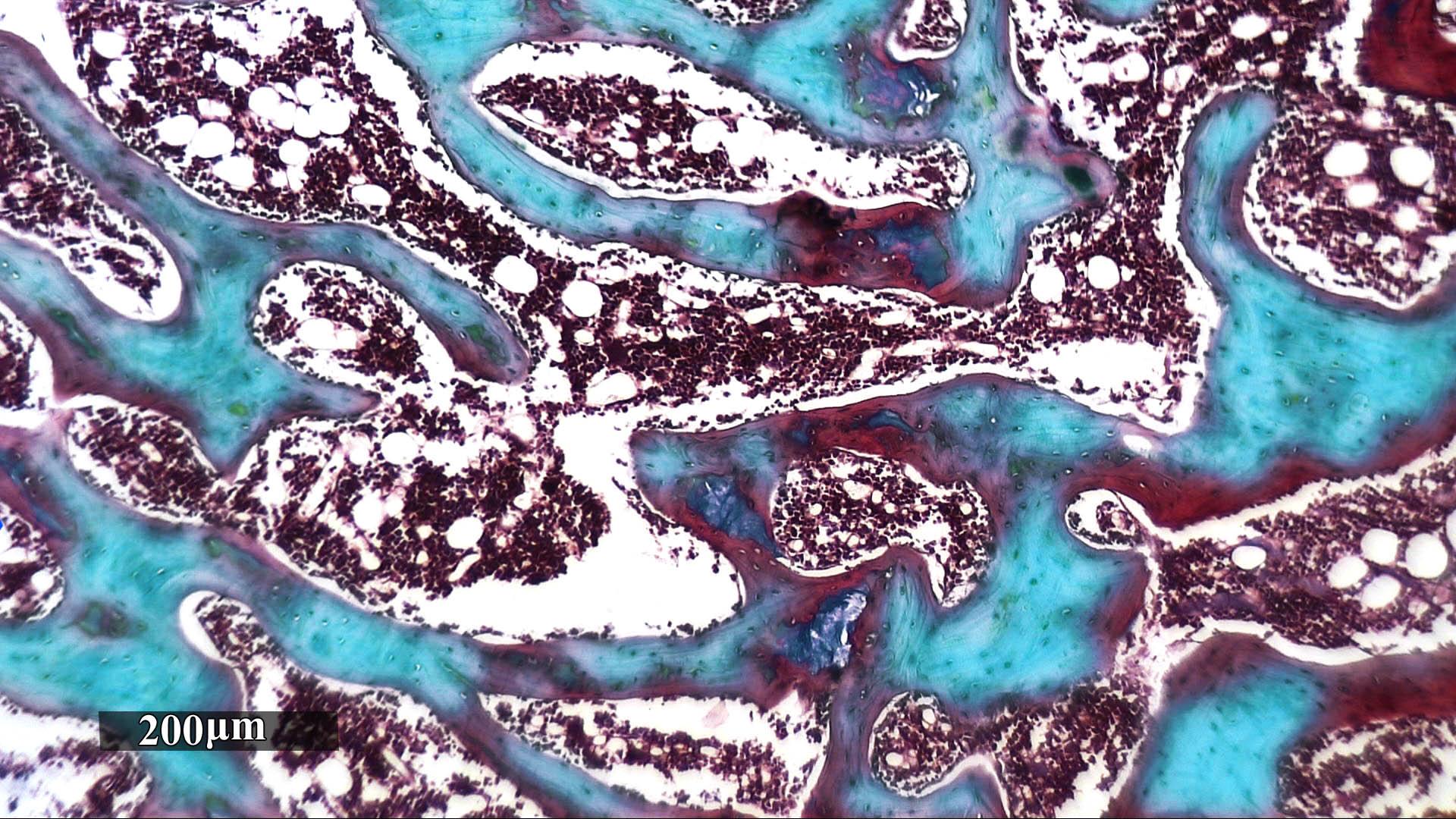

Supplement: Supplementary file 8 — Supplementary file8 (JPG 404 KB) [file 210_2024_3378_MOESM8_ESM.jpg]

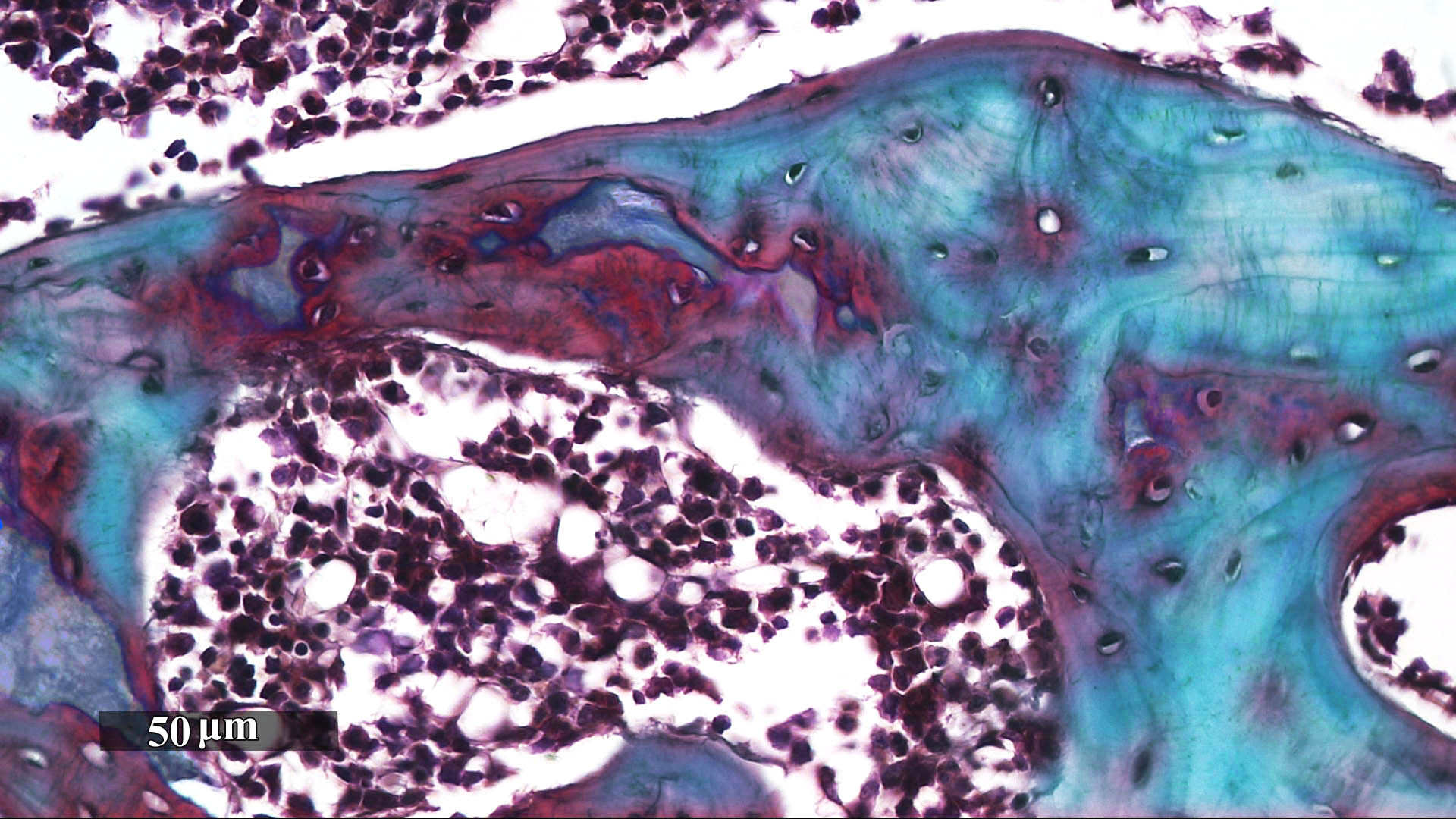

Supplement: Supplementary file 9 — Supplementary file9 (JPG 289 KB) [file 210_2024_3378_MOESM9_ESM.jpg]

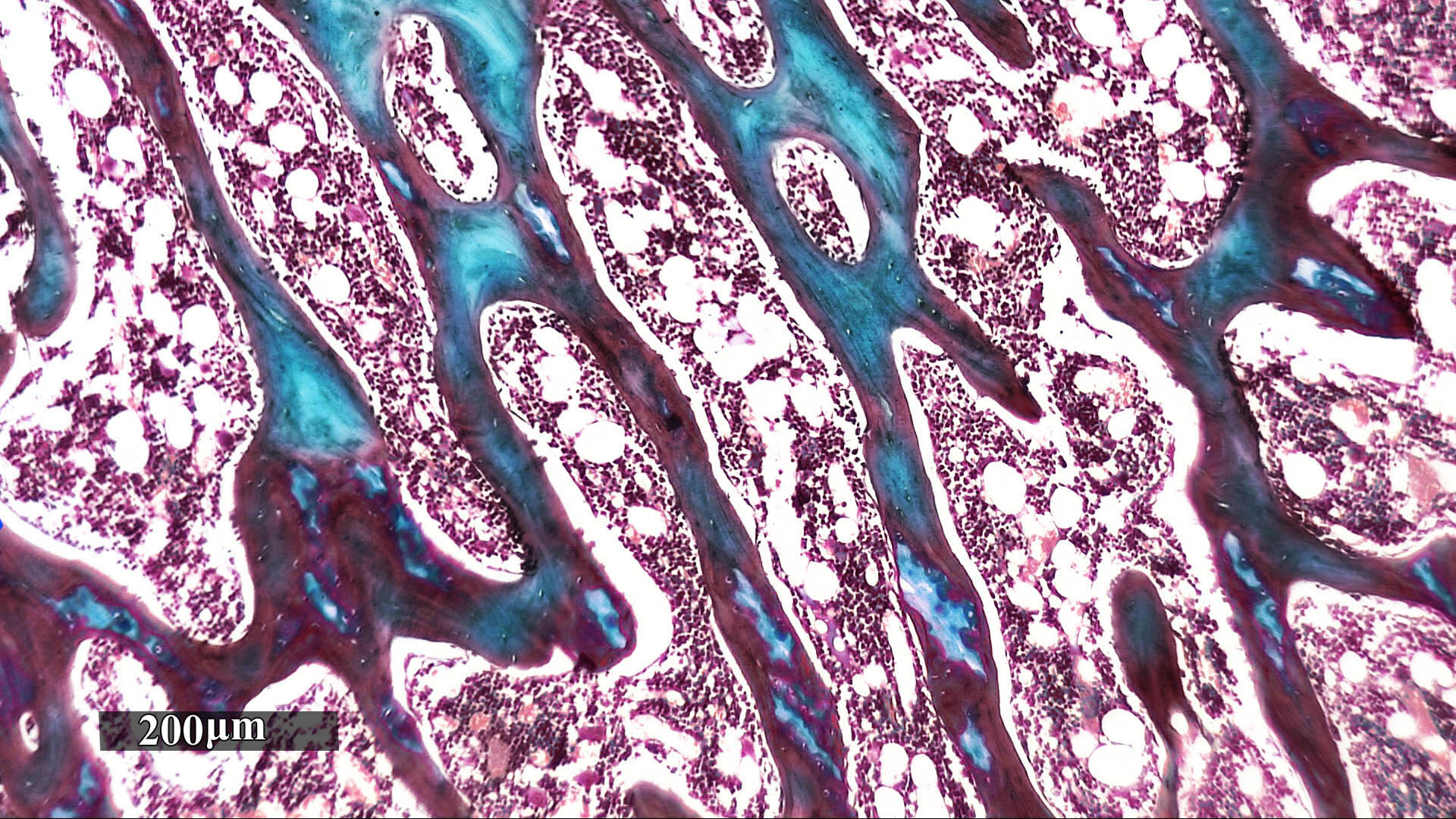

Supplement: Supplementary file 10 — Supplementary file10 (JPG 475 KB) [file 210_2024_3378_MOESM10_ESM.jpg]

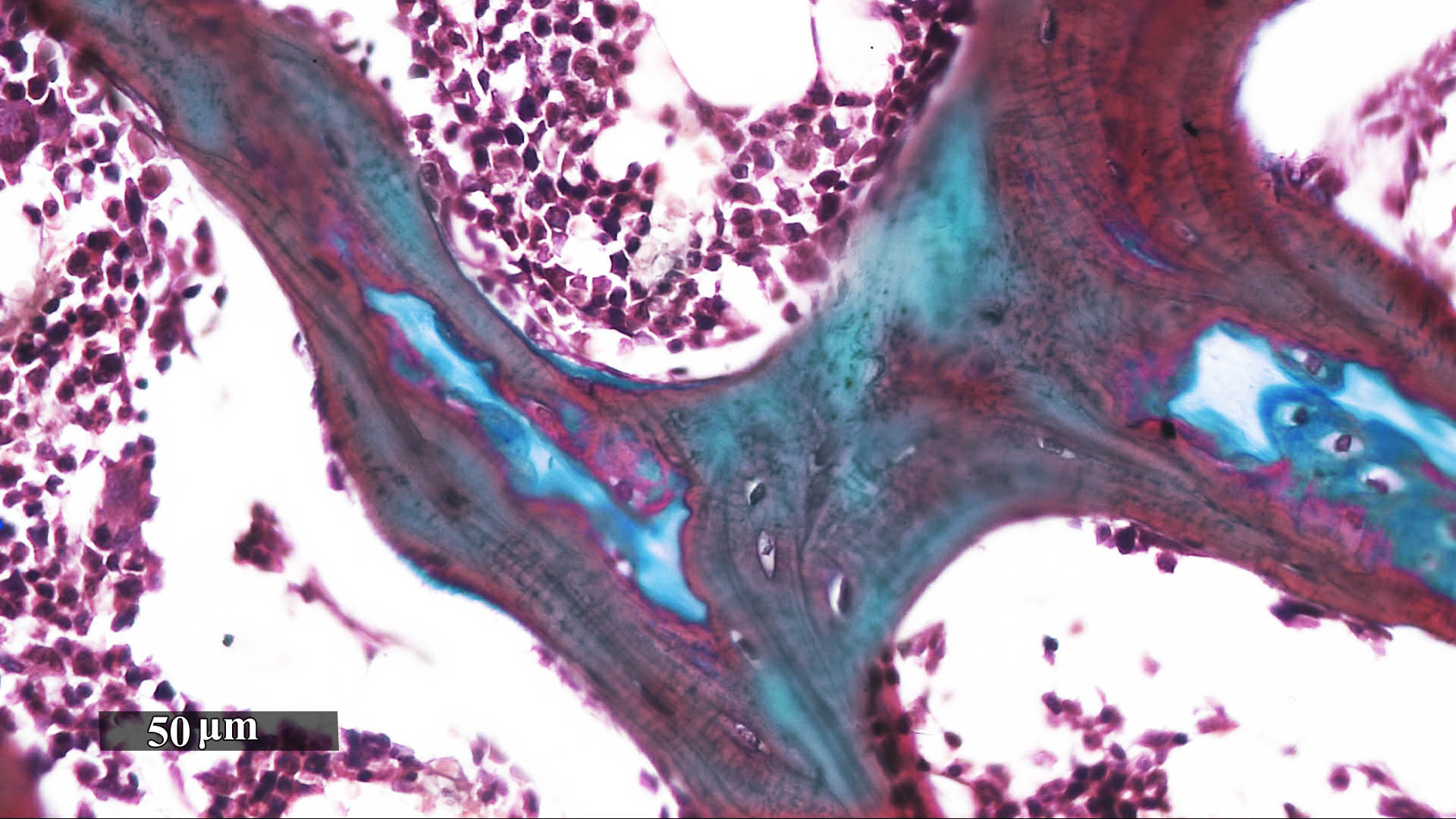

Supplement: Supplementary file 11 — Supplementary file11 (JPG 246 KB) [file 210_2024_3378_MOESM11_ESM.jpg]

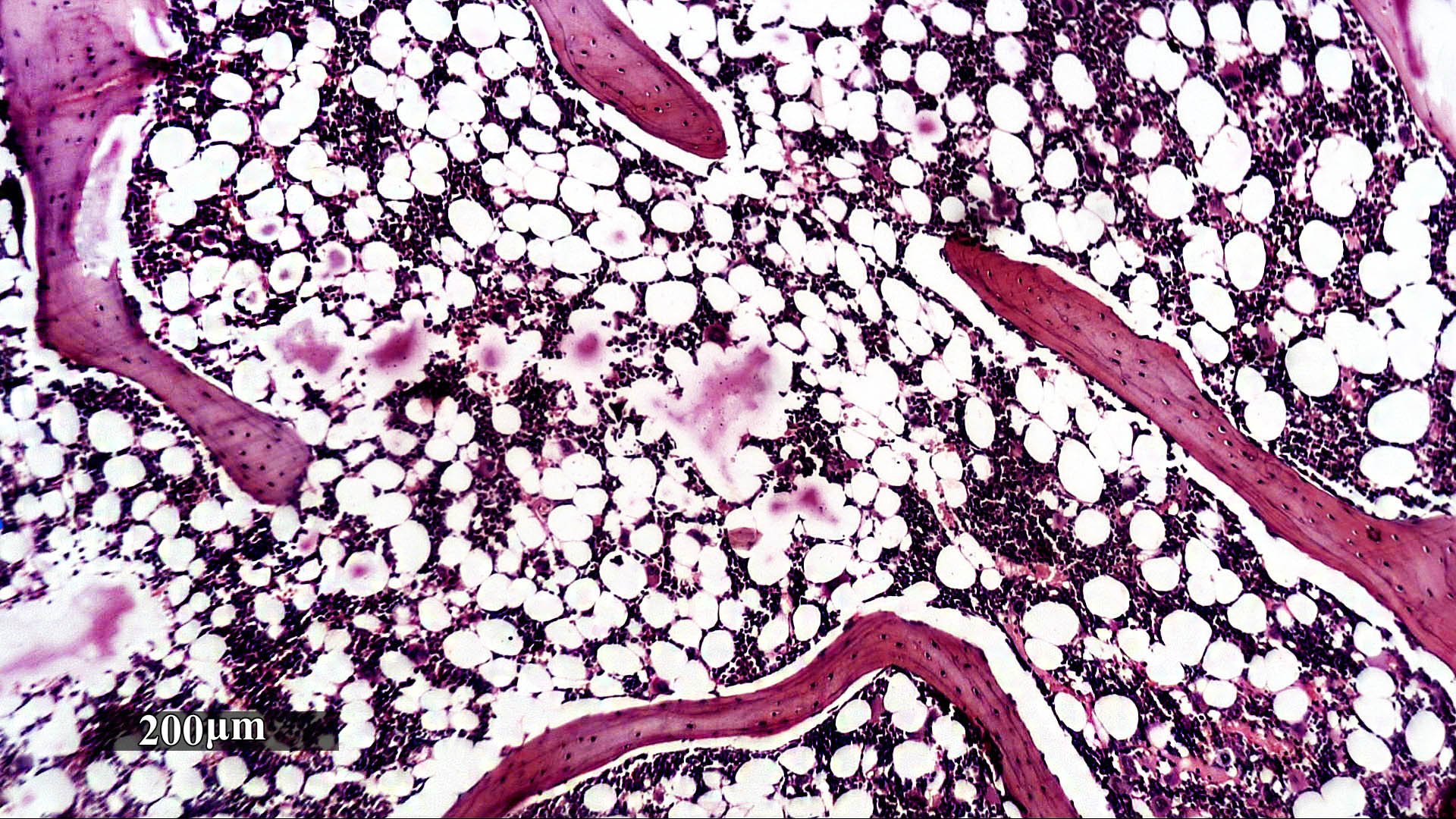

Supplement: Supplementary file 12 — Supplementary file12 (JPG 550 KB) [file 210_2024_3378_MOESM12_ESM.jpg]

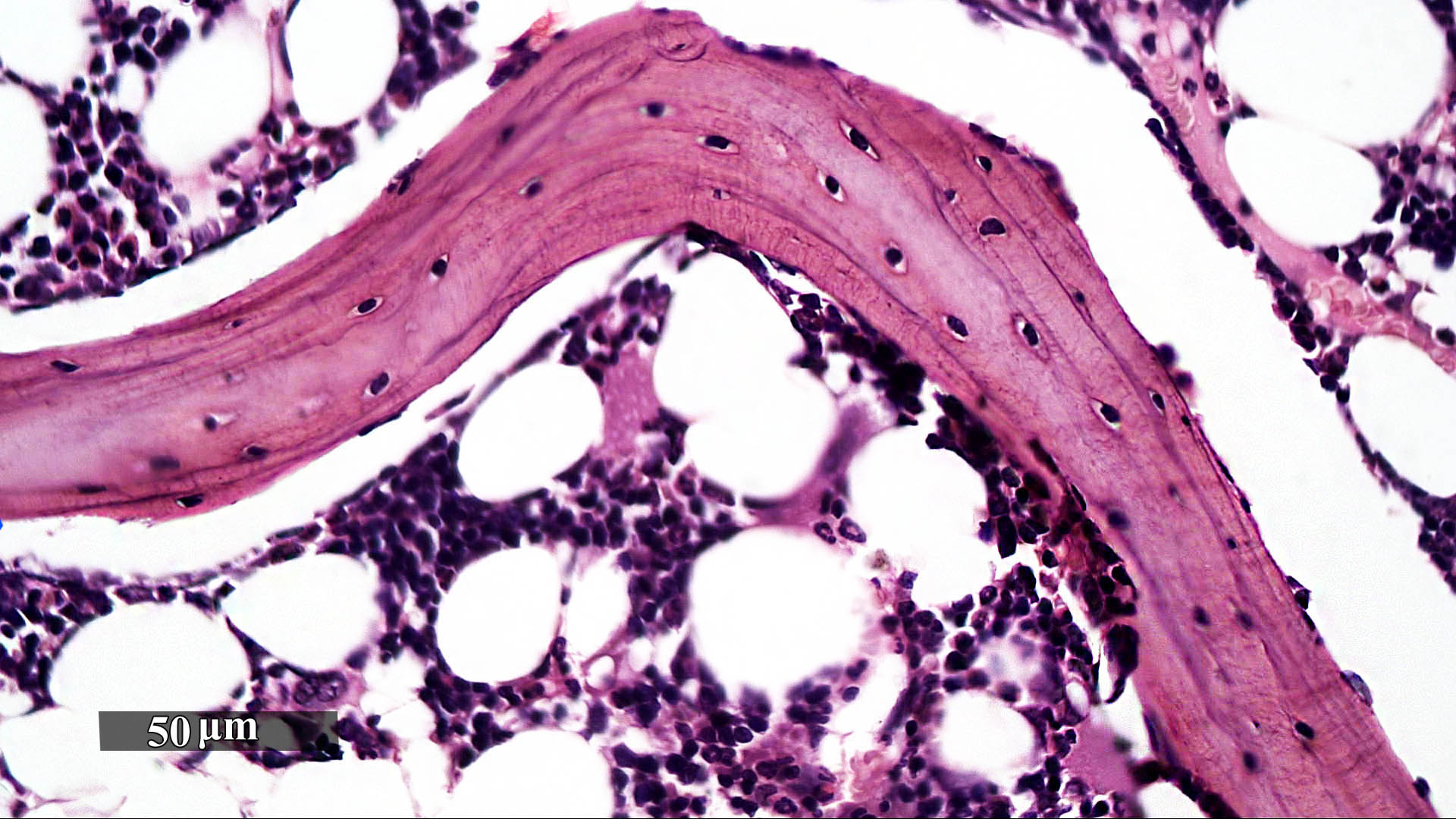

Supplement: Supplementary file 13 — Supplementary file13 (JPG 278 KB) [file 210_2024_3378_MOESM13_ESM.jpg]

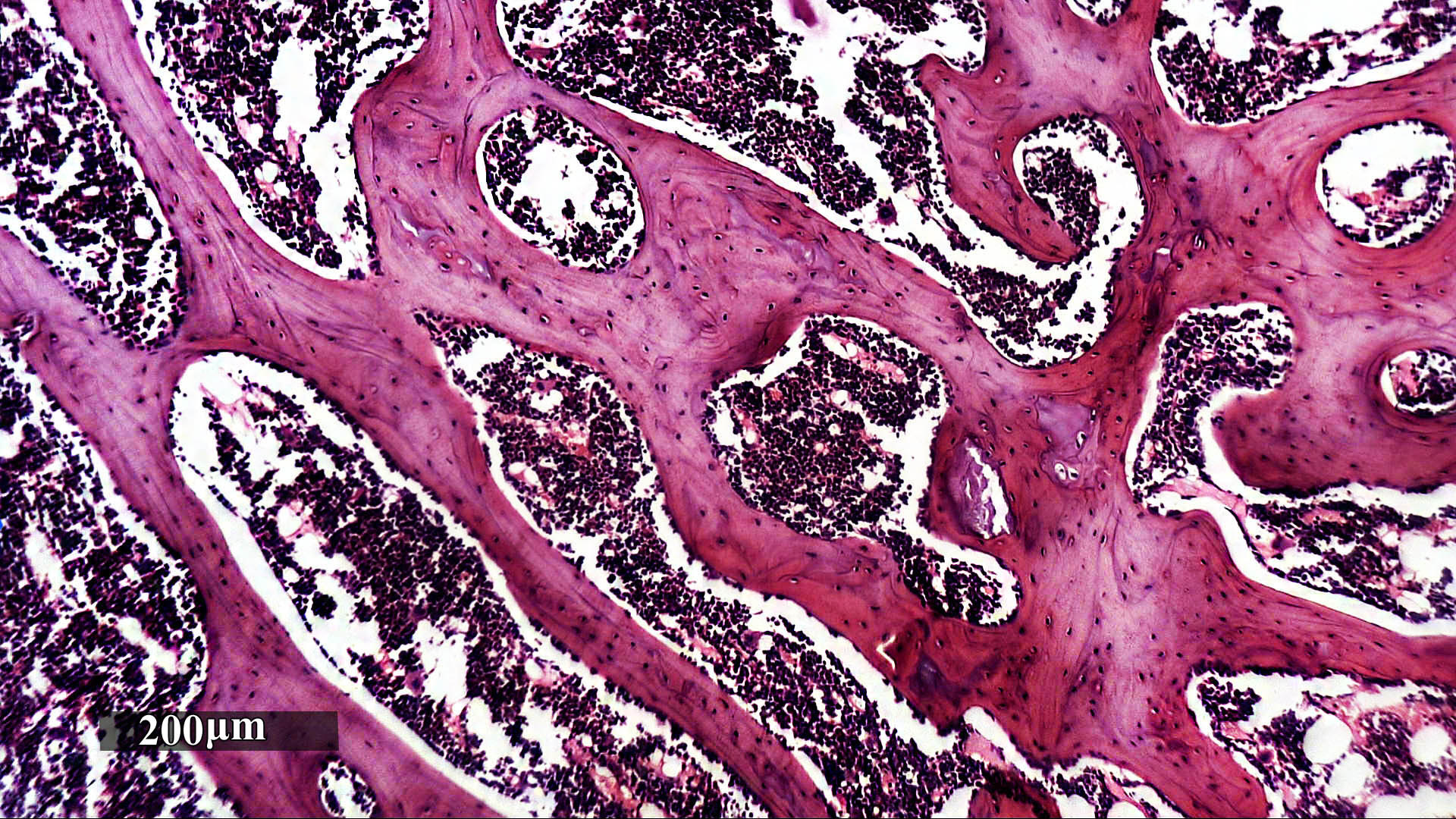

Supplement: Supplementary file 14 — Supplementary file14 (JPG 552 KB) [file 210_2024_3378_MOESM14_ESM.jpg]

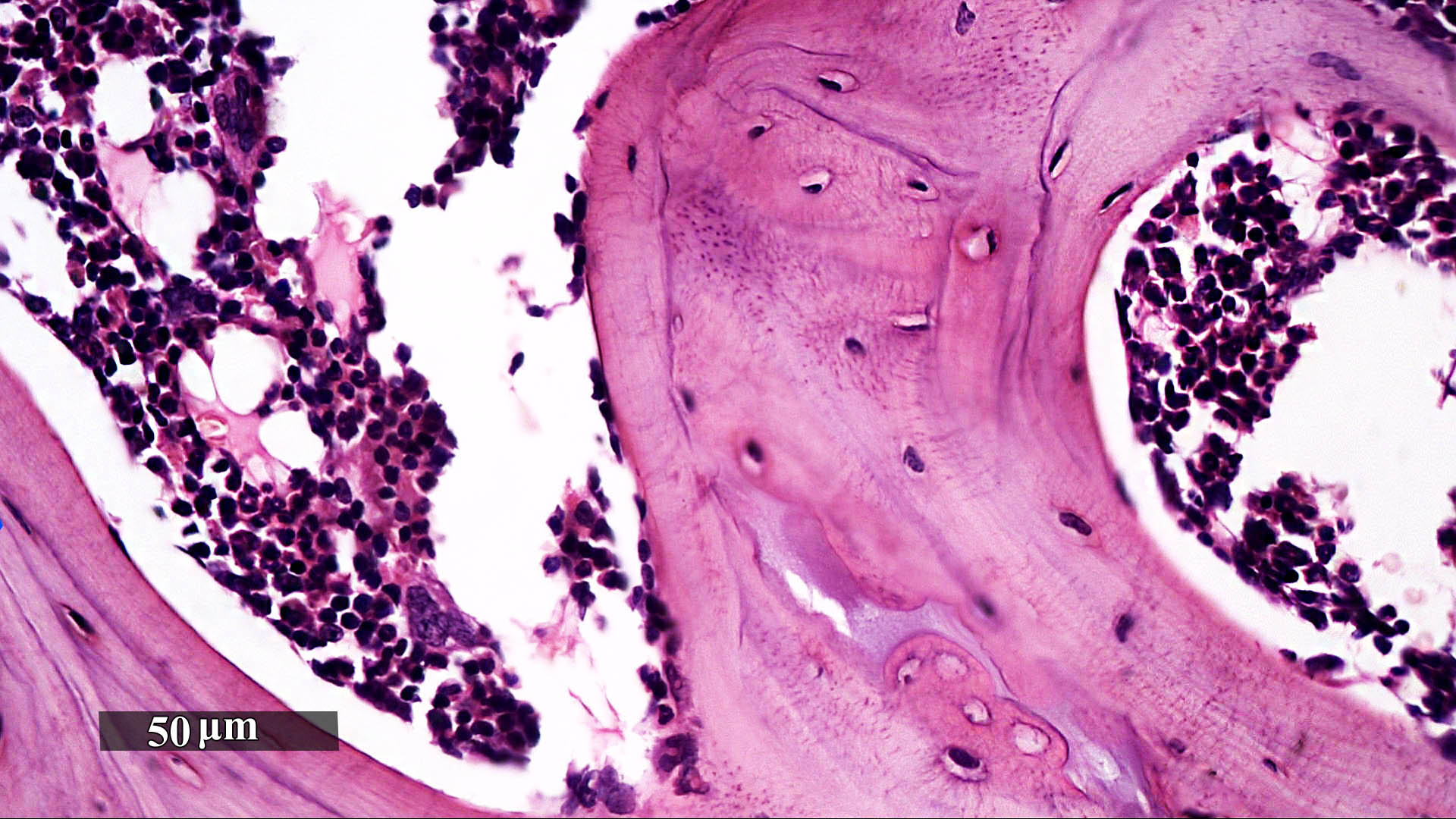

Supplement: Supplementary file 15 — Supplementary file15 (JPG 319 KB) [file 210_2024_3378_MOESM15_ESM.jpg]

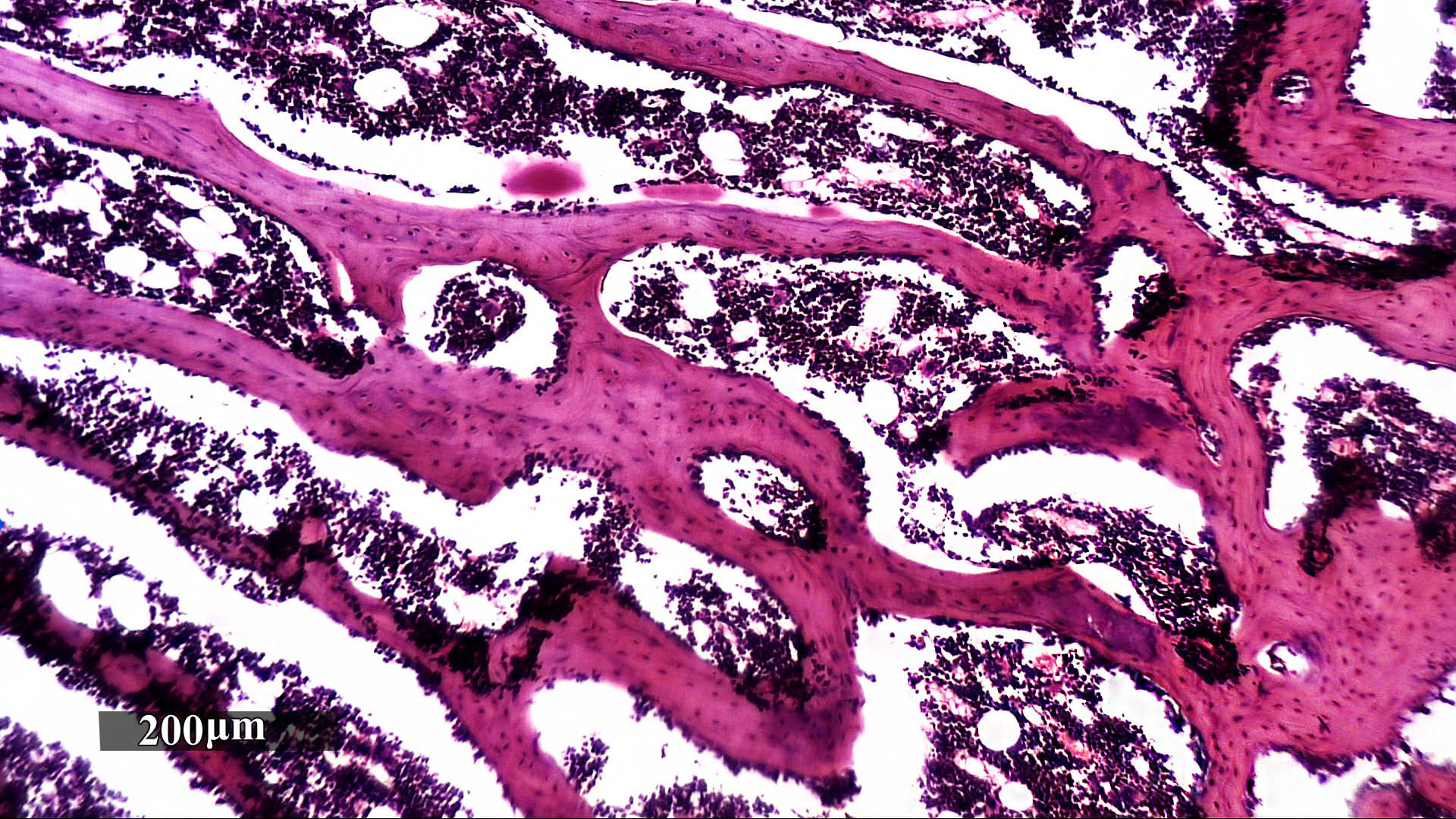

Supplement: Supplementary file 16 — Supplementary file16 (JPG 479 KB) [file 210_2024_3378_MOESM16_ESM.jpg]

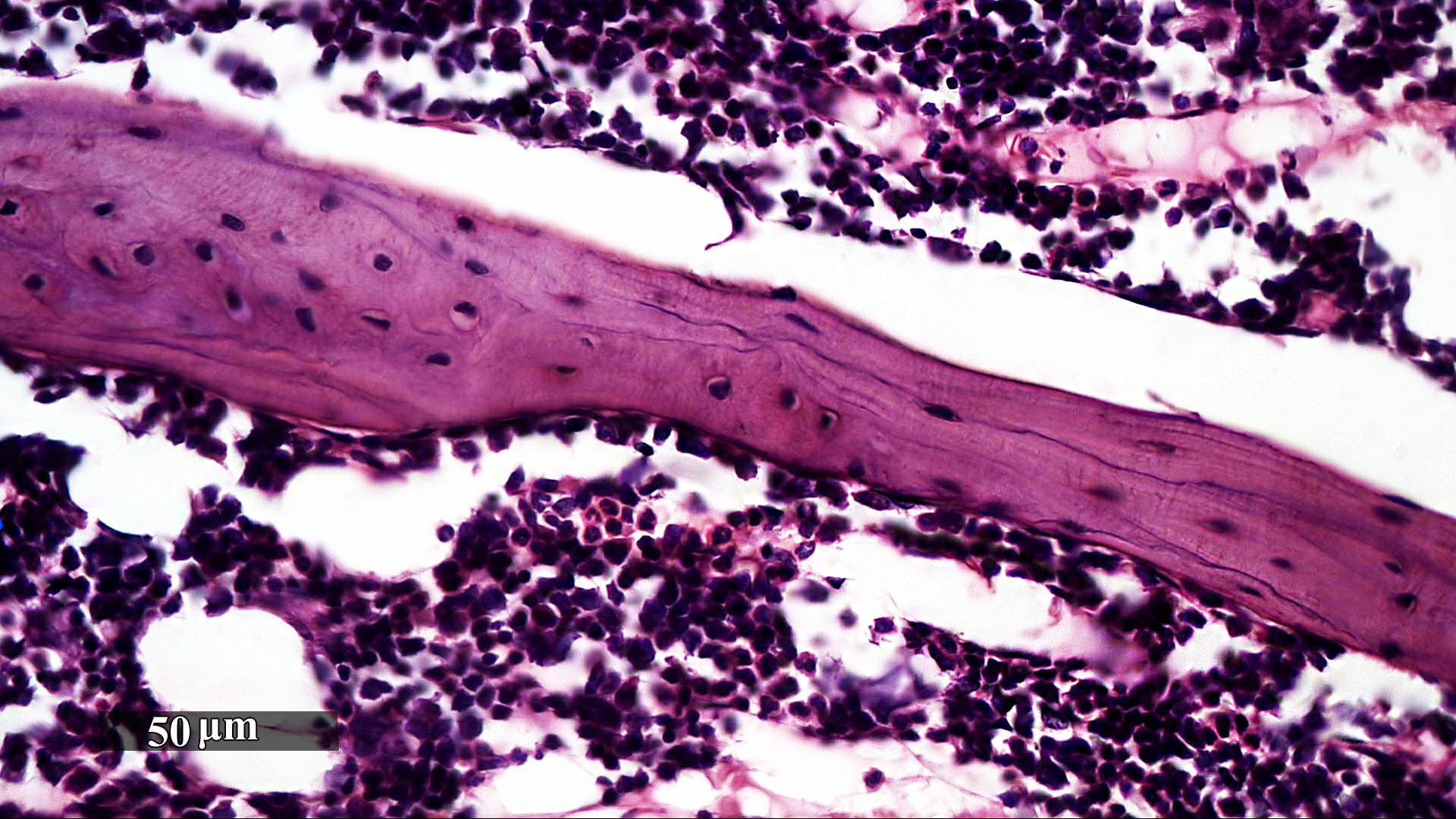

Supplement: Supplementary file 17 — Supplementary file17 (JPG 310 KB) [file 210_2024_3378_MOESM17_ESM.jpg]

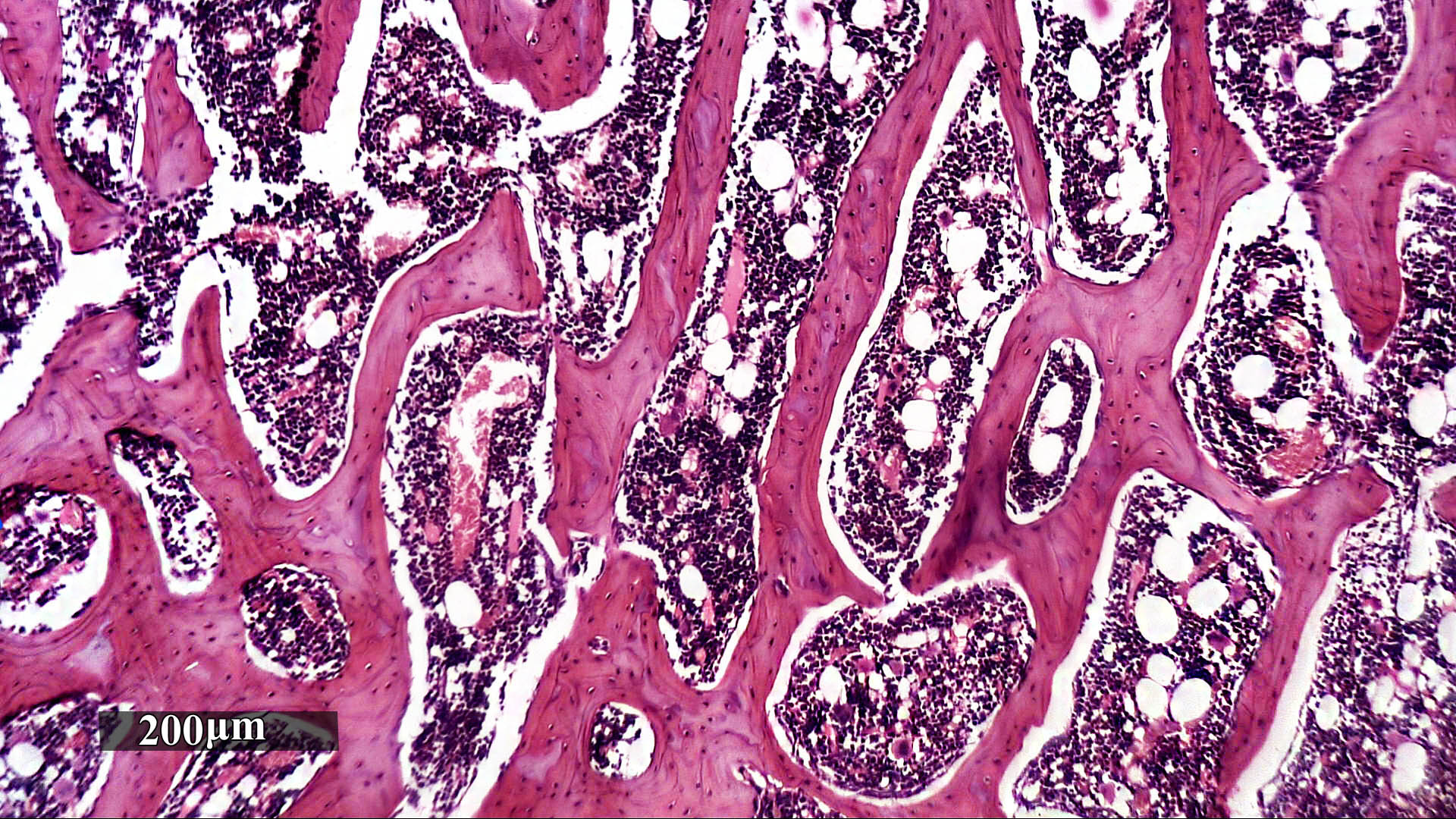

Supplement: Supplementary file 18 — Supplementary file18 (JPG 542 KB) [file 210_2024_3378_MOESM18_ESM.jpg]
