# Supplementary material for: The crucial role of beta-catenin in the osteoprotective effect of semaglutide in an ovariectomized rat model of osteoporosis
Source: Naunyn Schmiedebergs Arch Pharmacol. 2024 Sep 10;398(3):2677–93. doi: 10.1007/s00210-024-03378-z (PMC11920005; doi:10.1007/s00210-024-03378-z)
Supplement: Supplementary file 19 — Supplementary file19 (PDF 1568 KB) [file 210_2024_3378_MOESM19_ESM.pdf]

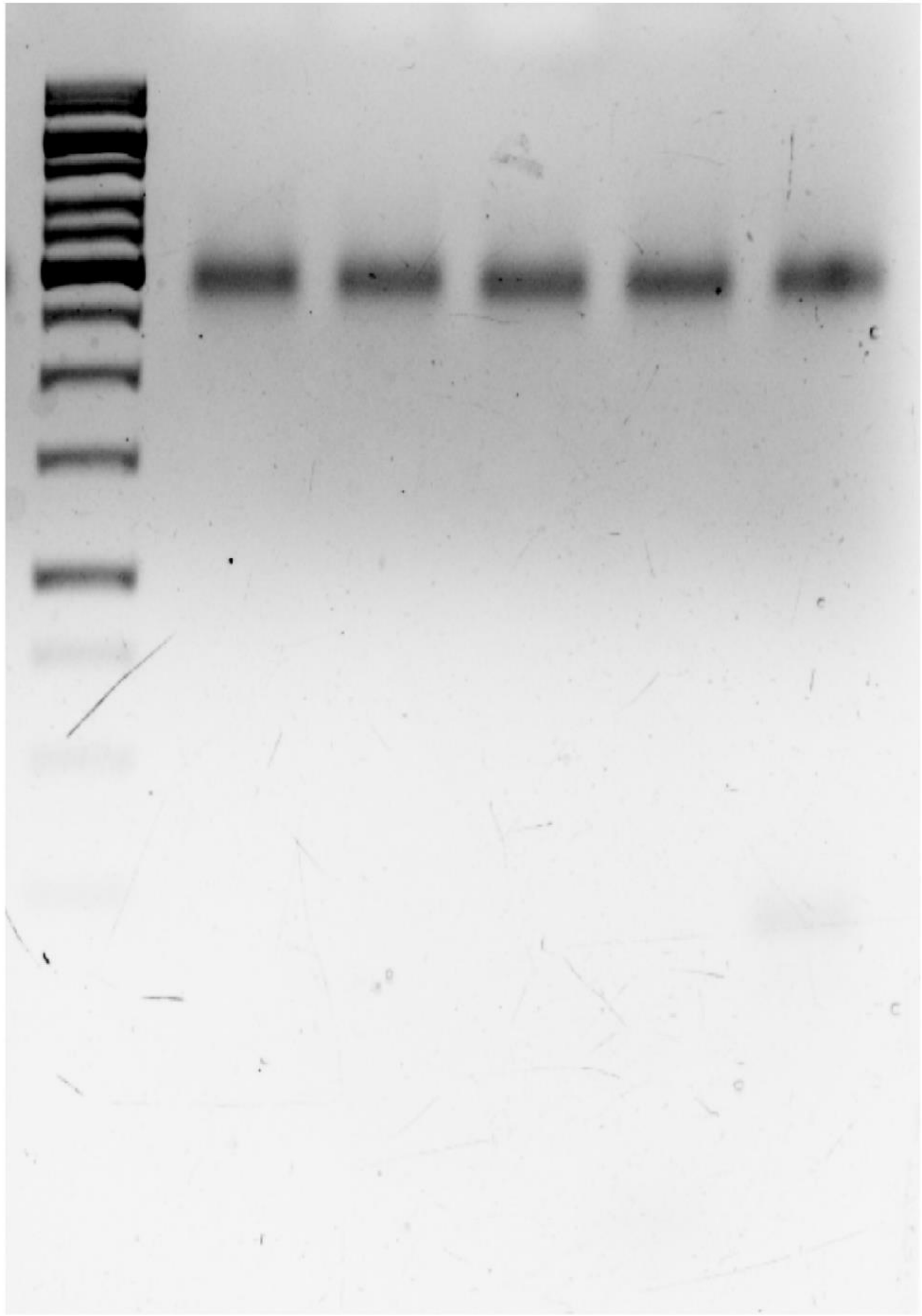

BETA ACTIN

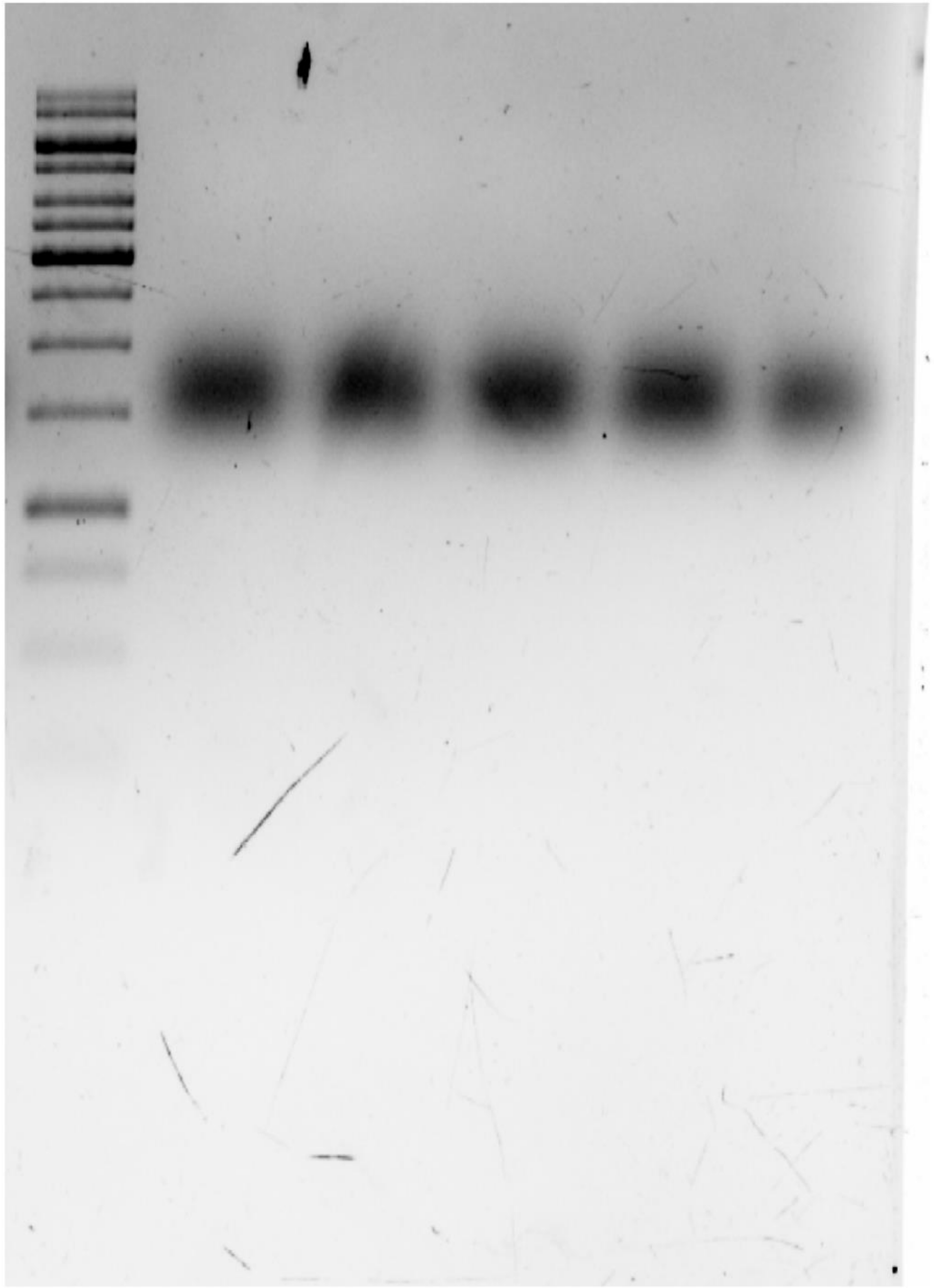

BETA ACTIN

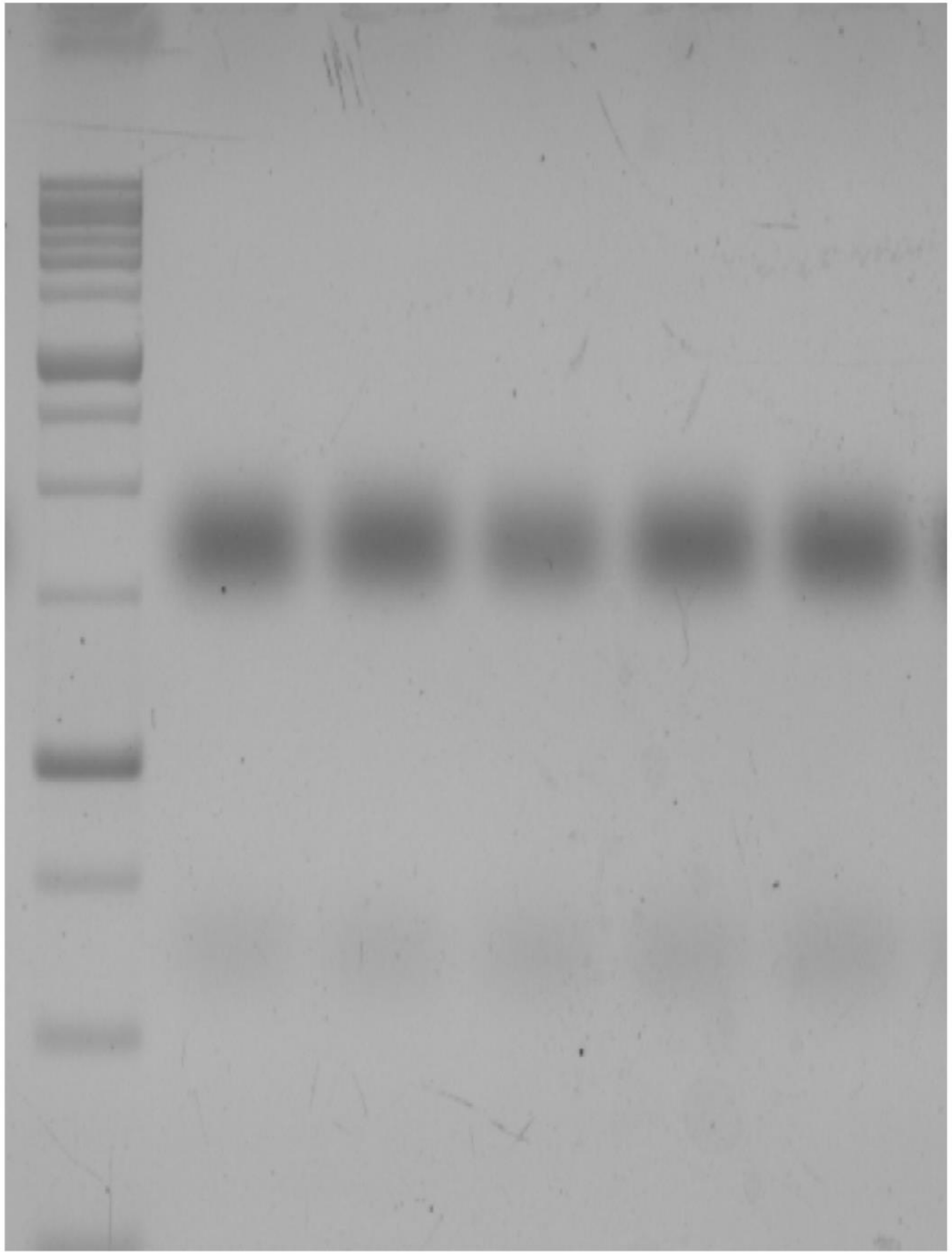

BETA ACTIN

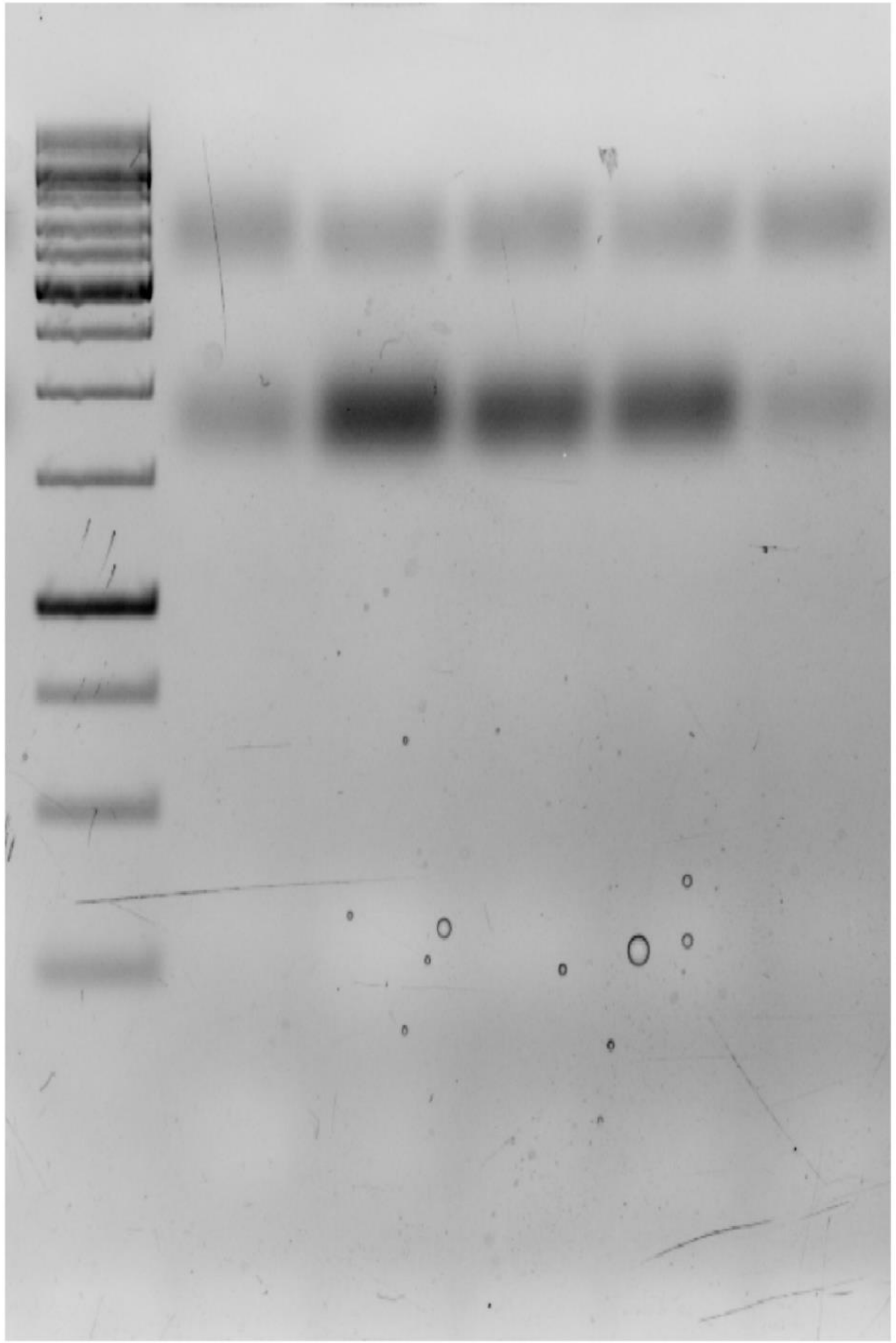

BMP

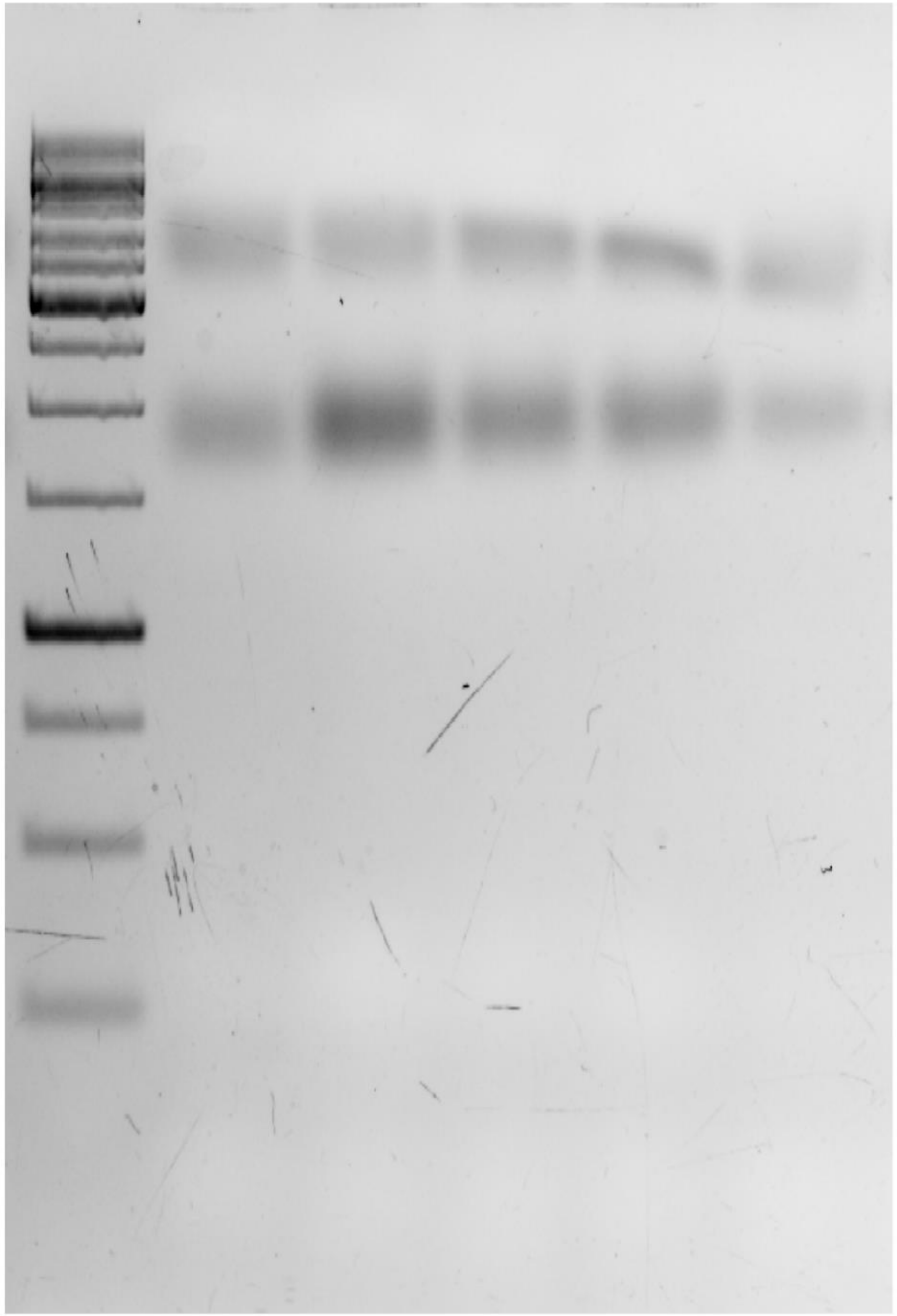

BMP

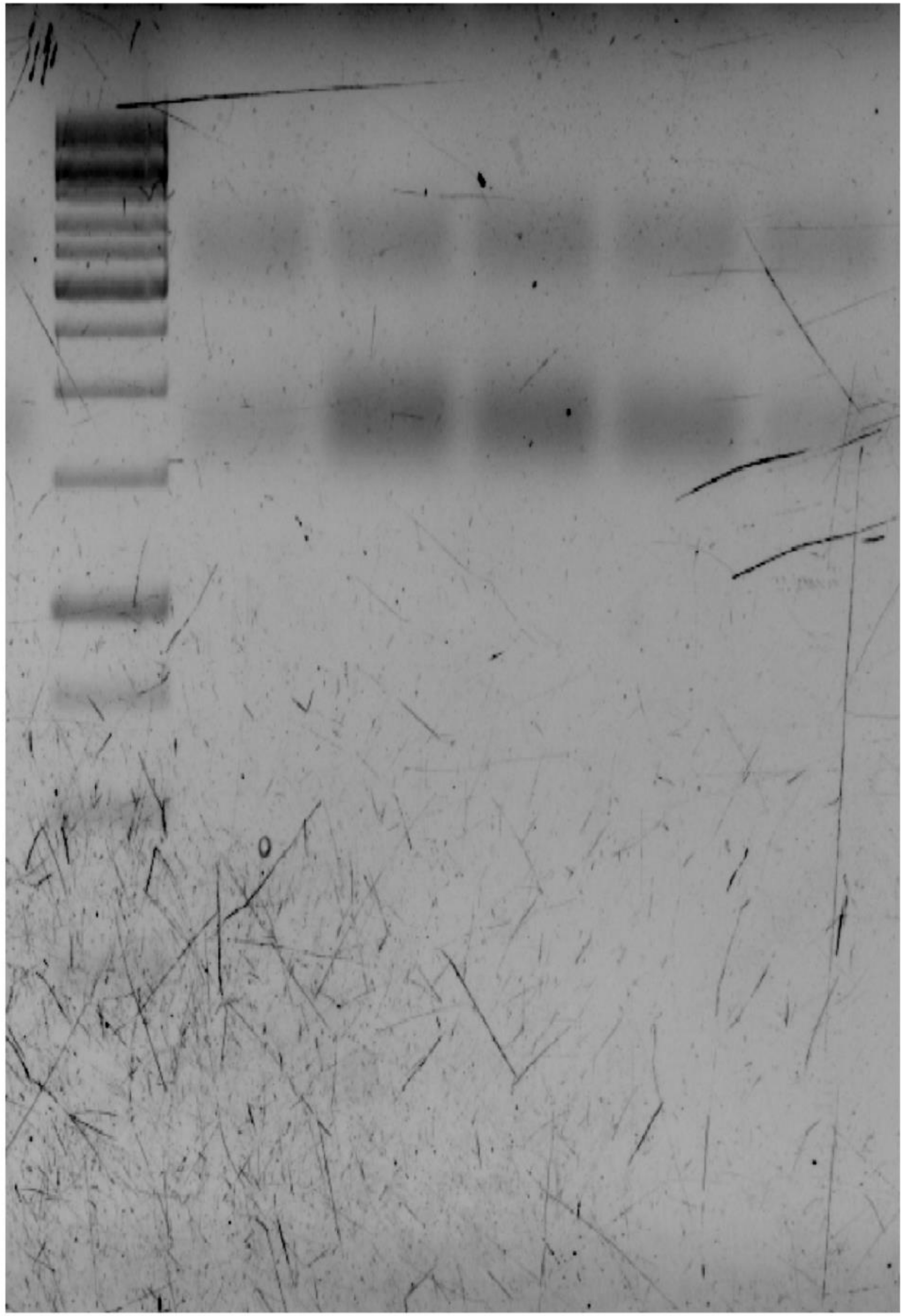

BMP

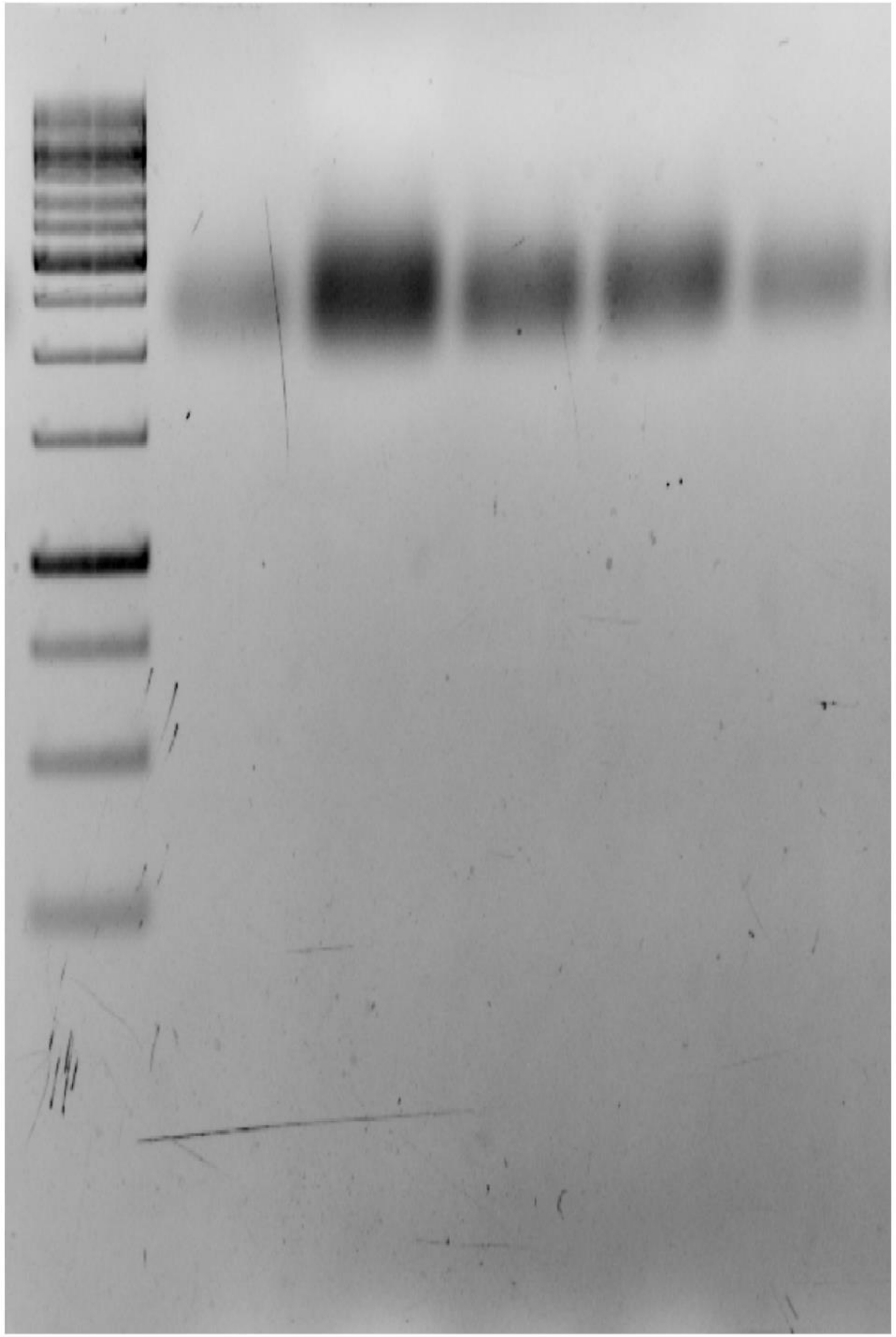

P-CATENIN

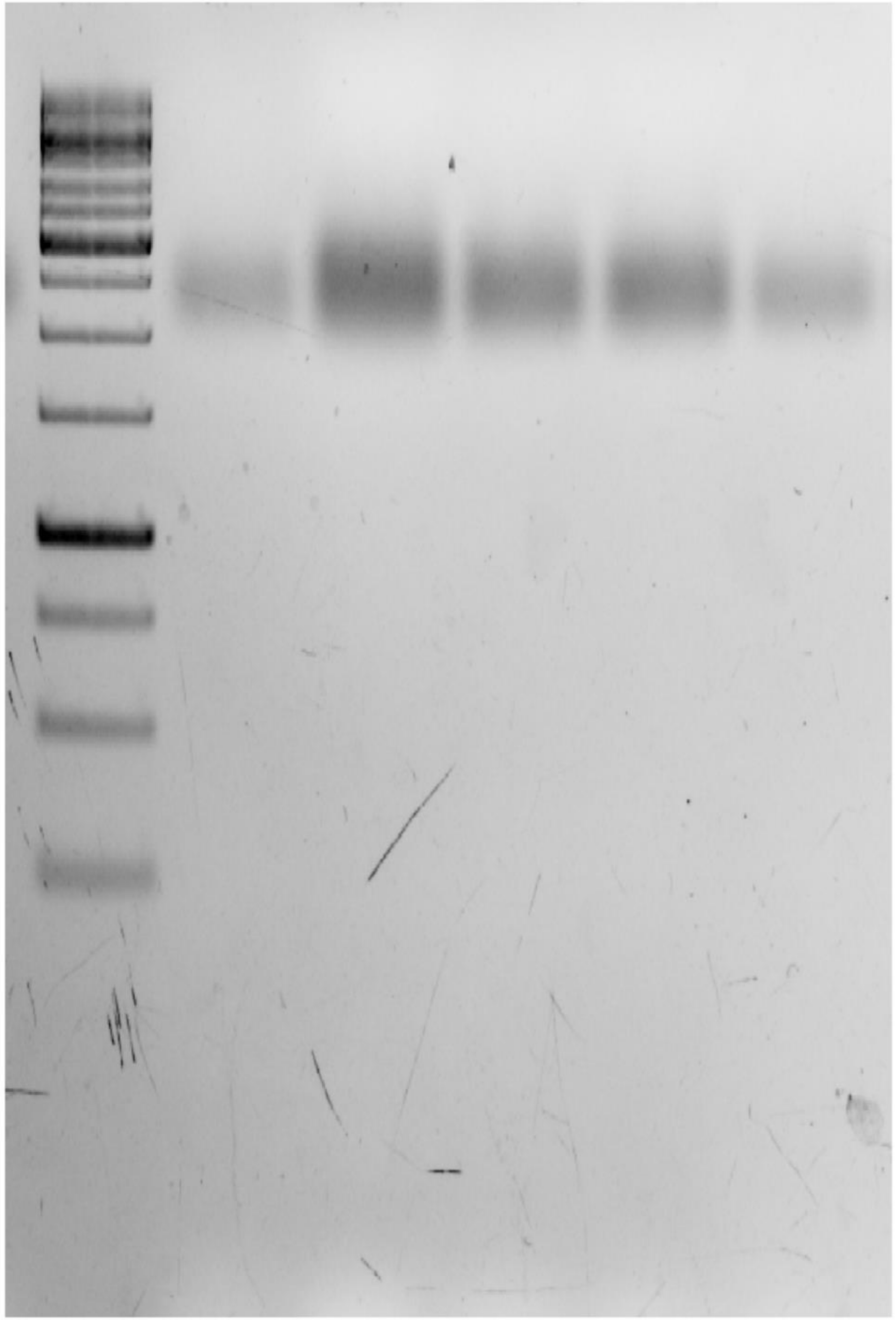

P-CATENIN

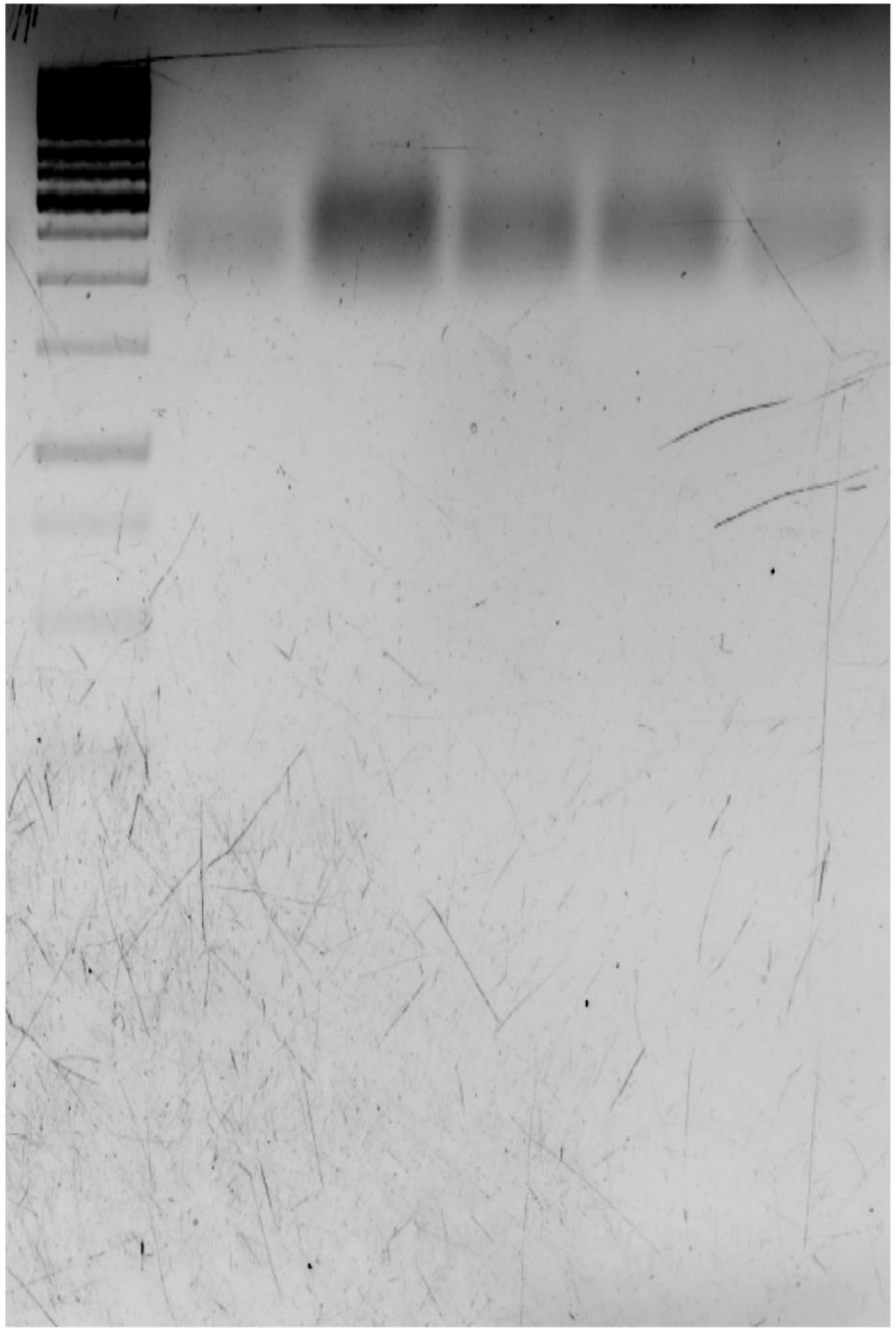

P-CATENIN

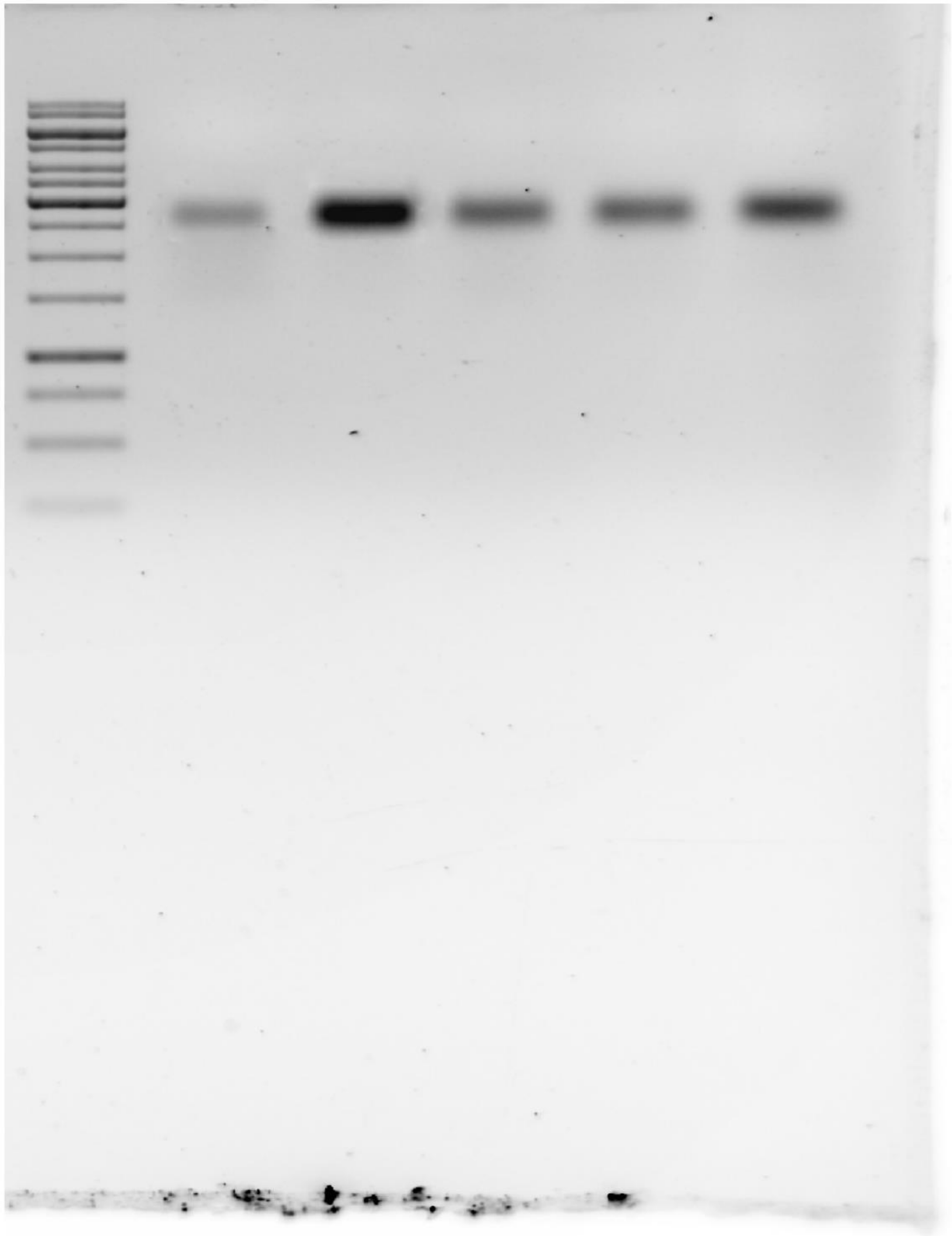

P-AKT

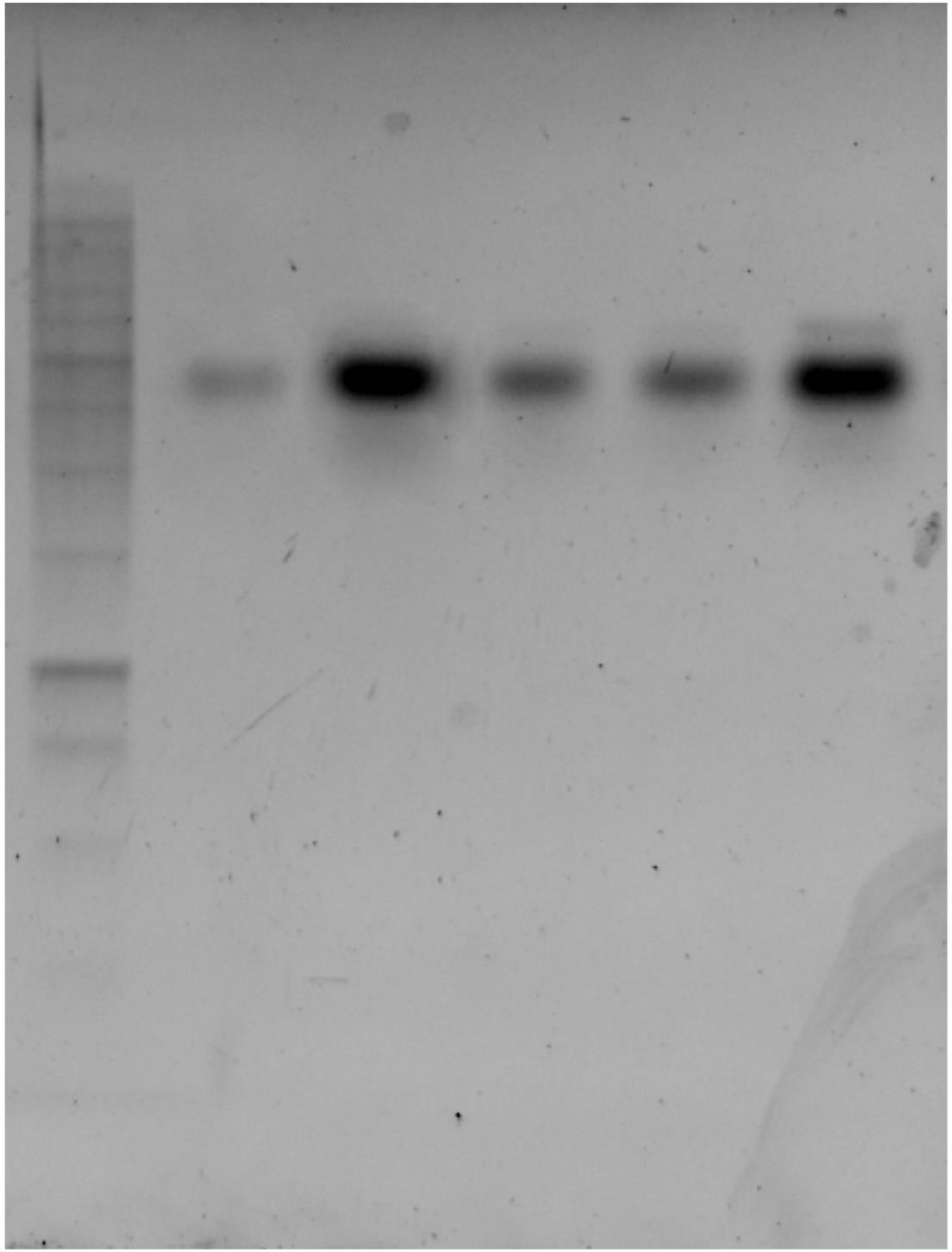

P-AKT

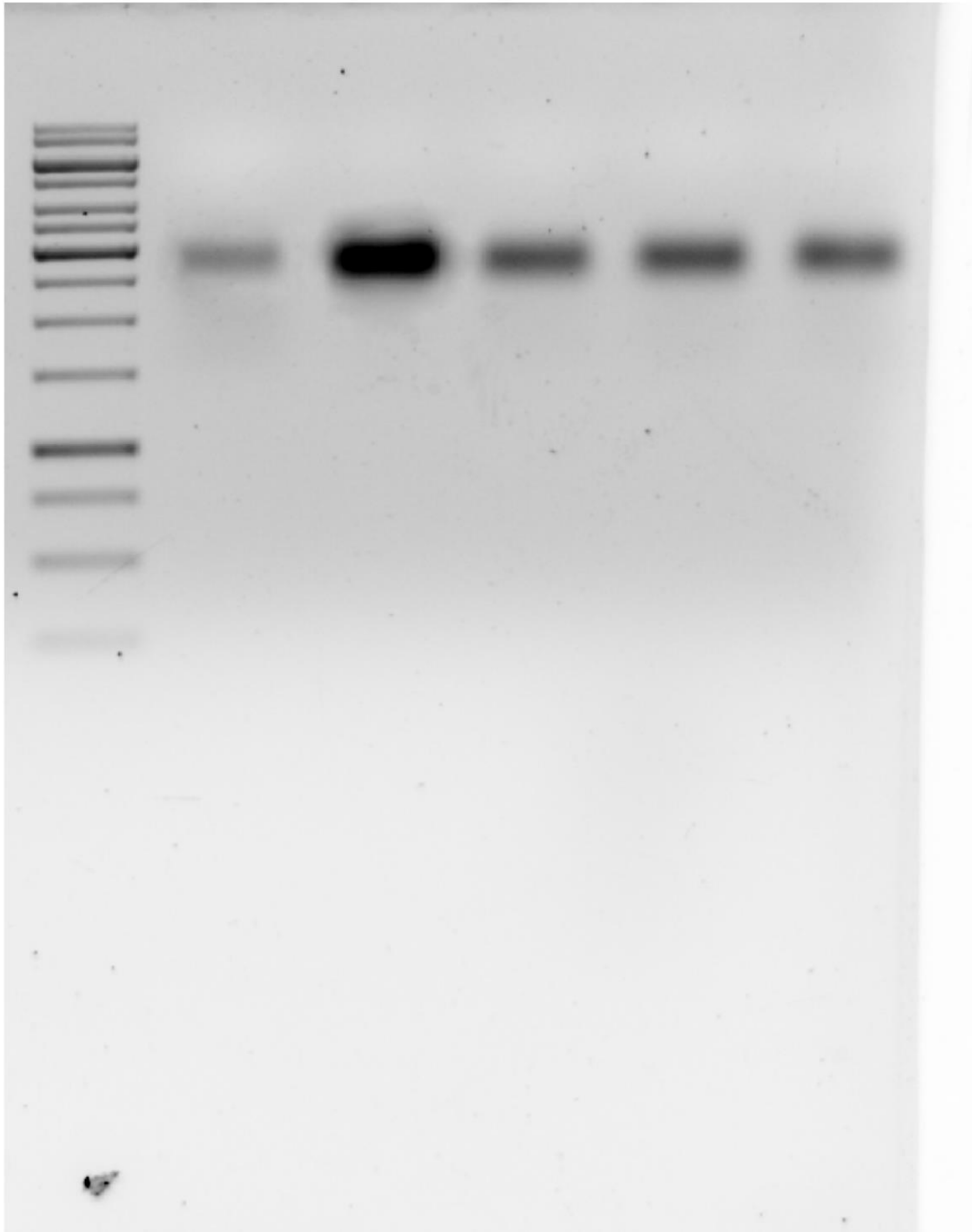

P-AKT

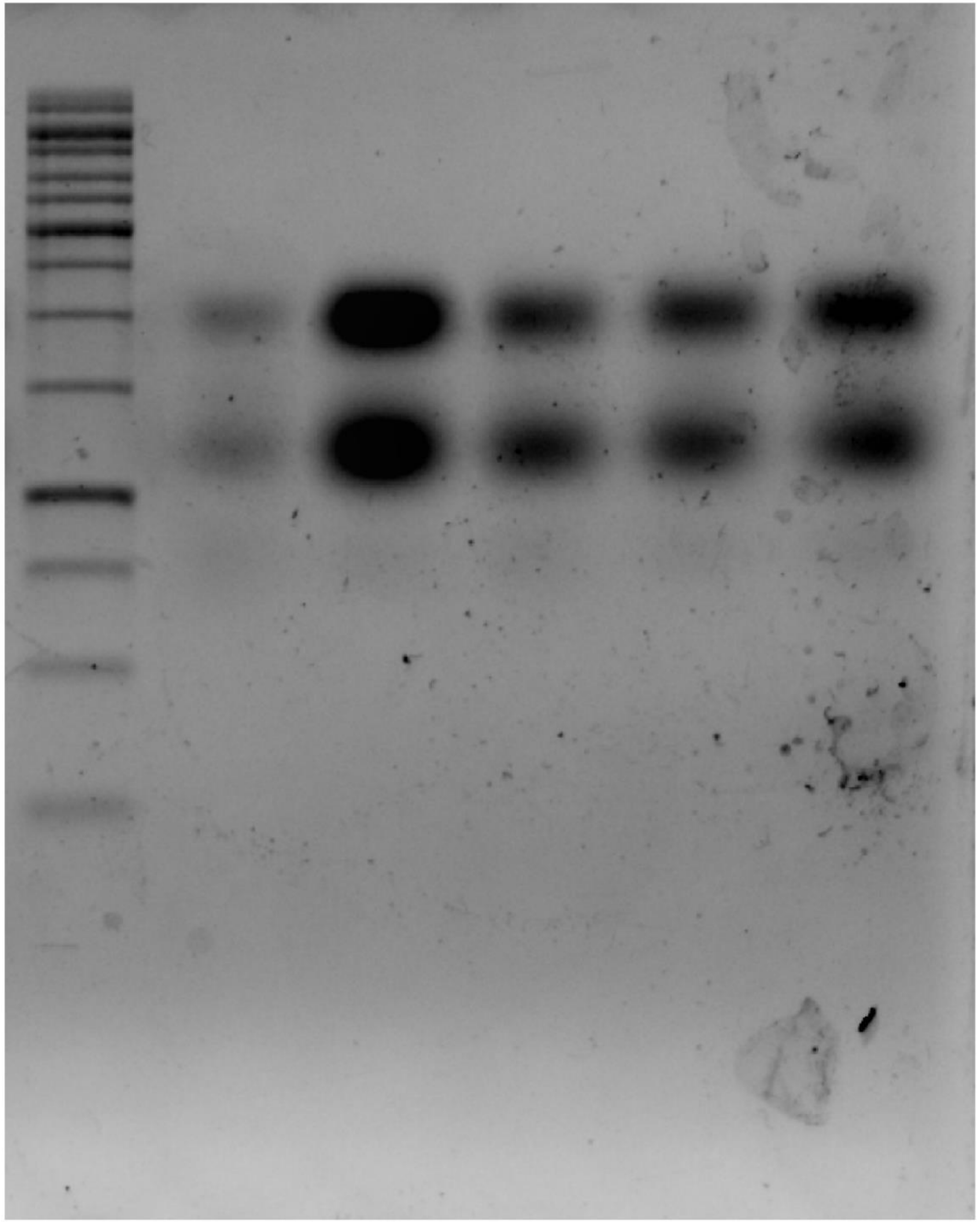

PKA

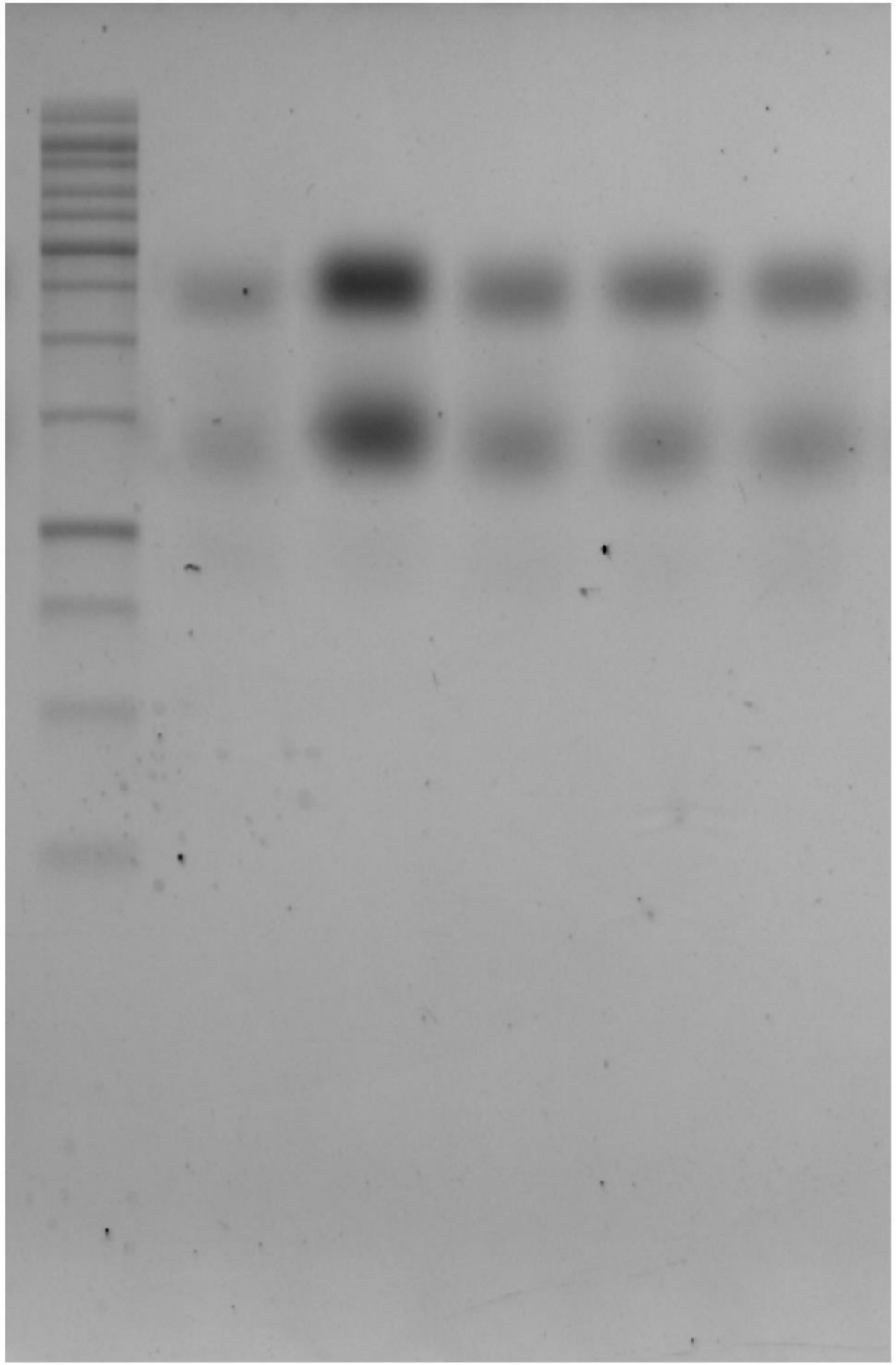

PKA

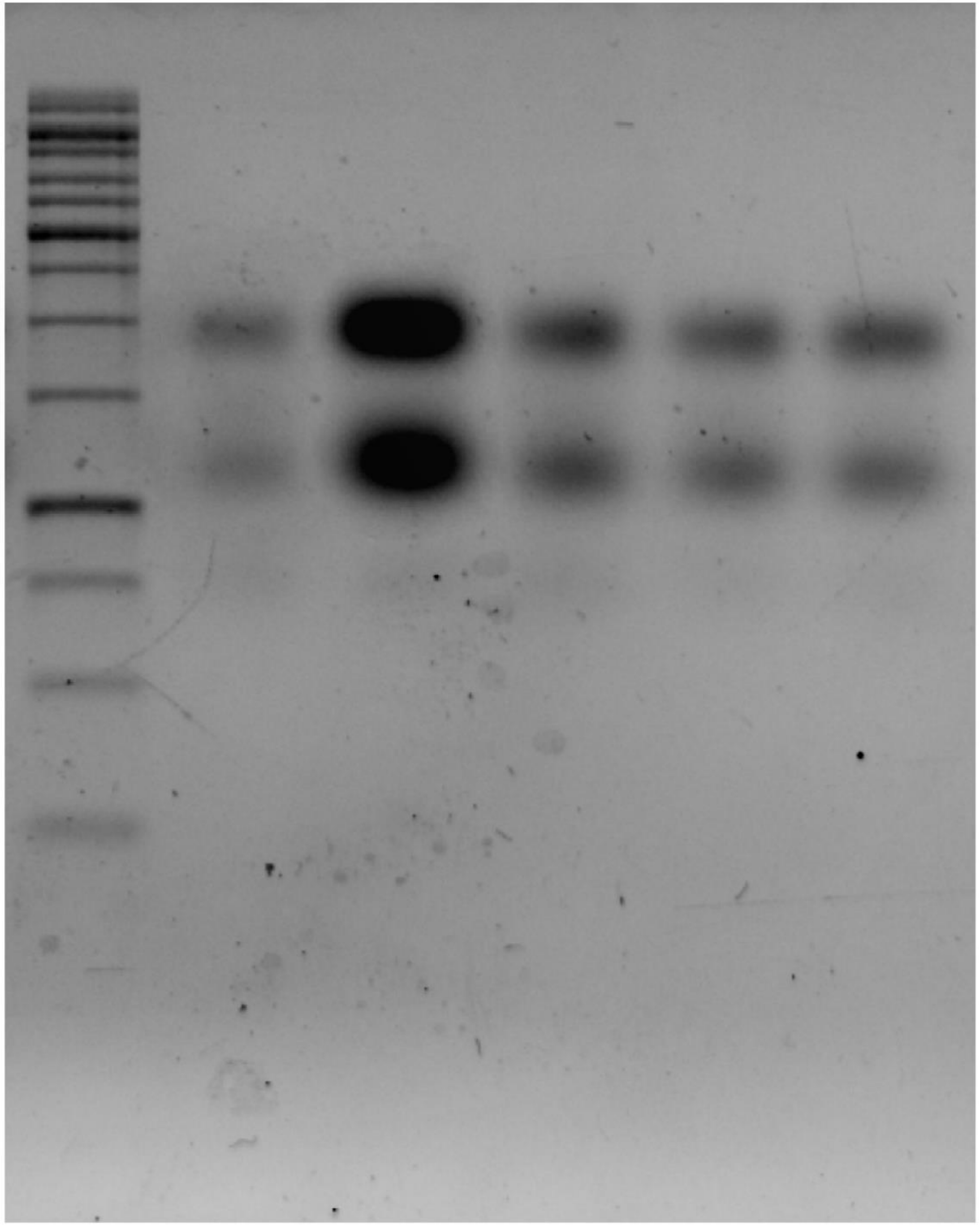

PKA

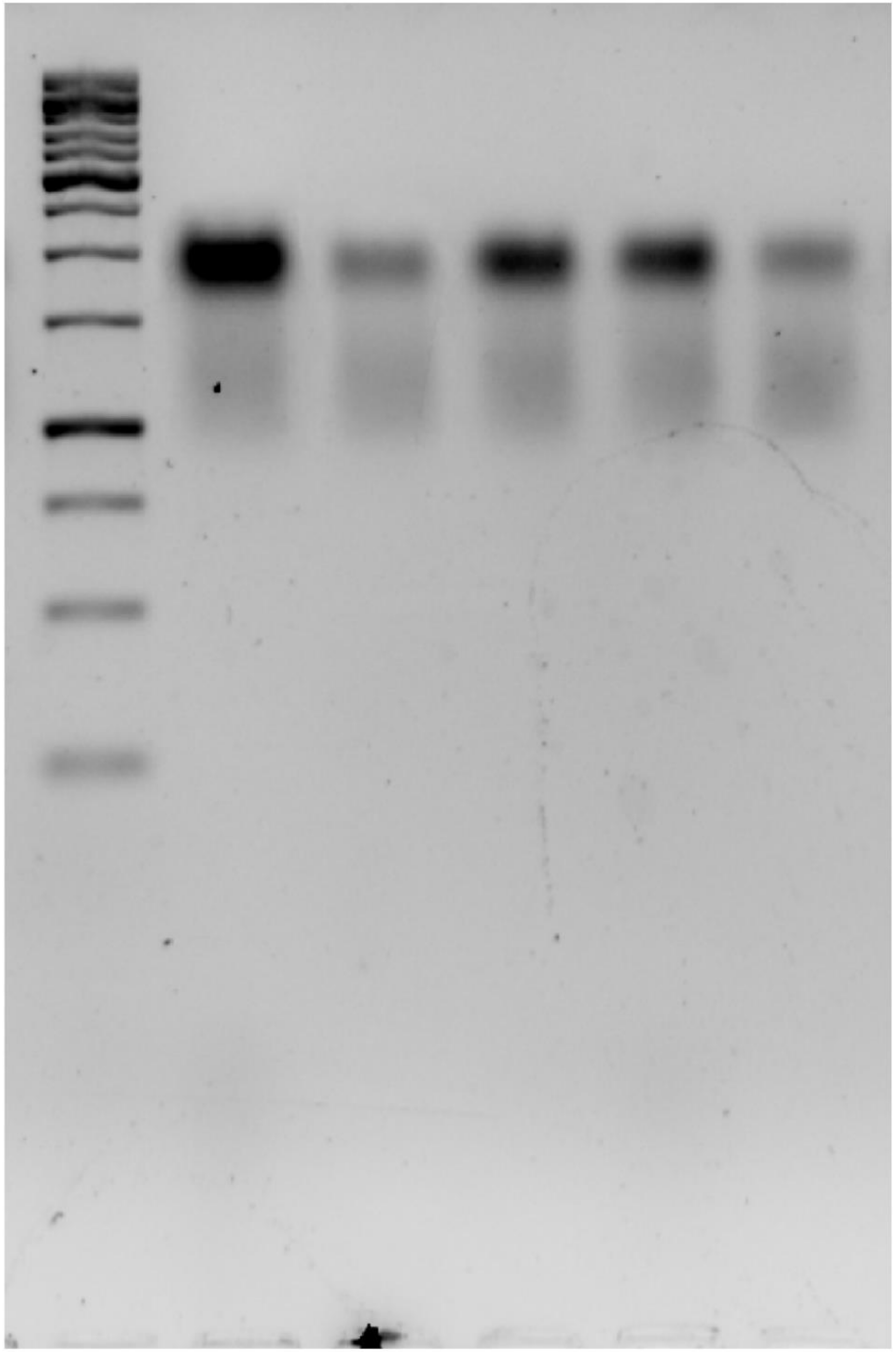

p-GSK3β

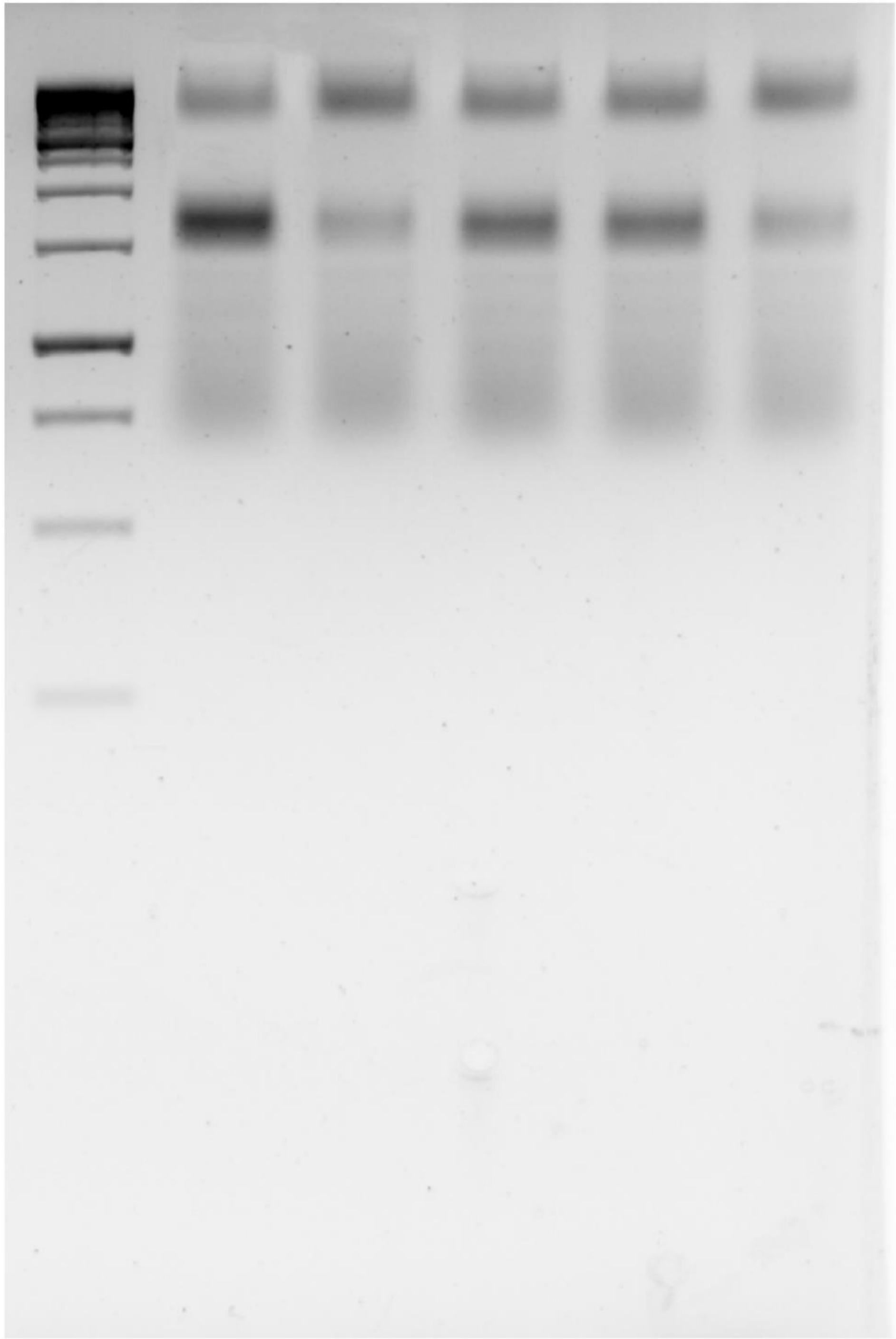

p-GSK3β

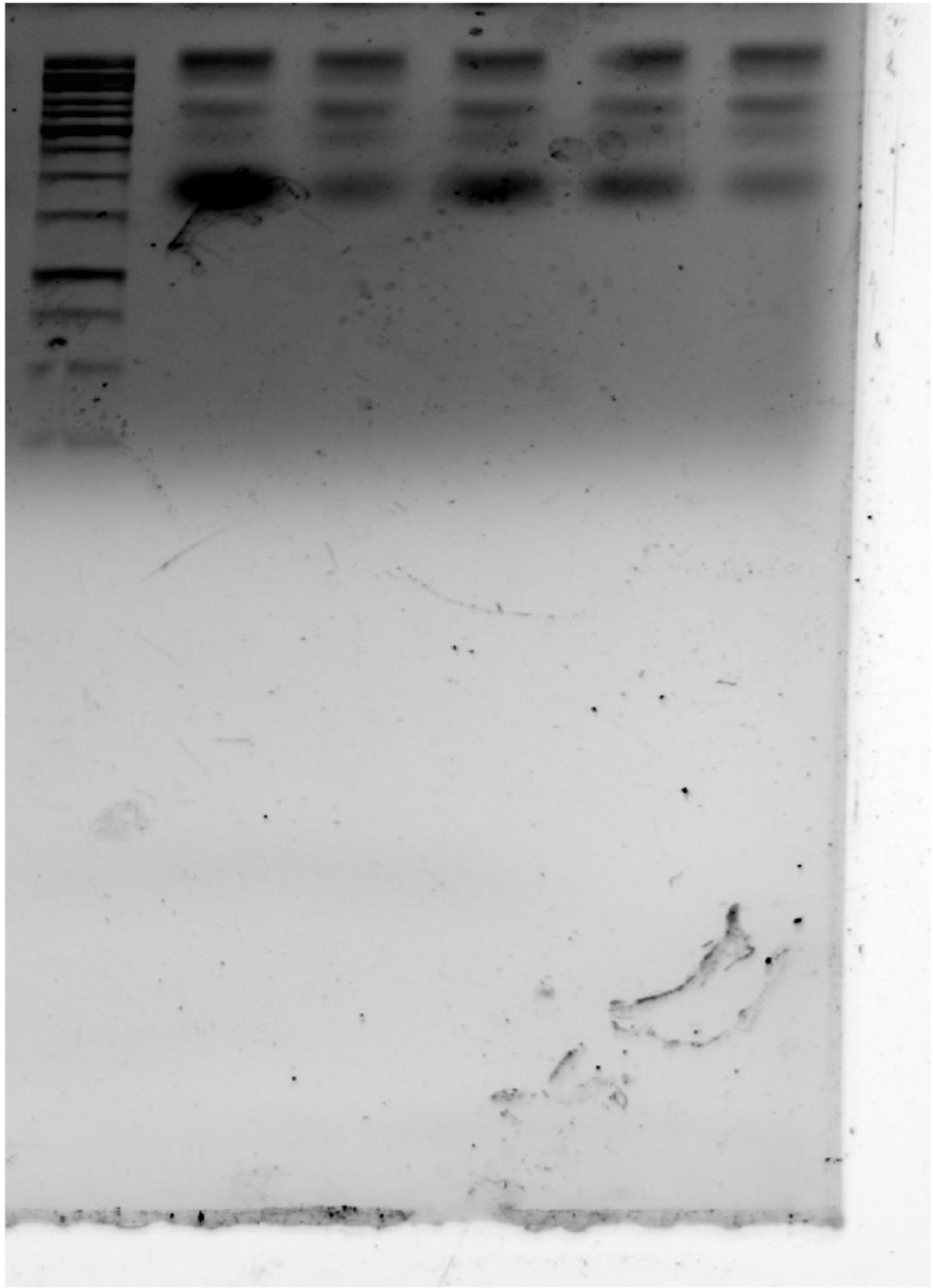

p-GSK3β

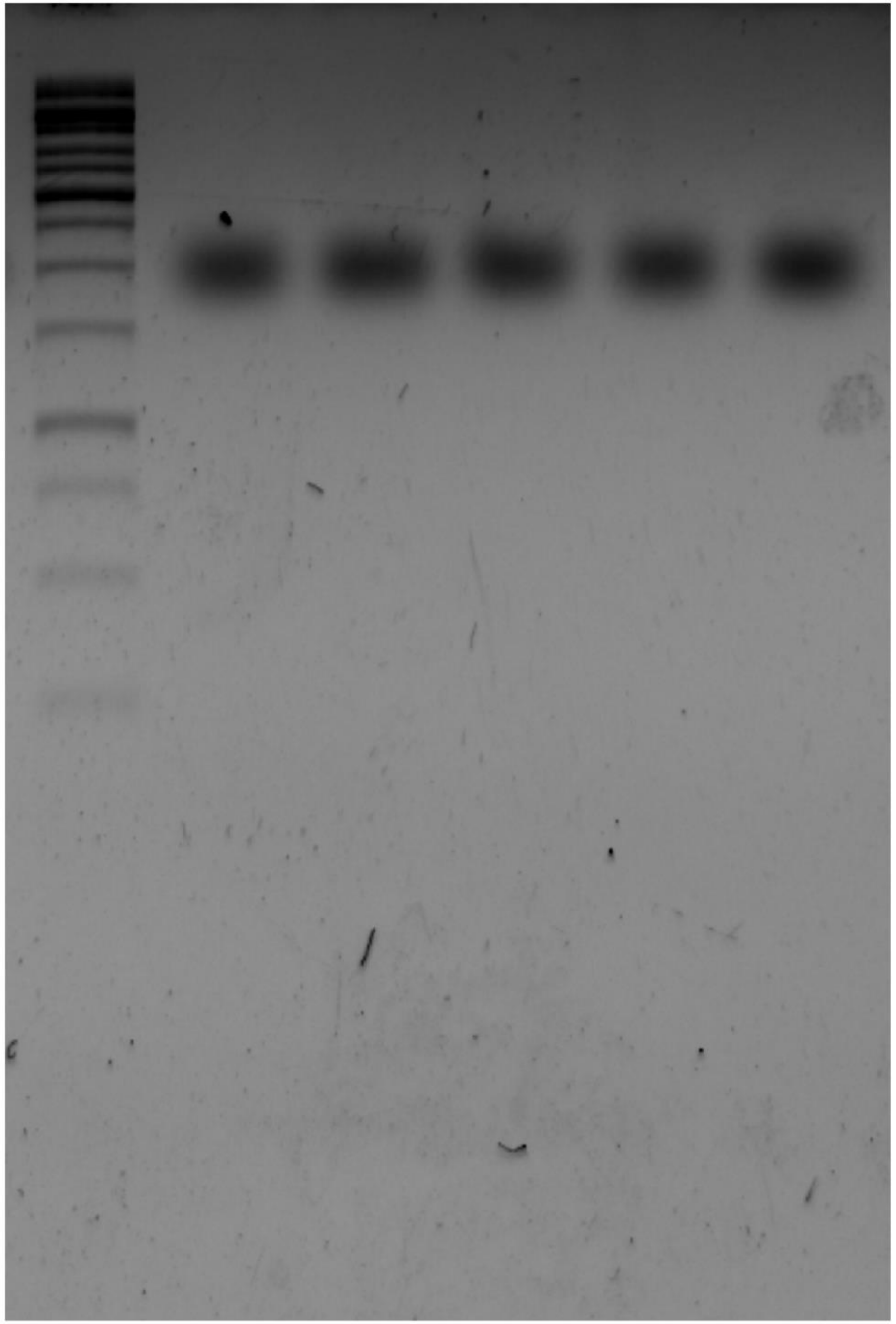

BETA ACTIN

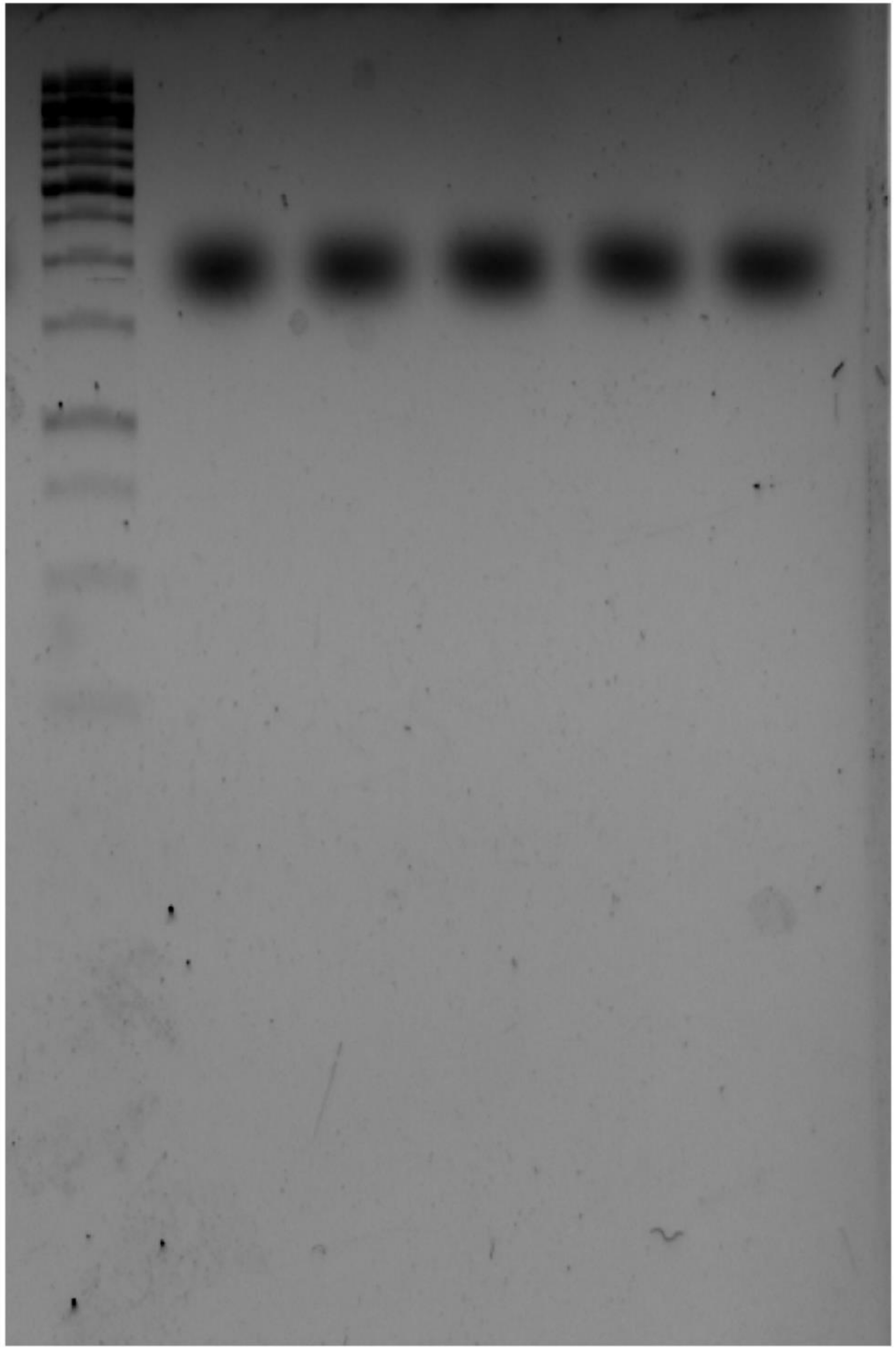

BETA ACTIN

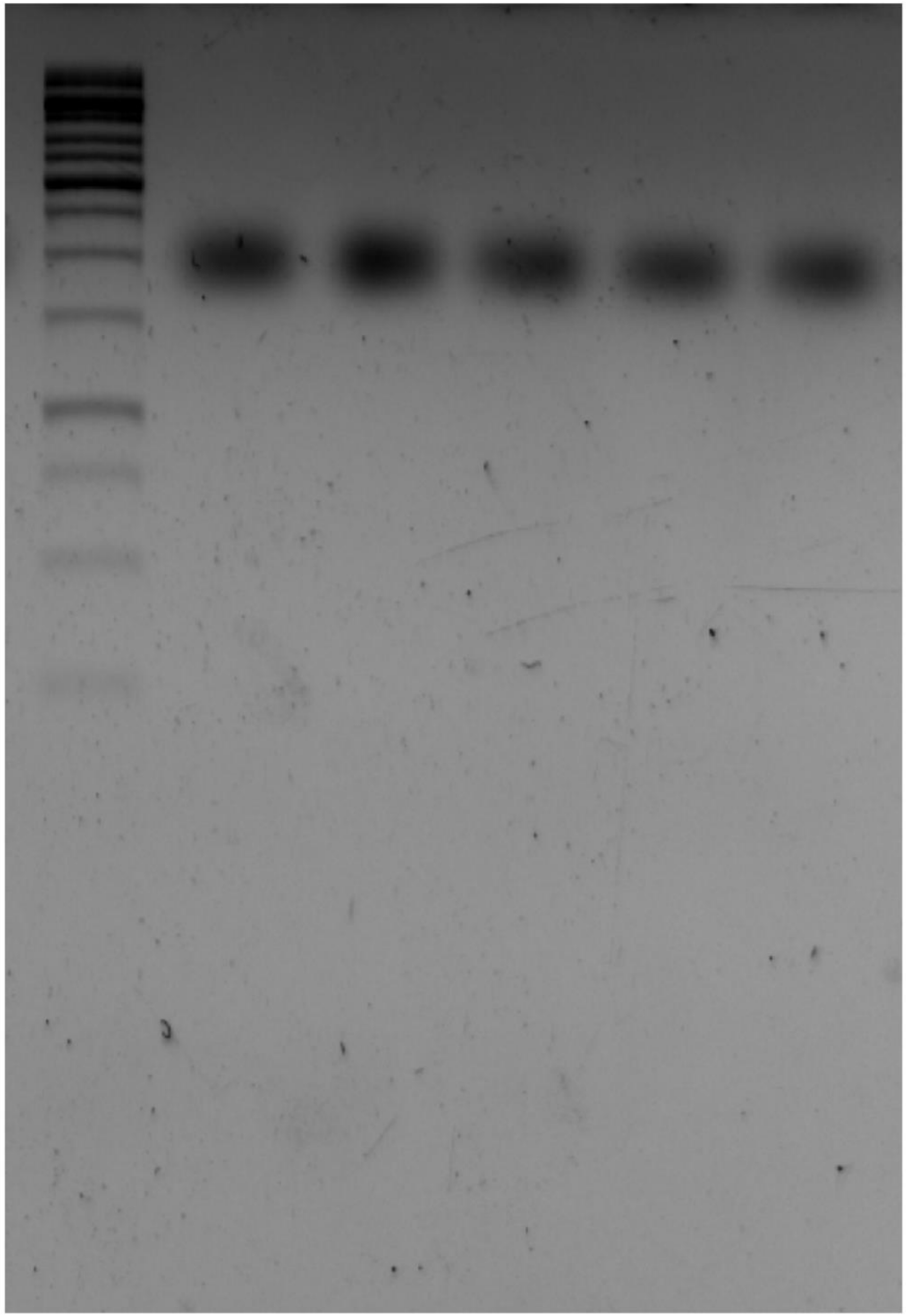

BETA ACTIN

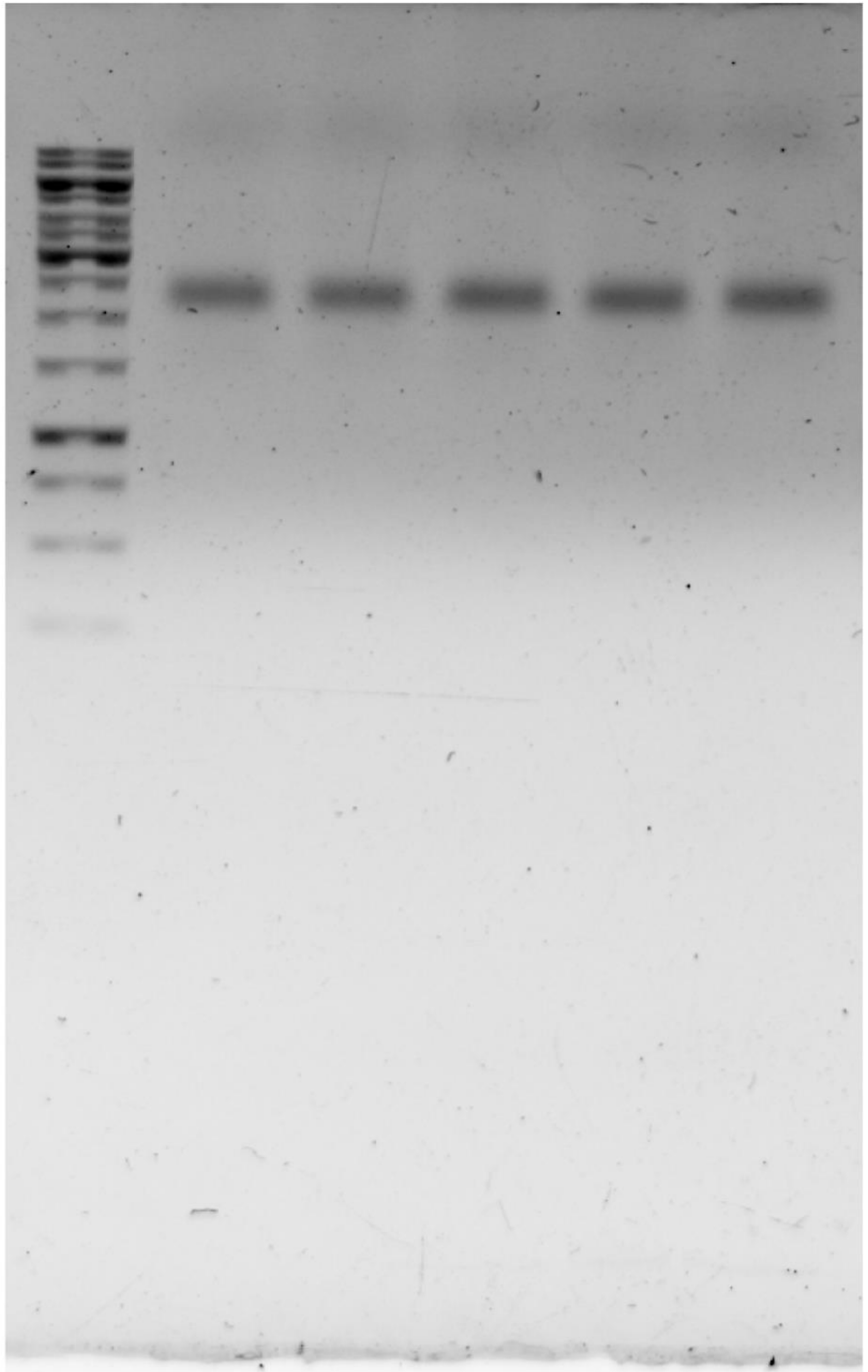

T-AKT

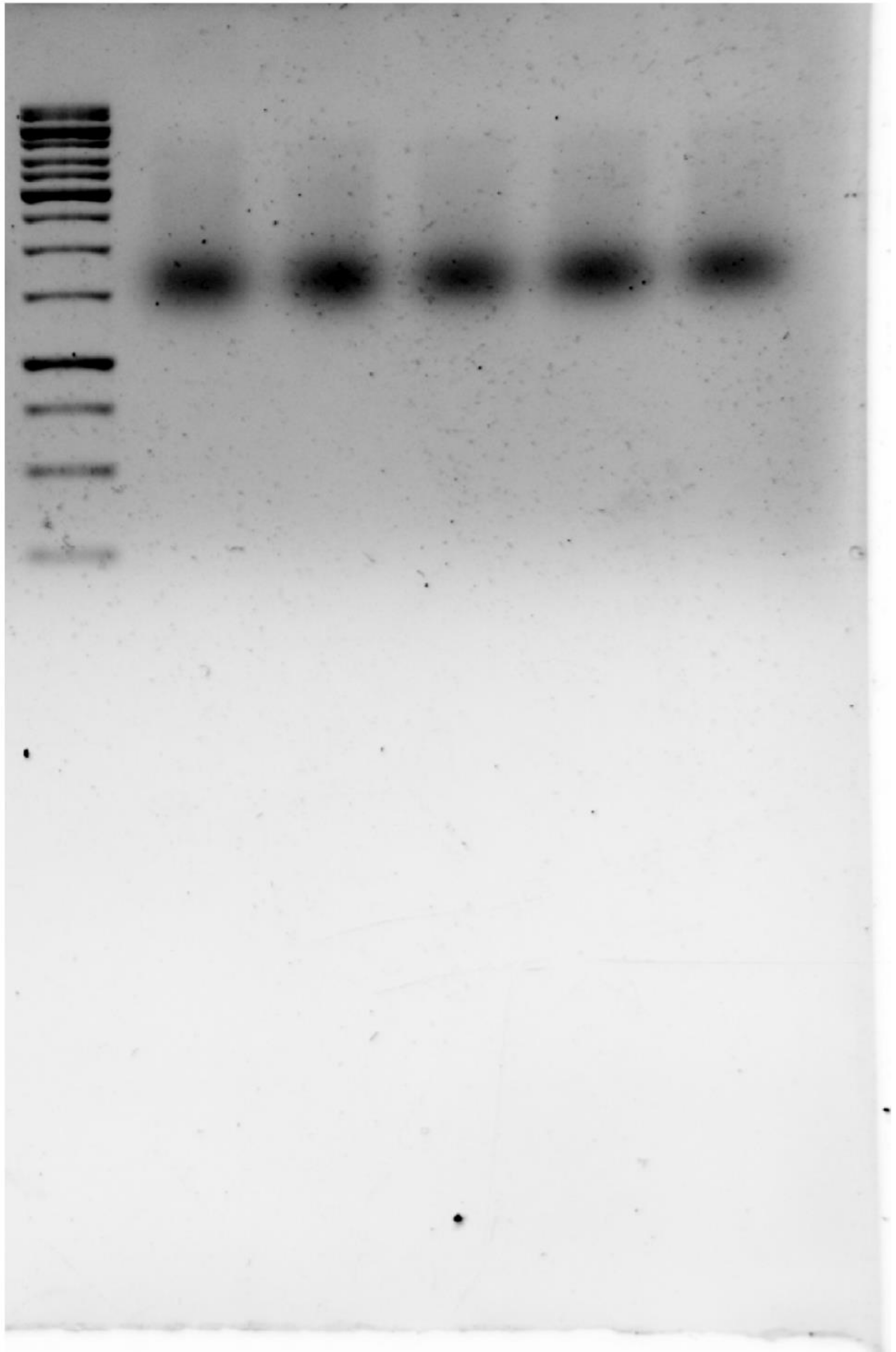

T-AKT

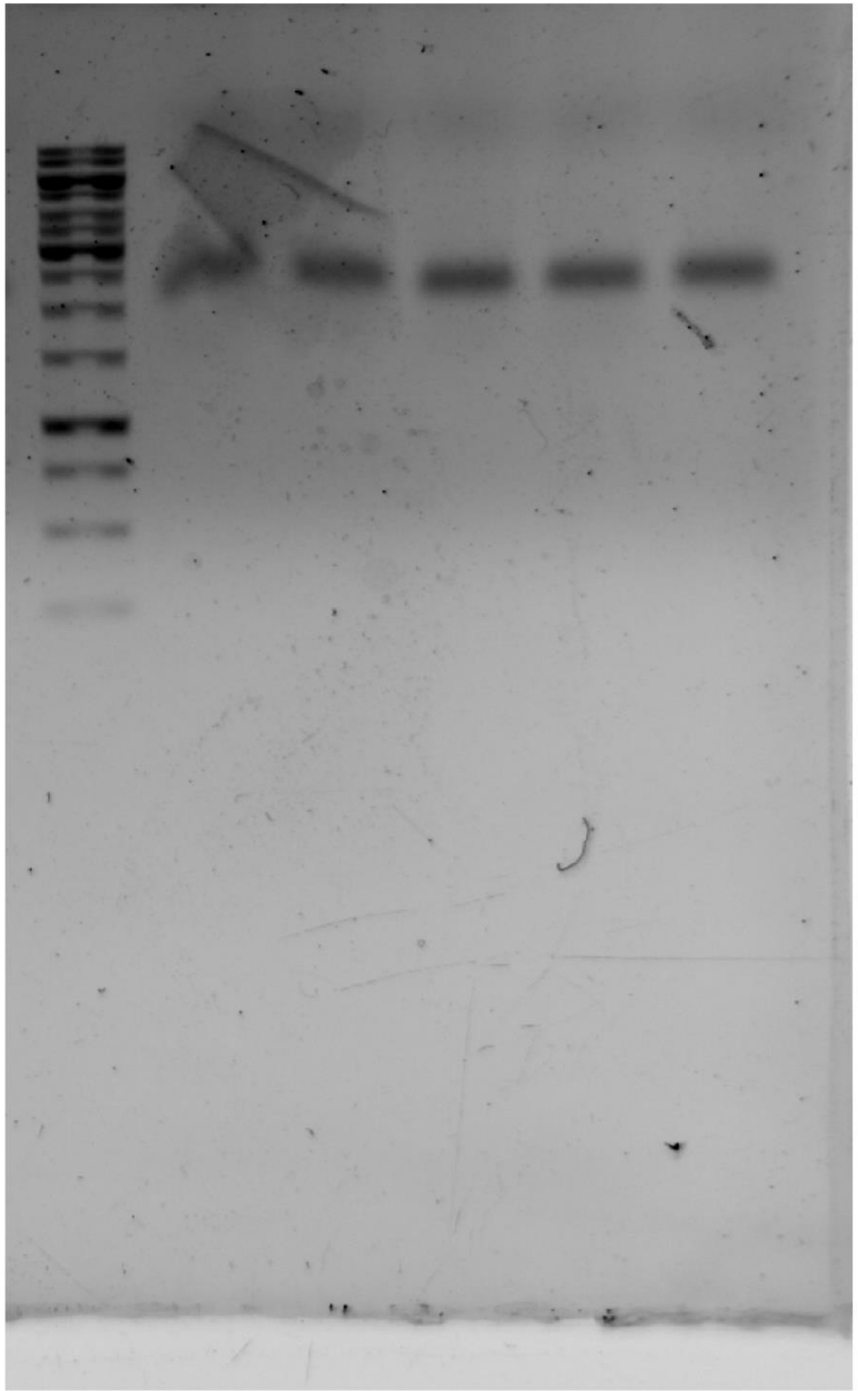

T-AKT
